# Cu–Ce Dual–Atom Sites Embedded in Zeolites Boost Resistance to Impurity Interference for Environmental Catalysis

**DOI:** 10.1002/anie.202517918

**Published:** 2025-10-11

**Authors:** Yanqi Chen, Penglu Wang, Wenqiang Qu, Yongjie Shen, Ya Tang, Edoardo Mariani, Yongbo Ni, Xiaonan Hu, Fuli Wang, Jin Zhang, Dengchao Peng, Xue Ding, Ming Xie, Yuejin Li, Emiliano Cortes, Dengsong Zhang

**Affiliations:** ^1^ International Joint Laboratory of Catalytic Chemistry State Key Laboratory of Advanced Special Steel Innovation Institute of Carbon Neutrality Department of Chemistry, College of Sciences Shanghai University Shanghai 200444 P.R. China; ^2^ BASF Environmental Catalyst and Metal Solutions Iselin NJ 08830 USA; ^3^ Nanoinstitute Munich, Faculty of Physics Ludwig‐Maximilians‐Universität (LMU) 80539 Munich Germany; ^4^ Department of Chemistry University of Toronto 80 St. George Street Toronto ON M5S 3H6 Canada; ^5^ Institute for Chemical Reaction Design and Discovery (WPI‐ICReDD) Hokkaido University Sapporo 001–0021 Japan; ^6^ Department of Chemical Engineering University of Bath Bath BA27AY UK

**Keywords:** Dual‐atom sites, Environmental catalysis, Impurity interference, Resistance, Zeolite

## Abstract

Biodiesel, a carbon‐neutral alternative to fossil fuels, plays a vital role in decarbonizing transportation, with global production exceeding 40 million tons annually. However, its widespread use introduces elevated phosphorus and metal cations into vehicle exhaust, severely deactivating Cu‐SSZ‐13 catalysts for NO_X_ reduction through pore blockage, framework degradation, and Cu sites loss. We present a Cu–Ce dual‐atom catalyst embedded in SSZ‐13 that maintains high performance in ammonia‐selective catalytic reduction under phosphorus‐rich conditions. Ce species, precisely positioned in eight‐membered rings, displace P‐sensitive [ZCu^2+^OH]^+^ sites, enriching the catalyst with P‐tolerant Z_2_Cu^2^⁺ species in six‐membered rings. Concurrent Ce─P interactions restore the electronic environment of Cu sites, enhancing NH_3_/NO adsorption and redox cycling. This design sustains 90% NO_X_ conversion and 100% N_2_ selectivity at 210 °C, even after phosphorus exposure. The strategy is broadly applicable to impurity‐sensitive environmental reactions, including NH_3_ oxidation and the coupled removal of NO_X_ with VOCs, offering a practical pathway to durable, poison‐resistant catalysts for clean and sustainable mobility.

## Graphical abstract

Embedding Cu‐Ce dual‐atom sites in zeolites enhances catalyst resistance to impurity poisoning for environmental applications. By implanting Ce into Cu‐SSZ‐13, we increase the relative proportion of P‐insensitive Z_2_Cu^2^⁺ sites by forming [ZCe^3+^(OH)_2_]^+^ sites in the 8MRs, while Ce sites preferentially bind phosphorus. This dual function boosts phosphorus tolerance even after hydrothermal aging, offering a general strategy for robust emission control catalysts in challenging conditions.

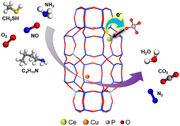

## Introduction

To reduce the environmental impact of mobile vehicles, sustainable fuels like biodiesel are increasingly used as alternatives to the petroleum‐based fuels.^[^ ^1^ ^]^ Biodiesel is widely viewed as a carbon‐neutral fuel because the CO_2_ released from its combustion is compensated for by the CO_2_ absorbed by the plants from which it is derived. Biodiesel consists of monoalkyl esters of long‐chain fatty acids, primarily extracted from animal fats, vegetable oils and/or recovered greases through commercial transesterification processes.^[^ ^2^ ^]^ However, it is important to note that biodiesel fuels typically contain trace amounts of alkali metals (Na and K), alkali earth metals (Ca and Mg), and phosphorus (P). These elements originate from the homogeneous catalysts (Na and K) used in transesterification, hard water washing (Mg and Ca), and the feedstock itself (P). The alkali and alkaline earth metals, along with elevated levels of phosphorus from biodiesel combustion, are known to significantly affect the service life of exhaust emission control catalysts.^[^ ^3^ ^]^

Zeolites, a class of microporous materials with unique molecular sieving properties, include Cu‐SSZ‐13 with a chabazite (CHA) structure. This zeolite possesses Z_2_Cu^2^⁺ sites located in six‐membered rings (6MRs) and [ZCu^2+^OH]^+^ sites located in eight‐membered rings (8MRs), endowing it with significant commercial application value for selective catalytic reduction of nitrogen oxides (NO*_x_*) in mobile vehicles exhaust gas treatment.^[^ ^4^ ^]^ Unfortunately, phosphorous is a strong poison for any catalyst, including Cu‐SSZ‐13, and in a conventional diesel engine it is present coming from the lubricating oil. Biodiesel‐powered vehicles have the added disadvantage of containing P impurities also in the fuel itself, which would cause a much more severe catalyst deactivation compared to the conventional ones.^[^ ^5^ ^]^ Specifically, current literature indicates that phosphorous in combustion exhaust gases predominantly exists as gas‐phase H_3_PO_4_. During its transit through an exhaust system, H_3_PO_4_ would react with NH_3_ to form (NH_4_)_2_HPO_4_, which is likely to be the primary phosphorus species that would reach the selective catalytic reduction (SCR) catalyst. The consequences of phosphorus poisoning over a Cu‐SSZ‐13 catalyst include pores blockage in zeolites by phosphorus‐containing species and compounds, framework dealumination, and loss of the exchanged Cu^2+^ sites caused by Al or Cu interaction with phosphorus.^[^ ^6^ ^]^

One strategy to restore catalyst activity after it is poisoned by phosphorus is to wash the poisoned catalyst with hot water to dissolve water‐soluble phosphorus compounds. Another strategy involves antagonistic interactions between different impurities to slow down the deactivation effect.^[^ ^7^ ^]^ Nevertheless, hot water washing would increase vehicle operating cost due to catalyst disassembly, assembly and installation, and cause service interruptions.^[^ ^7^ ^]^ The preferential binding between phosphorus and Zn, K, or Na metal impurities in vehicle exhaust could shield the active Cu sites of Cu‐SSZ‐13 catalyst from interacting with phosphorus. However, the effectiveness of this antagonistic effect largely hinges on the relative abundance of the counterions to form phosphate compounds stoichiometrically.^[^ ^8^ ^]^ Furthermore, the combined hydrothermal aging, required for system durability simulation, and the phosphorus poisoning effect would exacerbate catalyst deactivation. Therefore, an effective strategy to address the phosphorus poisoning problem for NO*_x_* reduction catalysts, especially that suitable for hydrothermally aged catalysts, is highly desirable for vehicles emission control.

Herein, we report a novel solution to the aforementioned problem by precise positioning Cu and Ce dual‐atom sites in SSZ‐13 at respectively targeted locations, to obtain the structure referred as Ce‐Cu‐SSZ‐13. By exchanging Ce into a Cu‐SSZ‐13 catalyst, the new type of [ZCe^3+^(OH)_2_]^+^ species in the 8MRs are precisely positioned, which increase the relative proportion of Z_2_Cu^2+^ sites that are not prone to phosphorus poisoning in the active Cu sites. Taking the selective catalytic reduction of NO*_x_* with NH_3_ as a probing reaction, the such obtained Ce‐Cu‐SSZ‐13 catalyst demonstrates exceptional phosphorous‐resistance for the NH_3_‐SCR reaction, achieving 90% NO*_x_* conversion and 100% N_2_ selectivity at 210 °C after phosphorus exposure. Extensive comparative studies involving experimental work and theory calculations confirm that Cu–Ce dual‐atom sites in SSZ‐13 zeolites have effectively minimized the P‐sensitive [ZCu^2+^OH]^+^ sites in the 8MRs and maximized the P‐insensitive Z_2_Cu^2+^ sites in the 6MRs. Meanwhile, the phosphorus‐resistant catalytic reduction mechanism over P‐insensitive active [ZCu^2+^OH]^+^ sites are also further clarified. Practically, this work provides a promising methodology to enhance phosphorus‐resistance of Cu‐zeolite based SCR catalysts, which is especially suitable for emission reduction from biodiesel‐powered vehicles. Moreover, this innovative strategy of embedding Cu–Ce dual‐atom sites in zeolites also proposes new insights to obtain new catalysts for a plethora of applications possessing high resistance against poisoning from impurities.

## Results and Discussion

### Catalytic Structure Design and Performance

**Figure 1:**
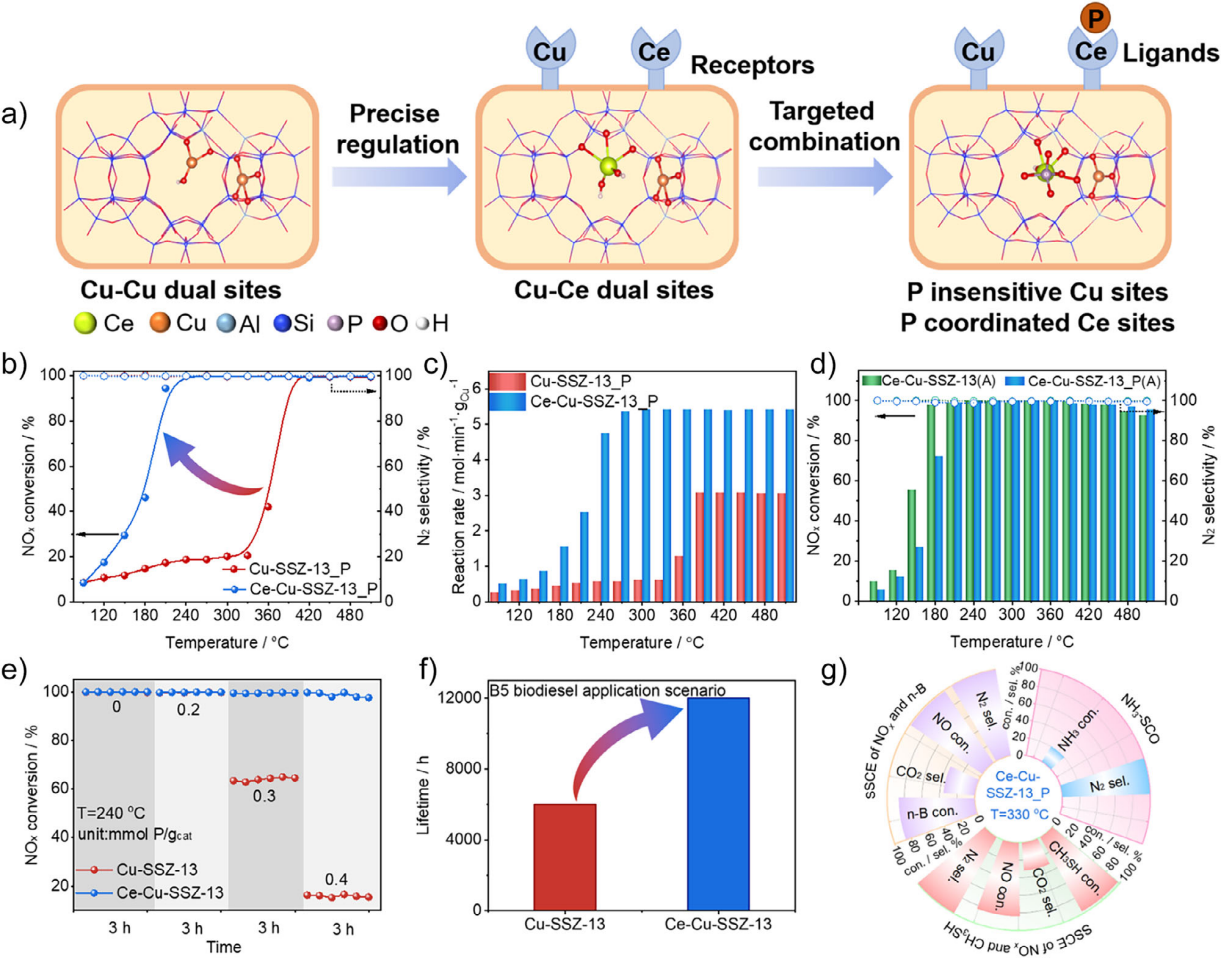
Catalytic structure design and performance. a) Schematic diagram of the Cu‐Ce dual‐atom zeolite catalyst. b) NO*_x_* conversion and N_2_ selectivity of Cu‐SSZ‐13_P and Ce‐Cu‐SSZ‐13_P in NH_3_‐SCR. c) Cu‐normalized reaction rate over Cu‐SSZ‐13_P and Ce‐Cu‐SSZ‐13_P. d) NO*_x_* conversion and N_2_ selectivity of Ce‐Cu‐SSZ‐13(A) and Ce‐Cu‐SSZ‐13_P(A) in NH_3_‐SCR. e) Stability test of Cu‐SSZ‐13 and Ce‐Cu‐SSZ‐13 loaded with different levels of phosphorus at 240 °C. f) Lifetime prediction of Cu‐SSZ‐13 and Ce‐Cu‐SSZ‐13 in the B5 biodiesel fuel (5% biodiesel) application scenario. g) Performance evaluation of Ce‐Cu‐SSZ‐13_P in other environmental catalysis reactions (NH_3_‐SCO, SSCE of NO*_x_* and n‐B, SSCE of NO_x_ and CH_3_SH) at 330 °C.

Strategically introducing Ce into Cu‐SSZ‐13 catalyst to construct Cu–Ce dual‐atom sites allows us to convert the P‐sensitive [ZCu^2+^OH]^+^ sites in the 8MRs to the P‐insensitive Z_2_Cu^2+^ sites in the 6MRs through the formation of [ZCe^3+^(OH)_2_]^+^ species in the 8MRs. As shown in Figure 1a, in this structure, Ce selectively binds with phosphorous, thus keeping it away from the active Cu sites. The Ce‐Cu‐SSZ‐13_P (where the “_P” suffix indicates a sample completely poisoned with P) catalyst was obtained as a result of an extensive investigation involving multiple factors including phosphorus loading, type of secondary cation, and concentration of Ce(NO_3_)_3_ solution required for ion exchange (Figures S1–S7). Figure 1b illustrates the catalytic activity of Cu‐SSZ‐13_P and Ce‐Cu‐SSZ‐13_P. Ce‐Cu‐SSZ‐13_P maintains NO*_x_* conversion above 90% when the reaction temperature exceeds 210 °C, indicating that atomic Ce sites significantly mitigate the detrimental effects of P on Cu‐SSZ‐13. Compared with currently reported P‐tolerant NH_3_‐SCR catalysts, the Ce‐Cu‐SSZ‐13_P catalyst exhibits the most favorable performance (Table S1). It is noteworthy that the N_2_ selectivity remains 99% for Cu‐SSZ‐13_P and Ce‐Cu‐SSZ‐13_P, suggesting phosphorus effectively inhibits the occurrence of nonselective oxidation side reactions involving NH_3_ (Figure 1b). Ce‐Cu‐SSZ‐13 exhibits significantly superior phosphorus resistance compared to physical mixed CeO_2_ and Cu‐SSZ‐13, underscoring the critical role of atomically dispersed Ce sites (Figures S8 and S9). Additionally, the reaction rates of Cu‐SSZ‐13_P and Ce‐Cu‐SSZ‐13_P, normalized by their respective copper contents obtained via inductively coupled plasma optical emission spectrometry (ICP‐OES), align with NH_3_‐SCR performance evaluation results (Figure 1c, Table S2). Within the low‐temperature range (below 200 °C), Ce‐Cu‐SSZ‐13‐P exhibits a similar normalized reaction rate to Cu‐SSZ‐13, indicating that Ce and P in Ce‐Cu‐SSZ‐13‐P do not change the turnover frequency of the SCR catalysis (Figure S10).

To simulate the catalyst durability under real operating conditions, we further evaluate the NO_x_ removal efficiency of Ce‐Cu‐SSZ‐13 and Ce‐Cu‐SSZ‐13_P catalysts after hydrothermal aging treatment (650 °C, 50 h, 10 vol.%H_2_O). Figure 1d indicates that Ce‐Cu‐SSZ‐13 (A) (A represents the sample after hydrothermal aging treatment) catalyst owns remarkable activity above 200 °C both before and after phosphorous treatment, with Ce‐Cu‐SSZ‐13_P(A) having slightly lower activity than Ce‐Cu‐SSZ‐13(A) below 200 °C. Further, an on‐stream stability test is performed at 240 °C on fresh and phosphorus‐poisoned Cu‐SSZ‐13 and Ce‐Cu‐SSZ‐13 catalysts as a function of phosphorus level in Figure 1e, revealing that Ce‐Cu‐SSZ‐13 exhibits excellent phosphorus resistance. Based on test results, we anticipate Ce‐Cu‐SSZ‐13 will demonstrate a service life of over 12 000 h under B5 biodiesel fuel conditions, markedly exceeding commercial Cu‐SSZ‐13 (Figures S11 and 1f). In addition, regeneration of the fully deactivated Ce‐Cu‐SSZ‐13 via mild acid washing or hydrothermal treatment partially restores its performance by reconstructing the electronic and coordination structures of the Cu/Ce sites (Figures S12–S14). For scalable application, we assess key parameters (Si/Al ratios, Ce introduction methods, and cost), confirming the approach's effectiveness and universality (Figures S15–S19). To further evaluate the resistance of Ce‐Cu‐SSZ‐13 against a variety of environmental catalysis that also are poisoned by phosphorus, we extend its application to NH_3_ selective catalytic oxidation (NH_3_‐SCO), selective synergistic catalytic elimination (SSCE) of NO*_x_* and CH_3_SH, and SSCE of NO*_x_* and n‐Butylamine (n‐B) in Figure 1g. The performance of Ce‐Cu‐SSZ‐13_P surpasses that of Cu‐SSZ‐13_P at 330 °C, involving higher NH_3_ conversion and N_2_ selectivity in NH_3_‐SCO reaction, boosted CH_3_SH/NO_x_ conversion and CO_2_/N_2_ selectivity in SSCE of NO_x_ and CH_3_SH, as well as increased n‐B/NO_x_ conversion and CO_2_/N_2_ selectivity in SSCE of NO_x_ and n‐Butylamine (Figure S20). The performance evaluation of Ce‐Cu‐SSZ‐13_P within a wide temperature range and various environmental catalytic reactions consistently demonstrate its exceptional resistance to impurity interference in many types of applications (Figures S21–S25).

### Structure Characterization

**Figure 2:**
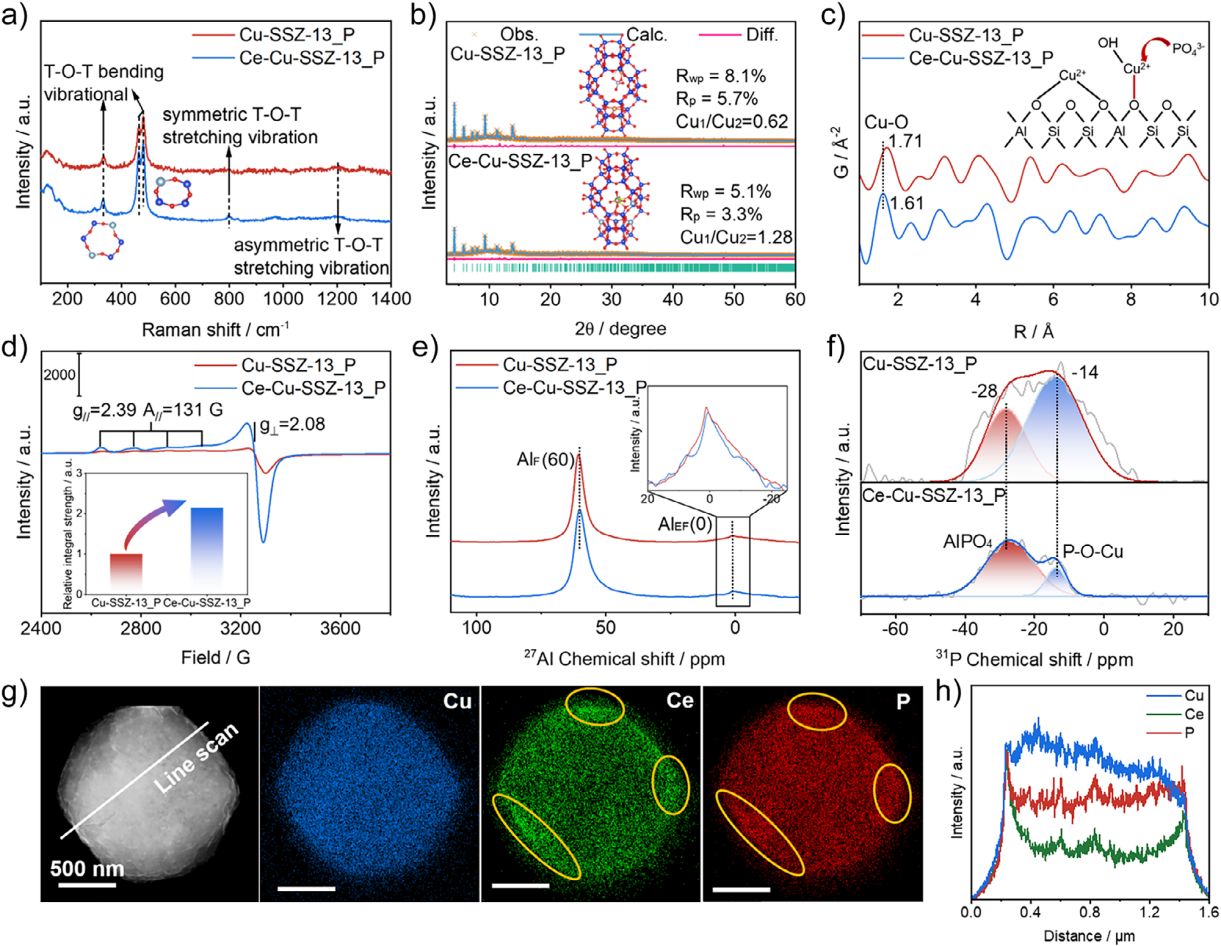
Crystal structure and morphology. a) Raman spectra of Cu‐SSZ‐13_P and Ce‐Cu‐SSZ‐13_P. b) Crystallographic structure of Cu‐SSZ‐13_P, Ce‐Cu‐SSZ‐13_P and their corresponding final Rietveld refinement results by using synchrotron X‐ray scattering data. Orange, blue, pink, and green represent the observed curve, calculated curve, difference curve, and Bragg peak (*λ* = 6.889 nm) respectively. c) Calculated PDF G(r) for Cu‐SSZ‐13_P and Ce‐Cu‐SSZ‐13_P. d) EPR diagram and integral diagram of the Cu‐SSZ‐13_P and Ce‐Cu‐SSZ‐13_P (*T* = 100 K). e) ^27^Al SSNMR of Cu‐SSZ‐13_P and Ce‐Cu‐SSZ‐13_P. f) ^31^P SSNMR of Cu‐SSZ‐13_P and Ce‐Cu‐SSZ‐13_P. g) HR‐TEM‐EDS mapping results of Cu, Ce, and P elements in Ce‐Cu‐SSZ‐13_P. h) Line scan results of Cu, Ce and P elements in Ce‐Cu‐SSZ‐13_P.

The structure and crystallinity of Cu‐SSZ‐13_P and Ce‐Cu‐SSZ‐13_P were determined by Raman and X‐ray diffraction (XRD). As shown in Figure 2a, Raman spectra show five bands associated with the skeletal vibrations of Cu‐SSZ‐13.^[^ ^9^ ^]^ The T‐O‐T (T is Si or Al) bending vibrational modes of 6MRs in the chabazite (CHA) structure, along with T‐O‐T bending vibrational modes of the tetrahedral ring (4MRs) within the hexagonal column and CHA cage, exhibits peaks at 330, 465, and 480 cm^−1^ respectively.^[^ ^10^ ^]^ The bands that belong to typical symmetric and asymmetric T‐O‐T stretching vibration modes appear at 800 and 1200 cm^−1^, respectively.^[^ ^11^ ^]^ These bands for Ce‐Cu‐SSZ‐13_P appear to show higher intensity than Cu‐SSZ‐13_P, indicating that Ce sites protects the Cu‐SSZ‐13 skeleton from being damaged by P. Although Cu‐SSZ‐13_P and Ce‐Cu‐SSZ‐13_P share the same CHA structure (PDF#47–0762) (Figures S26 and S27), XRD results also support this conclusion.^[^ ^7^, ^12^ ^]^ The fact that the bands associated with Cu‐O‐Cu is not produced in the Raman spectra by using 532 nm laser excitation and that no CuO*_x_* peaks are observed in the XRD patterns indicates that Cu exists mainly as cations in the zeolite structure.^[^ ^13^ ^]^

Rietveld refinement of synchrotron XRD were used to pinpoint the positions and proportion of Cu and Ce atoms in Cu‐Ce dual atom zeolite catalysts. The refinement results with a lower *R*_wp_ value show that Cu sites occupy both 6MRs and 8MRs, while Ce sites mainly occupy 8MRs (Tables S3–S6). As shown in Figure 2b, the introduction of phosphorus reduces the [ZCu^2+^OH]^+^/Z_2_Cu^2+^ (Cu_1_/Cu_2_) ratio in Cu‐SSZ‐13 from 2.96 to 0.62, which suggests the phosphorus preferentially bonds with the [ZCu^2+^OH]^+^ located in the 8MRs (Figure S28). Introducing Ce into the structure reduces the Cu_1_/Cu_2_ ratio to about one half (2.96→1.33), suggesting that Ce altered the type and location of active Cu^2+^ sites by occupying the 8MRs. The results of hydrogen‐temperature‐programmed reduction (H_2_‐TPR) provide strong support for this conclusion (Figure S29). Moreover, the Cu_1_/Cu_2_ ratio in the Ce‐Cu‐SSZ‐13_P (1.28) is much close to that in the Ce‐Cu‐SSZ‐13 (1.33), indicating that atomic Ce sites effectively hinders the phosphorus from poisoning the [ZCu^2+^OH]^+^ sites (Figure S30). The pair distribution function (PDF) G(r) analysis was used to further probe the coordination of the zeolite catalysts. Cu‐SSZ‐13_P and Ce‐Cu‐SSZ‐13_P exhibit similar framework structures but the average Cu─O distance in Ce‐Cu‐SSZ‐13_P (1.61 Å) is shorter than that in Cu‐SSZ‐13_P (1.71 Å) (Figure 2c). Apparently, the introduction of Ce sites in the presence of phosphorus enhances the interaction between the active Cu^2+^ sites and the framework oxygen in the catalyst.^[^ ^14^ ^]^

Electron paramagnetic resonance (EPR), ^27^Al solid‐state nuclear magnetic resonance (^27^Al SSNMR), and ^31^P SSNMR analyze the variations of Cu, Al, and P species in Cu‐SSZ‐13_P and Ce‐Cu‐SSZ‐13_P. EPR signal at g_⊥ _= 2.08 is attributed to Z_2_Cu^2+^ and [ZCu^2+^OH]^+^, and the EPR signal at g_// _= 2.39 is attributed to the Z_2_Cu^2+^ located at 6MRs (Figure 2d).^[^ ^15^ ^]^ In addition, the quadratic integral area of EPR curve indicates that Ce‐Cu‐SSZ‐13_P owns more highly active Cu^2+^ species, consistent with its elevated NH_3_‐SCR performance. In Figure 2e, the ^27^Al SSNMR results reveal an exceptionally strong peak at approximately 60 ppm for tetrahedral aluminum within the zeolite framework.^[^ ^16^ ^]^ Cu‐SSZ‐13_P and Ce‐Cu‐SSZ‐13_P molecular sieve frameworks exhibit localized de‐alumination (peak at ∼0 ppm), indicating that Ce has no detrimental effect on the stability of framework aluminum. Figure 2f reveals two peaks at ‐14 and ‐28 ppm in ^31^P SSNMR spectra, assignable to phosphorus species in both Cu‐SSZ‐13_P and Ce‐Cu‐SSZ‐13_P. The peak intensity of the P─O─Cu bond at δ‐14 ppm diminishes with the introduction of Ce, indicating a weak interaction between phosphorus and Cu. The peak at δ‐28 ppm is usually attributed to the tetrahedron P (4‐coordination) in P(OAl)_4_, indicating the formation of aluminum phosphate phase in the zeolite cages.^[^ ^8^, ^17^ ^]^ Similarly, H_2_‐TPR further confirms the formation of Cu‐PO_3_^−^/PO_4_^3^^−^ and AlPO_4_ phase in Cu‐SSZ‐13_P and Ce‐Cu‐SSZ‐13_P, respectively (Figure S29). The morphology and structure of Cu‐SSZ‐13_P and Ce‐Cu‐SSZ‐13_P were characterized using high‐resolution transmission electron microscope (HR‐TEM). All catalysts have a cubic structure with similar shape and dimensions (Figures 2g and S31). Typical lattice fringes of the Cu_3_(PO_4_)_2_ (111) and Cu_3_(PO_4_)_2_ (002) crystal planes are observed on Cu‐SSZ‐13_P (Figure S32). On Ce‐Cu‐SSZ‐13_P, lattice fringes corresponding to the CePO_4_(012) and CePO_4_(111) crystal planes are observed, suggesting that Ce preferentially binds with the phosphorus species (Figure S33). Energy dispersive spectrum analysis (EDS) shows that the elements Al, Si, and O are highly dispersed in Cu‐SSZ‐13_P crystals, while Cu and P appear uniformly as aggregates on the surface, which is consistent with the results of aberration‐corrected high‐angle annular dark‐field scanning transmission electron microscopy (HAADF‐STEM) measurement (Figures S31 and S34). Note, Cu and P track each other in the aggregates formed on Cu‐SSZ‐13_P, indicating a certain affinity between them. However, when Ce is introduced into the Cu‐SSZ‐13_P zeolite, as shown in Figure 2g and 2h, Cu distribution becomes highly uniform and P and Ce display similar distributions (as indicated by the yellow circles in the picture).

**Figure 3:**
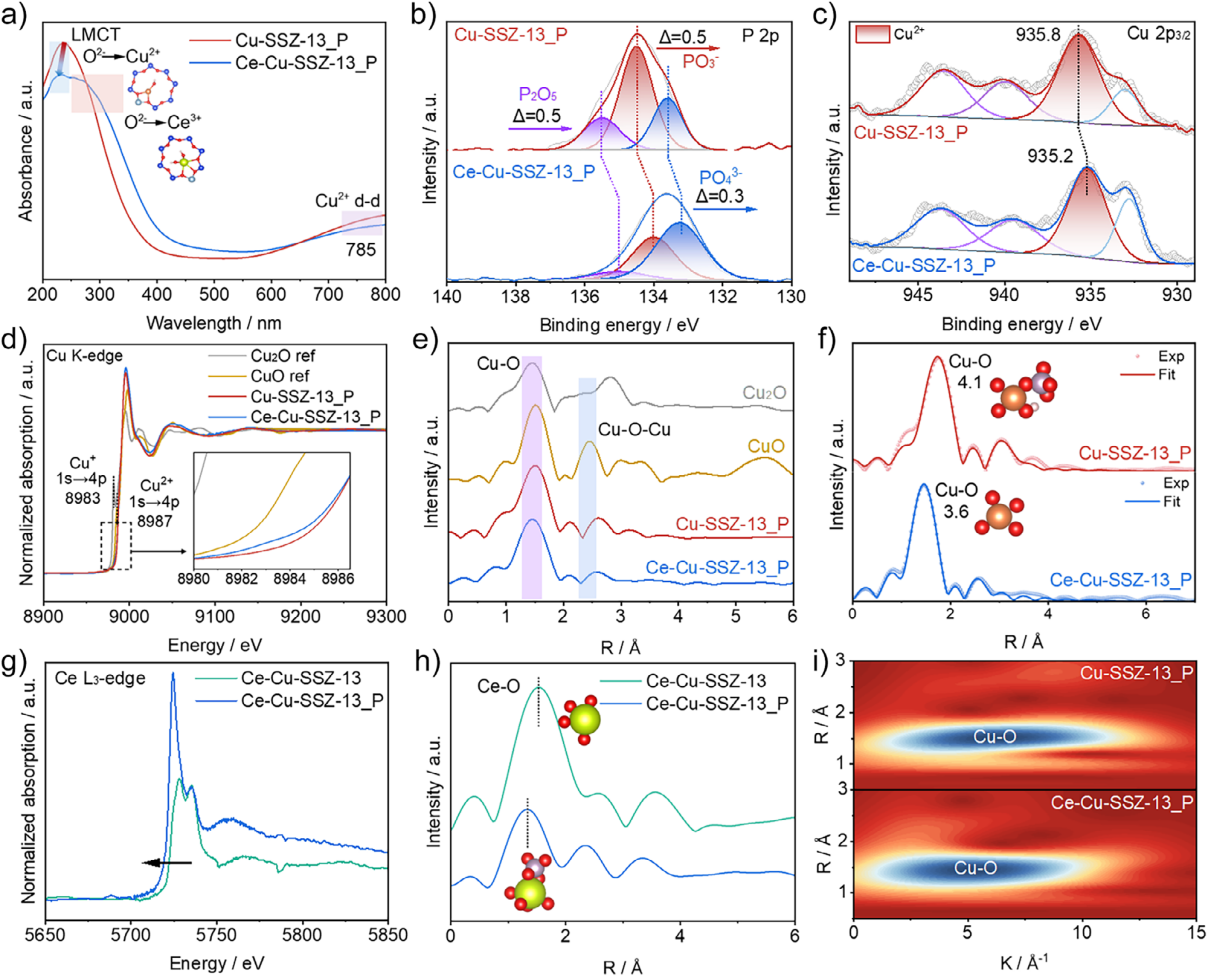
Electronic structure. a) UV–vis spectra of Cu‐SSZ‐13_P and Ce‐Cu‐SSZ‐13_P. b) P 2p XPS of Cu‐SSZ‐13_P and Ce‐Cu‐SSZ‐13_P. c) Cu 2p_3/2_ XPS of Cu‐SSZ‐13_P and Ce‐Cu‐SSZ‐13_P. d) Cu K‐edge XANES of Cu‐SSZ‐13_P, Ce‐Cu‐SSZ‐13_P, Cu_2_O, and CuO. e) The FT‐EXAFS curves in R space of Cu‐SSZ‐13_P, Ce‐Cu‐SSZ‐13_P, Cu_2_O, and CuO. f) FT‐EXAFS fitting curves for Cu‐SSZ‐13_P and Ce‐Cu‐SSZ‐13_P in R space. g) Ce L_3_‐edge XANES of Ce‐Cu‐SSZ‐13 and Ce‐Cu‐SSZ‐13_P. h) FT‐EXAFS curves for Ce‐Cu‐SSZ‐13 and Ce‐Cu‐SSZ‐13_P in R space. i) WT contour plots of Cu‐SSZ‐13_P and Ce‐Cu‐SSZ‐13_P.

To determine the electronic structure of catalysts, we characterized them using UV‐visible diffuse reflectance spectroscopy (UV‐Vis‐DRS) and X‐ray photoelectron spectroscopy (XPS). As shown in Figure 3a, UV–vis‐DRS of Cu‐SSZ‐13_P shows adsorption band near 240 nm, corresponding to the ligand‐to‐metal charge transfer (LMCT) between the ligand O^2−^ ions and the metal Cu^2+^, and around 785 nm, attributing to the d‐d transition of Cu^2+^ species.^[^ ^13^, ^18^ ^]^ The band center for O^2−^→Cu^2+^ undergoes blue‐shifted on Ce‐Cu‐SSZ‐13_P compared with Cu‐SSZ‐13_P, indicating that the introduction of Ce sites enhances the interaction of Cu^2+^ with the zeolite framework O species. Furthermore, a new peak observed at 292 nm in Ce‐Cu‐SSZ‐13_P originates from charge transfer between lattice oxygen and Ce^3+^ ions, suggesting that Ce exists in an ionic form.^[^ ^19^ ^]^ Figure 3b shows the P 2p XPS spectra of Cu‐SSZ‐13_P and Ce‐Cu‐SSZ‐13_P. The binding energies of P_2_O_5_, PO_3_^−^, and PO_4_^3^^−^ are located at 135.6 eV, 134.4 eV and 133.5 eV respectively in Cu‐SSZ‐13_P.^[^ ^20^ ^]^ For Ce‐Cu‐SSZ‐13_P, the binding energies of these phosphorus species shift to lower values (by 0.5, 0.5 and 0.3 eV, respectively), indicating electron transfer from Ce to P. This is consistent with the observation from Ce 3d XPS experiment, which shows a significantly higher Ce^3+^ binding energy in Ce‐Cu‐SSZ‐13_P than in Ce‐Cu‐SSZ‐13 (Figure S35). The main peak in the Cu 2p_3/2_ XPS spectra of Cu‐SSZ‐13_P at 935.8 eV is assigned to Cu^2+^, while the peaks between 940.0 to 947.5 eV represent satellite peaks of Cu^2+^ (Figure 3c).^[^ ^21^ ^]^ The characteristic peak of Cu^2+^ in Ce‐Cu‐SSZ‐13_P exhibits reduced binding energy relative to Cu‐SSZ‐13_P but remains unchanged from that of Cu‐SSZ‐13, suggesting that Ce restores the electronic environment around Cu^2+^ sites by neutralizing the electron‐withdrawing effect on of P (Figure S36).

To probe the electronic and coordinative environment of the catalyst, we conducted X‐ray absorption fine structure (XAFS) spectra. The Cu K‐edge X‐ray absorption near‐edge structure (XANES) spectra displayed in Figure 3d shows that the Cu‐SSZ‐13_P and Ce‐Cu‐SSZ‐13_P samples present a lack of characteristic electron transfer peaks in the 1s→4p orbital transition for Cu^+^ and Cu^2+^, compared to the reference samples (CuO and Cu_2_O). In addition, the position of the absorption edge for Ce‐Cu‐SSZ‐13_P locate between 8980 and 8988 eV slightly shift toward lower energy compared with Cu‐SSZ‐13_P, indicating that the introduction of Ce sites inhibits the loss of electrons on Cu.^[^ ^22^ ^]^ It is noteworthy that the absorption edge energies of Cu‐SSZ‐13_P and Ce‐Cu‐SSZ‐13_P exhibit a shift toward higher energies (compared to the reference samples Cu_2_O and CuO), indicating that Ce and P influences the electronic and valence state of Cu. As shown in Figure 3e, fourier‐transformed extended X‐ray absorption fine structure (FT‐EXAFS) curves in R space display the coordination structures of Cu–O and Cu–O–Cu in the zeolite and reference samples.^[^ ^23^ ^]^ Accordingly, the fitting curves of Cu‐SSZ‐13_P and Ce‐Cu‐SSZ‐13_P in R‐space show Cu─O peaks at approximately 1.95 Å in Figure 3f.^[^ ^24^ ^]^ In addition, the Cu─O coordination number for Ce‐Cu‐SSZ‐13_P is 3.6, which is slightly lower than 4.1 observed in Cu‐SSZ‐13_P, owing to the diminished Cu─O─P bonding caused by Ce introduction (Table S7). Moreover, compared to Ce‐Cu‐SSZ‐13, the Ce L_3_‐edge XANES spectrum of Ce‐Cu‐SSZ‐13_P shows a lower energy of the white line peak, indicating that electrons on Ce are more inclined to transfer from Ce to P via Ce─O─P bonding (Figure 3g). In Figure 3h, the FT‐EXAFS in R space further elucidates the local coordination surrounding Ce atoms. Both Ce‐Cu‐SSZ‐13 and Ce‐Cu‐SSZ‐13_P show a prominent Ce‐O peak at about 1.8 Å, indicating that Ce exists in the ionic form (Figure S37).^[^ ^25^ ^]^ Meanwhile, the wavelet transform (WT) contour plots in Figure 3i reveal only Cu─O bonds without other ligand species, indicating that Cu^2+^ ions is dispersed in the zeolite.^[^ ^24^ ^]^

### Adsorption Behavior and Molecular Dynamics

**Figure 4:**
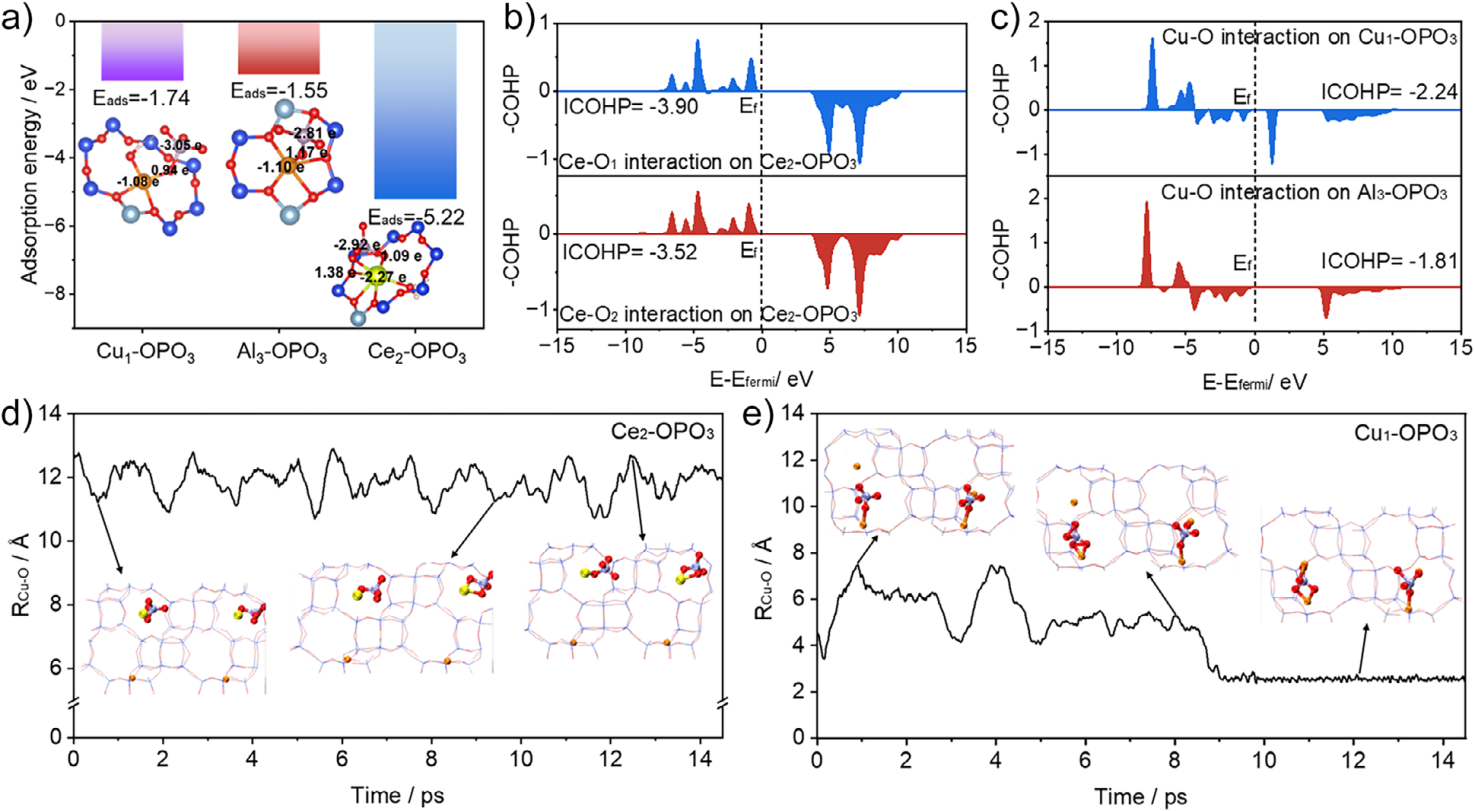
Adsorption behavior and molecular dynamics. a) The adsorption energy of PO_4_^3^^−^ on Cu_1_‐OPO_3_, Al_3_‐OPO_3_, Ce_2_‐OPO_3_, and bader charges of Cu, P, O, Ce in [ZCu^2+^OH]^+^ at 8MRs, with Z_2_Cu^2+^ at 6MRs and [ZCe^3+^(OH)_2_]^+^ at 8MRs, respectively. b) and c) The COHP of Cu─O/Ce─O bond for PO_4_^3−^ adsorption on Ce‐Cu‐SSZ‐13 and Cu‐SSZ‐13. d) and e) Calculated time‐dependent distance between the PO_4_^3^^−^ and Cu sites in the structures of Ce_2_‐OPO_3_ and Cu_1_‐OPO_3_ in AIMD simulation. Schematics and snapshot structures taken from the AIMD simulation of PO_4_^3^^−^ inter‐cage diffusion is presented in the insets.

The adsorption behavior of phosphorus species (PO_4_^3^^−^) in Cu‐SSZ‐13 and Ce‐Cu‐SSZ‐13 containing Cu^2+^, Al^3+^, and Ce^3+^ sites were investigated by density functional theory (DFT) calculation. In the absence of Ce^3+^ sites, PO_4_^3^^−^ interacts with both the active Cu sites and framework Al in the zeolite. For the Z_2_Cu^2+^ sites within 6MRs, only the Cu terminus interacting with PO_4_^3^^−^(Cu_2_‐OPO_3_) exhibits an adsorption energy of −1.57 eV. Conversely, for [ZCu^2+^OH]^+^ sites within 8MRs, PO_4_^3−^ can adsorb at three distinct sites: the H terminus (H‐OPO_3_), Cu/H terminus (Cu_1_(H)‐OPO_3_), and the Cu terminus (Cu_1_‐OPO_3_). The corresponding adsorption energies are −0.89, −1.44, and −1.74 eV. In addition, PO_4_^3^^−^ can also adsorb onto the Al^3+^ sites of both 8MRs and 6MRs, with adsorption energies of −1.41 eV (Al_1_‐OPO_3_), −1.47 eV (Al_2_‐OPO_3_), and −1.55 eV (Al_3_‐OPO_3_), respectively. In the presence of Ce^3+^ sites, the adsorption of PO_4_^3^^−^ is notably enhanced at the [ZCe^3+^(OH)_2_]^+^ sites within the 8MRs, which exhibits the highest adsorption energy of ‐5.22 eV (Ce_2_‐OPO_3_) (Figures S38 and S39). As shown in Figure 4a, the magnitude of adsorption energy for the most stable adsorption model of phosphorus at each site is in the following order: Ce^3+^ > Cu^2+^ > Al^3+^. In addition, we have performed bader charge calculations for the three structures, and the results show that the electrons are transferred from Cu/Ce to O in PO_4_^3^^−^. The Ce atom carries the most negative charge of −2.27 eV, indicating that [ZCe^3+^(OH)_2_]^+^ at the 8MRs position has the strongest adsorption capacity for PO_4_^3^^−^. Figure 4b and 4c examine the adsorption strength of PO_4_^3^^−^ toward Cu‐SSZ‐13 and Ce‐Cu‐SSZ‐13 via the crystal orbital Hamilton population (COHP). A more negative integrated COHP (ICOHP) value signifies greater adsorption stability and stronger bonding.^[^ ^26^ ^]^ Compared to the ICOHP of −2.24 and −1.81 eV for Cu‐O in the Cu_1_‐O‐PO_3_ and Al_3_‐O‐PO_3_ structures, respectively, the more negative ICOHP value of Ce–O in the Ce_2_‐OPO_3_ structure indicates that PO_4_^3^^−^ exhibits stronger adsorption capacity on Ce‐Cu‐SSZ‐13.

The inter‐cage diffusion behavior of PO_4_^3^^−^ in zeolite catalysts was further investigated by molecular dynamics (AIMD) simulation and shown in Figure 4d and 4e. During the AIMD simulation time, PO_4_^3^^−^ is anchored near the [ZCe^3+^(OH)_2_]^+^ sites in the 8MRs and remains far away from the Cu^2+^ sites in the cage of Ce‐Cu‐SSZ‐13 (Figure S40 and Movie S1). In contrast, PO_4_^3^^−^ diffuses between the cages and is stabilized near the Cu^2+^ sites after 14 ps in the Cu‐SSZ‐13 (Movie S2).^[^ ^27^ ^]^ In addition, the radial distribution function (RDF) reveals a significantly higher probability of finding PO_4_^3^^−^ near 2.5 Å from the Cu^2+^ sites in Cu‐SSZ‐13 than in Ce‐Cu‐SSZ‐13 (Figure S41). However, the RDF calculation shows the highest R value for Ce–O at 3 Å, indicating the occurrence of Ce–O–P (Figure S42).^[^ ^28^ ^]^ All of the above results demonstrate that Ce incorporation is able to precisely regulates the type of Cu sites from P‐sensitive [ZCu^2+^OH]^+^ in the 8MRs to P‐insensitive Z_2_Cu^2+^ in the 6MRs by filling the 8MRs with [ZCe^3+^(OH)_2_]^+^ species that would spontaneously bind PO_4_^3^^−^ and thereby well protect the active Cu^2+^ sites.

### Reaction Mechanism

**Figure 5:**
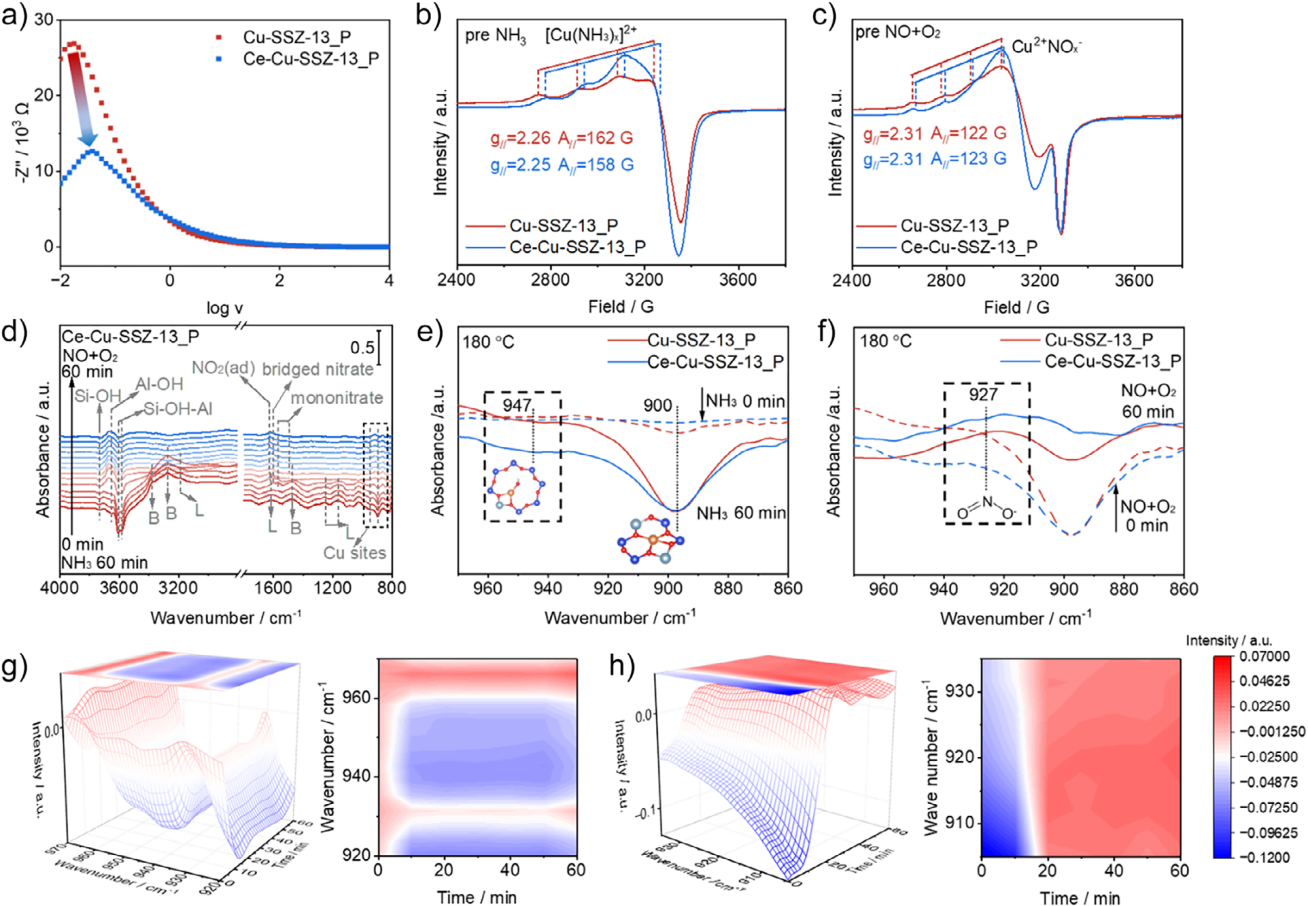
Reaction mechanism. a) EIS of Cu‐SSZ‐13_P and Ce‐Cu‐SSZ‐13_P. b) and c) *Quasi* in situ EPR spectra recorded at room temperature and the low‐field hyperfine structures of Cu‐SSZ‐13_P and Ce‐Cu‐SSZ‐13_P followed by adsorption of NH_3_/Cu and NO + O_2_/Cu atom. d) in situ DRIFTs of the transient reactions between pre‐adsorbed NH_3_ and NO + O_2_ as a function of time over Ce‐Cu‐SSZ‐13_P. Local amplification spectrum of the in situ DRIFTs e) after NH_3_ saturation and f) subsequent NO/O_2_ reaction on Cu‐SSZ‐13_P and Ce‐Cu‐SSZ‐13_P. g) and h) Three‐dimensional schematic of in situ DRIFTs on Ce‐Cu‐SSZ‐13_P near 947 and 927 cm^−1^ (upward peaks represent generated products, and downward peaks represent consumption of reactants).

To understand the difference in Cu ion mobility between Cu‐SSZ‐13_P and Ce‐Cu‐SSZ‐13_P, we performed an analysis using electrochemical impedance spectroscopy (EIS). The resonance peak of Ce‐Cu‐SSZ‐13_P shifts towards higher frequencies relative to Cu‐SSZ‐13_P, indicating that enhanced Cu ion mobility leads to a shorter dielectric relaxation time (Figure 5a).^[^ ^27^, ^29^ ^]^ *Quasi* in situ EPR, NH_3_ temperature programmed desorption coupled with a mass spectrometer (NH_3_‐TPD‐MS) and NO + O_2_ temperature programmed desorption coupled with a mass spectrometer (NO + O_2_‐TPD‐MS) were employed to study the adsorption of reactant molecules on catalysts. As shown in Figure 5b, the ultrafine‐region EPR parameters of the Ce‐Cu‐SSZ‐13_P catalyst pre‐adsorbed with NH_3_ are g_//_ = 2.25 and A_//_ = 158 G, exhibiting significant differences compared to the ultrafine‐region EPR parameters g_//_ = 2.39 and A_//_ = 131 G of the catalyst without NH_3_ adsorption. This change arises from the emergence of EPR‐active Cu(II)‐NH_3_ complexes ([Cu(NH_3_)_x_]^2+^).^[^ ^29^, ^30^ ^]^ The enhanced peak intensity in Ce‐Cu‐SSZ‐13_P, compared to Cu‐SSZ‐13_P, points to the role of atomic Ce sites in effectively restoring the NH_3_ solvation process of active Cu^2+^ sites at low temperatures. Based on NH_3_‐TPD‐MS integration results, the Lewis acid content of Ce‐Cu‐SSZ‐13_P is determined to be 0.91 µmol·g^−1^, much higher than that of Cu‐SSZ‐13_P (0.58 µmol·g^‐1^) (Figure S43, Table S8). Apparently, Ce modification enhances the low‐temperature activity by preserving the Lewis acid sites of Cu‐SSZ‐13 catalyst for NH_3_ adsorption. The EPR spectra after saturation of NO + O_2_ adsorption are shown in Figure 5c. The hyperfine region of the EPR parameters (g_//_ = 2.31, A_//_ = 123 G) are assigned to Cu^2+^NO_x_^−^ with peak intensities significantly higher for Ce‐Cu‐SSZ‐13_P than Cu‐SSZ‐13_P.^[^ ^30^ ^]^ Similarly, NO + O_2_‐TPD‐MS results indicate that the peak area for weakly adsorbed NO*_x_* significantly increases on the Ce‐Cu‐SSZ‐13_P. This demonstrates that Ce modification restores the NO oxidation process by enhancing the adsorption and activation capacity of NO on Cu‐SSZ‐13_P (Figure S44). These findings reveal that Ce‐Cu‐SSZ‐13_P has a higher Cu mobility and larger adsorption capacity for NH_3_ and NO, which is important in sustaining the dynamic redox cycles of the copper species during SCR catalysis.

In situ diffuse reflectance infrared Fourier transform spectroscopy (DRIFTS) was employed to probe the dynamic cycling process of Cu^2+^ sites in zeolite catalyst. in situ DRIFTS spectra of Ce‐Cu‐SSZ‐13_P after 1 h of NH_3_ pre‐treatment at 180 °C reveals distinct O─H vibrations at 3735, 3670, 3609, and 3583 cm^−1^ (Figure 5d).^[^ ^31^ ^]^ Different band locations of O─H vibrations stem from their different oxygen environments. Following interaction with Brønsted acid sites, NH_4_^+^ exhibits bands at 3332, 3274, and 1464 cm^−1^.^[^ ^32^ ^]^ Following interaction with Lewis acid sites, NH_3_ forms bands at 3183, 1620, 1260, and 1170 cm^−1^.^[^ ^24^, ^32^ ^]^ After passing NO + O_2_ for 20 min, the B‐NH_4_^+^ (NH_4_^+^ adsorbed on Brønsted acid sites) peak at 1464 cm^−1^ is converted to monodentate nitrate (1508 cm^−1^) and peaks at 3332, 3274, 1260, and 1170 cm^−1^ are consumed. In addition, flowing NO + O_2_ through the catalyst gradually transformed NH_4_^+^/NH_3_ species to the adsorbed NO_2_ (1626 cm^−1^),^[^ ^33^ ^]^ bridged nitrate (1610 cm^−1^)^[^ ^34^ ^]^ and monodentate nitrate (1575 cm^−1^) attached to the Cu^2+^ sites.^[^ ^35^ ^]^ The O─H peaks at high wavenumbers (3800–3500 cm^−1^) are not completely consumed even after flowing NO + O_2_ for 1 h, suggesting that some ammonia is still tightly bound to the hydroxyl groups. Following NH_3_ adsorption saturation, Cu‐SSZ‐13_P exhibits a significantly weaker peak than Ce‐Cu‐SSZ‐13_P, and its band at 3183 cm^−1^ vanishes. This indicates phosphorus exerts a weakening effect on the acid sites. Only a faint NO_2_ peak appeared at 1626 cm^−1^ after following 25 min of NO + O_2_ exposure, suggesting that phosphorus caused almost complete loss of activity in Cu‐SSZ‐13 (Figure S45). To explore the adsorption‐activation behavior of active Cu sites within the zeolite, we present a local amplification spectrum of the in situ DRIFTs collected after NH_3_ saturation in Figure 5e. One band at 900 cm^−1^ corresponds to the absorption peak generated by the T─O─T vibration of Z_2_Cu^2+^, the other T─O─T vibrational band at 947 cm^−1^ originates from [ZCu^2+^OH]^+^.^[^ ^34^ ^]^ Following adsorption of NH_3_ at 180 °C, the band at 947 cm^−1^ exhibits greater intensity on Cu‐SSZ‐13_P than on Ce‐Cu‐SSZ‐13_P. This indicates that the [ZCu^2+^OH]^+^ sites bound to phosphorus exhibit acidic properties, which impede the solvation process of NH_3_ towards active Cu^2^⁺ sites, and consequently inhibit the Cu^2+^→Cu^+^ reduction process. In Figure 5f, after introducing NO/O_2_ to Ce‐Cu‐SSZ‐13_P, complete consumption of NH_3_ on the two Cu^2+^ sites occurs concurrently with nitrite production (927 cm^−1^), via NO oxidation on the Cu^2+^ sites, an intermediate state for the oxidation of Cu^+^ to Cu^2+^.^[^ ^30^ ^]^ This band is less on Cu‐SSZ‐13_P, indicating that Ce alleviates the inhibitory effect of P on the oxidation process from Cu^+^ to Cu^2+^. These results are consistent with the finding of *quasi* in situ EPR. Ce‐Cu‐SSZ‐13_P was pre‐adsorbed with NO + O_2_ for 1 h at 180 °C, peaks of NO_2_ and monodentate nitrate in the adsorbed state are observed near 1626 and 1575 cm^−1^, respectively (Figure S46a).^[^ ^35^ ^]^ After the introduction of 500 ppm NH_3_, these two peaks disappear rapidly and NH_4_^+^ and NH_3_ species appear gradually. The Cu‐SSZ‐13_P catalyst exhibits no NO_x_ adsorption peak, indicating that phosphorus poisoning at low temperatures severely impairs the catalyst's adsorption of NO_x_ (Figure S46b). In addition, three‐dimensional thermograms were plotted in Figure 5g and 5h based on the positions of IR bands corresponding to the [Cu(NH_3_)_x_]^2+^ and Cu^2+^NO_x_^−^ characteristic peaks, indicating the typical redox cycling of the Cu^2+^ sites on Ce‐Cu‐SSZ‐13_P.

## Conclusion

In summary, we present a robust strategy of embedding Cu–Ce dual‐atom sites in SSZ‐13 zeolites to stabilize phosphorus‐insensitive Cu^2^⁺ species, enabling high catalytic efficiency across impurity‐sensitive environmental reactions, including NO_X_ reduction, NH_3_ oxidation, and the coupled removal of NO_X_ with CH_3_SH or n‐butylamine. Specifically, Ce introduced into the zeolite increase the relative proportion of the P‐insensitive Z_2_Cu^2+^ sites by forming [ZCe^3+^(OH)_2_]^+^ sites in the 8MRs. Meanwhile, Ce sites can also act as phosphorous receptors that preferentially capture the phosphorus poisons and thus avoid catalyst deactivation via precious protection of active Cu^2+^ sites. In addition, the charge transfer between Ce and P yields electron‐deficient Ce and electron‐rich P, which effectively restores the electronic environment around Cu^2+^ sites, thereby improved adsorption of NH_3_ and NO and more efficient redox cycling between Cu^2+^ and Cu^+^ during SCR reaction. Using this strategy, we are able to achieve high NO*_x_* conversion and N_2_ selectivity after phosphorus deposition on both fresh and hydrothermal aged Cu‐SSZ‐13 catalysts. Therefore, this work not only offers a promising methodology to mitigate the phosphorus poisoning issue for vehicle exhaust emission controls, especially for biodiesel vehicle applications, but also provides implications for environmental catalysts against impurity poisoning in new application scenarios including ammonia oxidation, synergistic catalytic elimination of nitric oxide and volatile organic compounds.

## Supporting Information

Experimental details, additional figures, and tables are compiled in the Supporting Information file. Additional references were cited within the Supporting Information.

## Conflict of Interests

E.C. is an International Consultant of the Academic Committee of the Carbon Neutrality Research Center at Shanghai University, China.

## Supporting information



Supporting Information

## Data Availability

The data that support the findings of this study are available from the corresponding author upon reasonable request.

## References

[1] A. Meijide , C. de la Rua , T. Guillaume , A. Röll , E. Hassler , C. Stiegler , A. Tjoa , T. June , M. D. Corre , E. Veldkamp , A. Knohl , Nat. Commun. 2020, 11, 1089, 10.1038/s41467-020-14852-6. 32107373 PMC7046764

[2] O. Filip , K. Janda , L. Kristoufek , D. Zilberman , Nat. Energy 2016, 1, 16169, 10.1038/nenergy.2016.169.

[3] J. Granestrand , R. Suárez París , M. Nilsson , F. Regali , L. J. Pettersson , Catalysts 2020, 10, 1439.

[4] Y. Wu , Y. Ma , Y. Wang , K. G. Rappé , N. M. Washton , Y. Wang , E. D. Walter , F. Gao , J. Am. Chem. Soc. 2022, 144, 9734–9746, 10.1021/jacs.2c01933. 35605129

[5] K. R. Landwehr , J. Hillas , R. Mead‐Hunter , A. King , R. A. O'Leary , A. Kicic , B. J. Mullins , A. N. Larcombe , Chemosphere 2023, 310, 136873, 10.1016/j.chemosphere.2022.136873. 36252896

[6] A. Wang , M. E. Azzoni , J. Han , K. Xie , L. Olsson , Chem. Eng. J. 2023, 454, 140040, 10.1016/j.cej.2022.140040.

[7] Z. Chen , C. Bian , Y. Guo , L. Pang , T. Li , ACS Catal. 2021, 11, 12963–12976, 10.1021/acscatal.1c03752.

[8] Y. Shen , W. Dong , L. Zhang , L. Wang , B. Chen , Y. Guo , W. Zhan , A. Wang , C. Ge , Y. Guo , Sep. Purif. Technol. 2024, 330, 125248, 10.1016/j.seppur.2023.125248.

[9] M. Chen , W. Zhao , Y. Wei , J. Han , J. Li , C. Sun , D. Mei , J. Yu , Nano Res. 2023, 16, 12126–12133, 10.1007/s12274-023-5500-x.

[10] Y. Zhang , H. Zhu , T. Zhang , J. Li , J. Chen , Y. Peng , J. Li , Environ. Sci. Tech. 2022, 56, 1917–1926, 10.1021/acs.est.1c06068. 34856804

[11] J. Zhang , Y. Chu , X. Liu , H. Xu , X. Meng , Z. Feng , F.‐S. Xiao , Chinese J. Catal. 2019, 40, 1854–1859, 10.1016/S1872-2067(19)63287-0.

[12] J. Wang , J. Zhang , C. Xing , T. Jin , J. Liu , M. Ju , X. Tang , Chem. Eng. J. 2023, 455, 140379, 10.1016/j.cej.2022.140379.

[13] B. Chen , R. Xu , R. Zhang , N. Liu , Environ. Sci. Tech. 2014, 48, 13909–13916, 10.1021/es503707c. 25365767

[14] M. A. Molina , A. Manjón‐Sanz , M. Sánchez‐Sánchez , Micropor. Mesopor. Mat. 2021, 319, 110973, 10.1016/j.micromeso.2021.110973.

[15] L. Ma , Y. Cheng , G. Cavataio , R. W. McCabe , L. Fu , J. Li , Chem. Eng. J. 2013, 225, 323–330, 10.1016/j.cej.2013.03.078.

[16] H. Zhao , Y. Zhao , M. Liu , X. Li , Y. Ma , X. Yong , H. Chen , Y. Li , Appl. Catal. B: Environ. 2019, 252, 230–239, 10.1016/j.apcatb.2019.04.037.

[17] K. Xie , J. Woo , D. Bernin , A. Kumar , K. Kamasamudram , L. Olsson , Appl. Catal. B: Environ. 2019, 241, 205–216, 10.1016/j.apcatb.2018.08.082.

[18] D. Jo , G. T. Park , T. Ryu , S. B. Hong , Appl. Catal. B: Environ. 2019, 243, 212–219, 10.1016/j.apcatb.2018.10.042.

[19] R. Li , Y. Liang , Z. Zhang , Q. Huang , X. Jiang , R. Yang , L. Yu , J. Jiang , Catal. Today 2022, 405–406, 125–134, 10.1016/j.cattod.2022.06.025.

[20] A. Wang , K. Xie , D. Bernin , A. Kumar , K. Kamasamudram , L. Olsson , Appl. Catal. B: Environ. 2020, 269, 118781, 10.1016/j.apcatb.2020.118781.

[21] J. Wang , Z. Peng , Y. Chen , W. Bao , L. Chang , G. Feng , Chem. Eng. J. 2015, 263, 9–19, 10.1016/j.cej.2014.10.086.

[22] Q. Gao , Z. Yan , W. Zhang , H. S. Pillai , B. Yao , W. Zang , Y. Liu , X. Han , B. Min , H. Zhou , L. Ma , B. Anaclet , S. Zhang , H. Xin , Q. He , H. Zhu , J. Am. Chem. Soc. 2023, 145, 19961–19968. 37651158 10.1021/jacs.3c06514

[23] K. Liu , H. Shen , Z. Sun , Q. Zhou , G. Liu , Z. Sun , W. Chen , X. Gao , P. Chen , Nat. Commun. 2025, 16, 1203, 10.1038/s41467-025-56534-1. 39885168 PMC11782518

[24] Q. An , G. Xu , J. Liu , Y. Wang , Y. Yu , H. He , ACS Catal. 2023, 13, 6851–6861, 10.1021/acscatal.3c01322.

[25] J. Fang , H. Bao , B. He , F. Wang , D. Si , Z. Jiang , Z. Pan , S. Wei , W. Huang , J. Phys. Chem. C 2007, 111, 19078–19085, 10.1021/jp076627m.

[26] Q. Li , Y. Hu , G. Liu , Z. Wu , X. Chen , Y. F. Song , Small 2025, 21, e2411043, 10.1002/smll.202411043. 39937148

[27] Y. Fu , W. Ding , H. Lei , Y. Sun , J. Du , Y. Yu , U. Simon , P. Chen , Y. Shan , G. He , H. He , J. Am. Chem. Soc. 2024, 146, 11141–11151, 10.1021/jacs.3c13725. 38600025

[28] T. Li , A. Naveed , J. Zheng , B. Chen , M. Jiang , B. Liu , Y. Zhou , X. Li , M. Su , R. Guo , J. Sumner , C. C. Li , Y. Liu , Angew. Chem. Int. Ed. 2025, 64, e202424095. 10.1002/anie.202424095 40084513

[29] M. Moreno‐González , B. Hueso , M. Boronat , T. Blasco , A. Corma , J. Phys. Chem. Lett. 2015, 6, 1011–1017, 10.1021/acs.jpclett.5b00069. 26262861

[30] M. Moreno‐González , R. Millán , P. Concepción , T. Blasco , M. Boronat , ACS Catal. 2019, 9, 2725–2738. 10.1021/acscatal.8b04717.

[31] Y. Shan , Y. Sun , J. Du , Y. Zhang , X. Shi , Y. Yu , W. Shan , H. He , Appl. Catal. B: Environ. 2020, 275, 119105, 10.1016/j.apcatb.2020.119105.

[32] Y. Shan , X. Shi , Z. Yan , J. Liu , Y. Yu , H. He , Catal. Today 2019, 320, 84–90, 10.1016/j.cattod.2017.11.006.

[33] C. Liu , G. Malta , H. Kubota , T. Toyao , Z. Maeno , K.‐i. Shimizu , J. Phys. Chem. C 2021, 125, 21975–21987, 10.1021/acs.jpcc.1c06651.

[34] A. Guo , K. Xie , H. Lei , V. Rizzotto , L. Chen , M. Fu , P. Chen , Y. Peng , D. Ye , U. Simon , Environ. Sci. Tech. 2021, 55, 12619–12629, 10.1021/acs.est.1c03630. 34510889

[35] D. Wang , L. Zhang , K. Kamasamudram , W. S. Epling , ACS Catal. 2013, 3, 871–881, 10.1021/cs300843k.

