# Supplementary material for: Cu–Ce Dual–Atom Sites Embedded in Zeolites Boost Resistance to Impurity Interference for Environmental Catalysis
Source: Angew Chem Int Ed Engl. 2025 Oct 11;64(49):e202517918. doi: 10.1002/anie.202517918 (PMC12668302; doi:10.1002/anie.202517918)
Supplement: Supplementary file 1 — Supporting Information [file ANIE-64-e202517918-s001.docx]

Supporting Information

**Cu-Ce Dual-Atom Sites Embedded in Zeolites Boost Resistance to Impurity Interference for Environmental Catalysis**

Yanqi Chen,^[a]^ Penglu Wang,*^, [a]^ Wenqiang Qu,^[a, d]^ Yongjie Shen,^[a, e]^ Ya Tang,^[a]^ Edoardo Mariani,^[c]^ Yongbo Ni,^[a]^ Xiaonan Hu,^[a]^ Fuli Wang,^[a]^ Jin Zhang,^[a]^ Dengchao Peng,^[a]^ Xue Ding,^[a]^ Ming Xie,^[f]^ Yuejin Li,*^, [b]^ Emiliano Cortes,*^, [c]^ Dengsong Zhang*^, [a]^

[a] Y. Chen, Assoc. Prof. P. Wang, Dr. W. Qu, Dr. Y. Shen, Assoc. Prof. Y. Tang, Y. Ni, Dr. X. Hu, Dr. F. Wang, J. Zhang, D. Peng, X. Ding, Prof. D. Zhang

International Joint Laboratory of Catalytic Chemistry, State Key Laboratory of Advanced Special Steel, Innovation Institute of Carbon Neutrality,

Department of Chemistry, College of Sciences, Shanghai University, Shanghai 200444, People’s Republic of China.

E-mail: plwang@shu.edu.cn, dszhang@shu.edu.cn

[b] Dr. Y. Li

BASF Environmental Catalyst and Metal Solutions, Iselin, NJ 08830, United States.

E-mail: yuejin.li@basf-catalystsmetals.com

[c] E. Mariani, Prof. E. Cortes

Nanoinstitute Munich, Faculty of Physics, Ludwig-Maximilians-Universität (LMU), Munich 80539, Germany.

E-mail: Emiliano.Cortes@lmu.de

[d] Dr. W. Qu

Department of Chemistry, University of Toronto, 80 St. George Street, Toronto, ON M5S 3H6, Canada.

[e] Dr. Y. Shen

Institute for Chemical Reaction Design and Discovery (WPI-ICReDD), Hokkaido University, Sapporo 001-0021, Japan.

[f] Dr. M. Xie

Department of Chemical Engineering, University of Bath, Bath, BA27AY UK.

Table of Contents

**Experimental Procedures S4**

1. **Reagents and Materials Synthesis** S4
2. **Catalytic Performance Tests** S5
3. **Reaction Rate Research** S5
4. **Lifetime Research** S5
5. **Structure Characterization** S6
6. **Temperature Programmed Desorption and Surface Reaction Tests** S6
7. ***Quasi in situ* EPR spectra measurements** S7
8. ***In situ* DRIFTS spectra measurements** S7
9. **Computational method and model for density functional theory (DFT) calculations** S7
10. ***Ab initio* molecular dynamics (AIMD) simulations** S7

**Results and Discussion S8**

Figure S1. (a) NO_x_ conversion, (b) N_2_O concentration and N_2_ selectivity of the fresh and P-poisoned Cu-SSZ-13 catalysts in NH_3_-SCR. S8

Figure S2. (a) NO_x_ conversion, (b) N_2_O concentration and N_2_ selectivity of the Cu-SSZ-13_P and Sm_0.05_-Cu-SSZ-13_P catalysts in NH_3_-SCR. S9

Figure S3. (a) NO_x_ conversion, (b) N_2_O concentration and N_2_ selectivity of the Cu-SSZ-13_P and La_0.05_-Cu-SSZ-13_P catalysts in NH_3_-SCR. S10

Figure S4. (a) NO_x_ conversion, (b) N_2_O concentration and N_2_ selectivity of the Cu-SSZ-13_P and Pr_0.05_-Cu-SSZ-13_P catalysts in NH_3_-SCR. S11

Figure S5. (a) NO_x_ conversion, (b) N_2_O concentration and N_2_ selectivity of the Cu-SSZ-13_P and Nd_0.05_-Cu-SSZ-13_P catalysts in NH_3_-SCR. S12

Figure S6. NO_x_ conversion and N_2_ selectivity of Ce_0.0125_-Cu-SSZ-13, Ce_0.025_-Cu-SSZ-13, Ce-Cu-SSZ-13, Ce_0.1_-Cu-SSZ-13, and Cu-SSZ-13 catalysts in NH_3_-SCR. S13

Figure S7. NO_x_ conversion and N_2_ selectivity of Ce_0.0125_-Cu-SSZ-13_P, Ce_0.025_-Cu-SSZ-13_P, Ce-Cu-SSZ-13_P and Ce_0.1_-Cu-SSZ-13_P catalysts in NH_3_-SCR S14

Figure S8. (a) NO_x_ conversion, (b) N_2_O concentration and N_2_ selectivity of the (Cu-SSZ-13+zCeO_2_)_P (z=0, 10%, 20%, 30%, 40%, 50% in weight percent) catalysts in NH_3_-SCR. S15

Figure S9. NO_x_ conversion and N_2_ selectivity of the Cu-SSZ-13_P, Ce-Cu-SSZ-13_P and Cu-SSZ-13+40%CeO_2_)_P catalysts in NH_3_-SCR.. S16

Figure S10. The reaction rate histograms of Cu-SSZ-13 and Ce-Cu-SSZ-13_P in the low-temperature normalized on the Cu atomic content of Cu-SSZ-13. S17

Figure S11. NO_x_ conversion and N_2_ selectivity of the Cu-SSZ-13_P, Ce-Cu-SSZ-13_P, Ce-Cu-SSZ-13_0.8P, and Ce-Cu-SSZ-13_1.2P catalysts in NH_3_-SCR S18

Figure S12. NO_x_ conversion and N_2_ selectivity of the Cu-SSZ-13_P, Ce-Cu-SSZ-13_0.8P and Ce-Cu-SSZ-13_0.8P (AW/OT/HT/AE) catalysts in NH_3_-SCR. S19

Figure S13. EPR profiles of Ce-Cu-SSZ-13_0.8P, Ce-Cu-SSZ-13_0.8P(HT), Ce-Cu-SSZ-13_0.8P(AW) at room temperature (insert is the magnification of the EPR profiles within the range of 2500-3100G).. S20

Figure S14. UV-vis profiles of Ce-Cu-SSZ-13_0.8P, Ce-Cu-SSZ-13_0.8P(HT), Ce-Cu-SSZ-13_0.8P(AW)... S21

Figure S15. (a) NO_x_ conversion, (b) N_2_O concentration and N_2_ selectivity of the Cu-SSZ-13_yP (y=0.4, 0.5, 0.6, 0.7 mmol/g_cat_) (SAR=16) catalysts in NH_3_-SCR... S22

Figure S16. (a) NO_x_ conversion, (b) N_2_O concentration and N_2_ selectivity of the Cu-SSZ-13, Cu-SSZ-13_0.7P and Ce-Cu-SSZ-13_0.7P (SAR=16) catalysts in NH_3_-SCR.... S23

Figure S17. NO_x_ conversion and N_2_ selectivity of the Cu-SSZ-13_P, Ce-Cu-SSZ-13_P and Ce-Cu-SSZ-13_P (SSIE) catalysts in NH_3_-SCR..... S24

Figure S18. NO_x_ conversion and N_2_ selectivity of the (Ce_w_-Cu-SSZ-13)_P(SSIE) (w=0.5%, 1.0%, 1.5%, 2.0%, 2.23% in weight percent) catalysts in NH_3_-SCR...... S25

Figure S19. Plots of the NO_x_ conversion *versus* Ce loading over (Ce_w_-Cu-SSZ-13)_P(SSIE) (w=0.5%, 1.0%, 1.5%, 2.0%, 2.23% in weight percent) catalysts in NH_3_-SCR at 240 °C....... S26

Figure S20. Performance evaluation of Cu-SSZ-13_P in other environmental catalytic reactions (T=330 ℃) S27

Figure S21. NH_3_ conversion and N_2_ selectivity of Cu-SSZ-13_P and Ce-Cu-SSZ-13_P catalysts in NH_3_-SCO S28

Figure S22.  (a) NO*_x_* conversion and N_2_ selectivity, (b) CH_3_SH conversion and CO_2_ selectivity of Cu-SSZ-13_P and Ce-Cu-SSZ-13_P catalysts in SSCE of NO_x_ and CH_3_SH.. S29

Figure S23. The reaction products of (a) SO_2_ and (b) HCN formation over Cu-SSZ-13_P and Ce-Cu-SSZ-13_P catalysts in SSCE of NO_x_ and CH_3_SH.. S30

Figure S24. (a) n-B conversion and NO*_x_* conversion, (b) CO_2_ selectivity and N_2_ selectivity of Cu-SSZ-13_P and Ce-Cu-SSZ-13_P catalysts in SSCE of NO_x_ and n-B.. S31

Figure S25. The reaction products of (a) CO and (b) HCN formation over Cu-SSZ-13_P and Ce-Cu-SSZ-13_P catalysts in SSCE of NO_x_ and n-B. S32

Figure S26. XRD patterns for Cu-SSZ-13, Cu-SSZ-13_P and Ce-Cu-SSZ-13_P catalysts. S33

Figure S27. Relative crystallinity of the Cu-SSZ-13, Cu-SSZ-13_P and Ce-Cu-SSZ-13_P catalysts. S34

Figure S28. Crystallographic structure of Cu-SSZ-13 with 2.37 wt% Cu loading and its corresponding final Rietveld refinement results by using synchrotron X-ray scattering data. S35

Figure S29. H_2_-TPR profiles of Cu-SSZ-13, Cu-SSZ-13_P, Ce-Cu-SSZ-13 and Ce-Cu-SSZ-13_P catalysts. S36

Figure S30. Crystallographic structure of Ce-Cu-SSZ-13 with 1.88 wt% Cu, 2.41 wt% Ce loading and its corresponding final Rietveld refinement results by using synchrotron X-ray scattering data. S37

Figure S31. (a) HR-TEM image and line scan results of the Cu-SSZ-13_P catalyst. (b-f) EDS mapping results of Cu, Al, O and Si elements distribution over the Cu-SSZ-13_P catalyst (blue represents Cu, orange represents Al, purple represents O, yellow represents Si, and red represents P). S38

Figure S32. HR-TEM image of the Cu-SSZ-13_P catalyst S39

Figure S33. HR-TEM image of the Ce-Cu-SSZ-13_P catalyst. S40

Figure S34. (a) AC-HAADF-STEM pictures and (b) HAADF-STEM-EDS mapping results of the Cu-SSZ-13_P catalyst. S41

Figure S35. Ce 3d XPS spectra of Ce-Cu-SSZ-13 and Ce-Cu-SSZ-13_P catalysts S42

Figure S36. Cu 2p XPS spectra of the Cu-SSZ-13 catalyst S43

Figure S37. FT-EXAFS spectra of CeO_2_. S44

Figure S38. Local structures and energy profiles of PO_4_^3-^ adsorption on Cu^2+^ sites, Al^3+^ sites and Ce^3+^ sites. S45

Figure S39. Global structures and energy profiles of PO_4_^3-^ adsorption on (1-4) Cu^2+^, (5-7) Al^3+^ and (8-10) Ce^3+^ sites. S46

Figure S40. Calculated time-dependent distance between the PO_4_^3-^ and Ce sites over the structure of Ce_2_-OPO_3_ in AIMD simulation S47

Figure S41. RDFs for Cu-O (Cu_1_-OPO_3_) and Cu-O (Ce_2_-OPO_3_) collected from MD simulations S48

Figure S42. RDFs for Ce-O (Ce_2_-OPO_3_) collected from MD simulations. S49

Figure S43. NH_3_-TPD-MS profiles of Cu-SSZ-13_P and Ce-Cu-SSZ-13_P catalysts S50

Figure S44. NO+O_2_-TPD-MS profiles of Cu-SSZ-13_P and Ce-Cu-SSZ-13_P catalysts S51

Figure S45. *In situ* DRIFTs of the transient reactions between pre-adsorbed NH_3_ and NO + O_2_ as a function of time over the Cu-SSZ-13_P catalyst S52

Figure S46. *In situ* DRIFTs of the transient reactions between pre-adsorbed NO + O_2_ and NH_3_ as a function of time over the (a) Ce-Cu-SSZ-13_P and (b) Cu-SSZ-13_P catalysts S53

Table S1. Comparison between Ce-Cu-SSZ-13_P with other reported P-tolerant NH_3_-SCR catalysts S54

Table S2. Element contents in different samples from ICP-OES. S55

Table S3. Refine atomic position, occupancies, and thermal displacement parameters for the XRD pattern of the Cu-SSZ-13 catalyst. Cu_1_ represent [ZCu^2+^OH]^+^ occupying at 8MRs, Cu_2_ represent Z_2_Cu^2+^ occupying at 6MRs. S56

Table S4. Refine atomic position, occupancies, and thermal displacement parameters for the XRD pattern the Cu-SSZ-13_P catalyst. Cu_1_ represent [ZCu^2+^OH]^+^ occupying at 8MRs, Cu_2_ represent Z_2_Cu^2+^ occupying at 6MRs S57

Table S5. Refine atomic position, occupancies, and thermal displacement parameters for the XRD pattern the Ce-Cu-SSZ-13 catalyst. Cu_1_ represent [ZCu^2+^OH]^+^ occupying at 8MRs, Cu_2_ represent Z_2_Cu^2+^ occupying at 6MRs, Ce_1_ represent [Z_1_Ce^3+^(OH)_2_]^+^ occupying at 8MRs. S58

Table S6. Refine atomic position, occupancies, and thermal displacement parameters for the XRD pattern the Ce-Cu-SSZ-13_P catalyst. Cu_1_ represent [ZCu^2+^OH]^+^ occupying at 8MRs, Cu_2_ represent Z_2_Cu^2+^ occupying at 6MRs, Ce_1_ represent [Z_1_Ce^3+^(OH)_2_]^+^ occupying at 8MRs. S59

Table S7. EXAFS fitting parameters at the Cu K-edge for Cu-SSZ-13_P and Ce-Cu-SSZ-13_P catalysts. S60

Table S8. Quantitative analysis result of NH_3_-TPD-MS profiles. S61

Movie S1. AIMD simulation for the PO_4_^3-^ inter-cage diffusion in Ce-Cu-SSZ-13 (MP4). S62

Movie S2. AIMD simulation for the PO_4_^3-^ inter-cage diffusion in Cu-SSZ-13 (MP4). S63

**References S64**

**Author Contributions S64**

Experimental Procedures

**1. Reagents and Materials Synthesis**

Cerium nitrate (Ce(NO_3_)_3_·6H_2_O), Diammonium hydrogen phosphate ((NH_4_)_2_HPO_4_) and Oxalic acid dihydrate (H_2_C_2_O_4_·2H_2_O) were purchased from Sinopharm Group Co. Ltd. Praseodymium nitrate (Pr(NO_3_)_3_·6H_2_O), Neodymium nitrate (Nd(NO_3_)_3_·6H_2_O), Lanthanum nitrate (La(NO_3_)_3_·6H_2_O), Samarium nitrate (Sm(NO_3_)_3_·6H_2_O) were purchased from Shanghai Diyang Chemical Co., LTD. Ceric dioxide (CeO_2_) and Ammonium chloride (NH_4_Cl) were purchased from Shanghai Macklin Biochemical Co., Ltd. Cu-SSZ-13 is the commercial catalyst for mobile source NO_x_ removal, supplied by BASF. All the reagents were used without further purification.

Ce with different content was introduced into Cu-SSZ-13 (SAR=27) by one-step ion exchange. The Cu-SSZ-13 sample where the Ce ions exchanged was performed was written as Ce_x_-Cu-SSZ-13 (x is the concentration of Ce(NO_3_)_3_ solution, its value is 0.0125 M, 0.025 M, 0.05 M or 0.1 M). Phosphorus was introduced to Cu-SSZ-13 or Ce_x_-Cu-SSZ-13 by traditional impregnation method, and (NH_4_)_2_HPO_4_ was used as the precursor of phosphorus. Based on the theoretical content of phosphorus, Cu-SSZ-13 with different phosphorus loading were denoted as Cu-SSZ-13_yP (y = 0.05, 0.1, 0.2, 0.3, and 0.4 mmol/g_cat_). The catalyst study for anti-phosphorus poisoning in this work was based on a theoretical phosphorus loading of 0.4 mmol/g_cat_ and a concentration of 0.05 M Ce(NO_3_)_3_ solution. Therefore, Cu-SSZ-13_0.4P, Ce_0.05_-Cu-SSZ-13 and Ce_0.05_-Cu-SSZ-13_0.4P were written as Cu-SSZ-13_P, Ce-Cu-SSZ-13 and Ce-Cu-SSZ-13_P. After hydrothermal aging treatment at 650 °C for 50 h in 10% H_2_O in air, the catalysts were recorded as Ce-Cu-SSZ-13(A) and Ce-Cu-SSZ-13_P(A), respectively.

Cu-SSZ-13_yP (y = 0.05, 0.1, 0.2, 0.3, and 0.4 mmol/g_cat_) catalysts were prepared by the traditional impregnation method. First, solution A (0.1 M (NH_4_)_2_HPO_4_ solution) was configured using a 100 mL volumetric flask. Second, 1 g Cu-SSZ-13 was dispersed in 39.5 mL/39 mL/38 mL/37 mL/36 mL deionized water at room temperature for obtaining dispersion liquid B. Then 0.05 mL/1 mL/2 mL/3 mL/4 mL of solution A was added dropwise to the dispersion liquid B, and then kept stirring for 2 h to ensure optimal mixing. Subsequently, the obtained liquid was rotary evaporated at 60 °C, dried in a blast oven at 80 °C and calcined in a muffle furnace at 550 °C in air for 4 h to obtain Cu-SSZ-13_yP.

Ce_x_-Cu-SSZ-13 (x is the concentration of Ce(NO_3_)_3_ solution, its value is 0.0125 M, 0.025 M, 0.05 M or 0.1 M) catalysts were synthesis by one-step ion exchange. Firstly, add 1 g Cu-SSZ-13 and 0.5428 g/1.0856 g/2.1711 g/4.3422 g Ce(NO_3_)_3_·6H_2_O in a 200 mL bottle containing 100 mL of distilled water. The mixtures were stirred at 80 °C for 2 h, then filtrated and washed with deionized water until PH=7, dried and calcined at 550°C for 4 h in air to obtain Ce_x_-Cu-SSZ-13.

To verify the anti-phosphorus properties of different types of secondary ions, we synthesized the fresh and poisoned Cu-SSZ-13 samples with different types of secondary ions modification, which was denoted as A_0.05_-Cu-SSZ-13 and A_0.05_-Cu-SSZ-13_P (A=Pr, Nd, La, Sm). It is worth noting that the concentration of other ions exchange solution was 0.05M and they have the same preparation method as Ce_x_-Cu-SSZ-13 and Ce_x_-Cu-SSZ-13_P.

To verify the critical role of Ce sites among the Ce-Cu-SSZ-13 catalyst, we prepare the catalysts by physical mixing Cu-SSZ-13 with different amount of CeO_2_ (10%, 20%, 30%, 40%, 50% in weight percent), which was denoted as (Cu-SSZ-13 +zCeO₂)_P (z=10%, 20%, 30%, 40%, 50%) (P=0.4 mmol/g_cat_). In details, weigh 1 g of Cu-SSZ-13 and the corresponding amount of CeO₂ into a mortar at room temperature, then grind thoroughly to yield the Cu-SSZ-13 +zCeO₂ catalyst. Preparation of (Cu-SSZ-13 + zCeO₂)P catalyst via the impregnation method.

To predict the deactivation threshold of catalysts, we prepared Ce-Cu-SSZ-13 with higher phosphorus content (0.8 mmol/g_cat_ and 1.2 mmol/g_cat_) by impregnation method that same with the case of Ce-Cu-SSZ-13_P, which was denoted as Ce-Cu-SSZ-13___0.8P and Ce-Cu-SSZ-13___1.2P.

To clarify long-term viability of Ce-Cu-SSZ-13, regeneration of the Ce-Cu-SSZ-13_0.8P catalyst was conducted through a process involving oxidation treatment (OT, a 2 h pre-treatment in 10% O_2_/N_2_), hydrothermal treatment (HT, 50 h of hydrothermal aging at 650°C), mild acid washing (AW, a 1 h wash with 0.1 M H_2_C_2_O_4_(aq)), and ammonium salt exchange (AE, a 2 h ion exchange with 1 M NH_4_Cl solution at 80°C).

To evaluate the universality of Ce modification strategy on Cu-SSZ-13 zeolite with different silicon-to-aluminium ratio (SAR), phosphorus was incorporated into Cu-SSZ-13 with a lower silicon-aluminium ratio (SAR=16) via the same immersion method with Cu-SSZ-13_P (SAR=27). The resulting samples were denoted as Cu-SSZ-13_yP (y = 0.5, 0.6, 0.7 mmol/g_cat_) (SAR=16).

To facilitate large-scale implementation, we evaluated the feasibility of solid-state ion exchange method to synthesize the Ce-Cu-SSZ-13 catalyst. The catalysts prepared by solid-state ion exchange (SSIE) were denoted as (Ce_w_-Cu-SSZ-13)_P(SSIE) (w=0.5%, 1.0%, 1.5%, 2.0%, 2.23% in weight percent) (P=0.4 mmol/g_cat_). In details, Weigh the required mass of Ce(NO₃)₃·6H₂O into a crucible. Add sufficient deionized water to dissolve it completely. Under stirring, add the Cu-SSZ-13 catalyst to achieve thorough wetting, and then dry the mixture overnight at room temperature. After calcination in a muffle furnace, phosphorus is introduced into the catalyst via impregnation.

To validate the universality of this resistance strategy when applied to molecular sieves with different silicon-aluminium ratios, we synthesised Ce-Cu-SSZ-13_0.7P (SAR=16) prepared from Cu-SSZ-13 (SAR=16) via solid-state ion exchange (Ce content equivalent to that of the Ce-Cu-SSZ-13 (SAR=27) sample.

**2. Catalytic Performance Tests**

The NH_3_-SCR / NH_3_-SCO / SSCE of NO_x_ and CH_3_SH / SSCE of NO_x_ and n-B activity tests were performed using 0.15 g / 0.15 g / 0.3 g / 0.3 g of catalysts (40-60 mesh) in a fixed-bed reactor. The gas mixture compositions were: 500 ppm NO, 500 ppm NH_3_, 10% O_2_, 5% H_2_O, 5% CO_2_, N_2_ as the balance, and WHSV = 200, 000 mL·g^-1^·h^-1^ for NH_3_-SCR. 500 ppm NH_3_, 10 % O_2_, 5% H_2_O, N_2_ as balance, and WHSV = 200, 000 mL·g^-1^·h^-1^ for NH_3_-SCO. 500 ppm NO, 500 ppm NH_3_, 100 ppm CH_3_SH, 5 % O_2_, N_2_ as balance, and GHSV = 25,000 h^−1^ for SSCE of NO_x_ and CH_3_SH. 100 ppm NO, 100 ppm NH_3_, 200 ppm n-B, 5 % O_2_, N_2_ as balance, and GHSV = 50,000 h^−1^ for SSCE of NO_x_ and n-B. The WHSV/GHSV was obtained by the following formula:

$WHSV=\frac{q_{v}}{m}$ (1)

$GHSV=\frac{q_{v}}{\Pi\cdot h\cdot r^{2}}$ (2)

q_v_ corresponds to the total flow rate; m denotes the weight of the catalysts in the reactor; h denotes the height of the catalysts in the reactor; r represents the radius of the reaction tube.

The concentrations of the tail gas at the outlet of the reactor were measured by an FTIR spectrometer (Thermo Fisher). The catalytic activity was recorded after the reaction system reached a steady state. The conversion rate and selectivity were calculated by the following equation:

$\mathrm{NO}_{x}\mathrm{conversion}\left( \% \right)=\frac{\left[ \mathrm{NO}_{x} \right]_{\mathrm{in}}-\left[ \mathrm{NO}_{x} \right]_{\mathrm{out}}}{\left[ \mathrm{NO}_{x} \right]_{\mathrm{in}}}$×100% (3)

$N_{2}\mathrm{selectivity}\left( \% \right)=(1-\frac{2\left[ N_{2}O \right]}{\left[ \mathrm{NO}_{x} \right]_{\mathrm{in}}+\left[ \mathrm{NH}_{3} \right]_{\mathrm{in}}-\left[ \mathrm{NO}_{x} \right]_{\mathrm{out}}-\left[ \mathrm{NH}_{3} \right]_{\mathrm{out}}})$×100% (4)

$NH_{3} conversion (\%)=\left( \frac{\left[ \mathrm{NH}_{3_{\mathrm{in}}} \right]-\left[ \mathrm{NH}_{3_{\mathrm{out}}} \right]}{\left[ \mathrm{NH}_{3_{\mathrm{in}}} \right]} \right)\times100\%$ (5)

$\text{CH}_{\text{3}}\text{SH conversion }\left( \text{\%} \right)\text{ = }\frac{{\text{[}\text{CH}_{\text{3}}\text{SH]}}_{\text{in}}\text{-}{\text{[}\text{CH}_{\text{3}}\text{SH]}}_{\text{out}}}{{\text{[}\text{CH}_{\text{3}}\text{SH]}}_{\text{in}}}\times100\%$ (6)

$\text{CO}_{\text{2}}\text{ yield }\left( \text{\%} \right)\text{ = }\frac{{\text{[}\text{CO}_{\text{2}}\text{]}}_{\text{out}}}{{\text{[}\text{CH}_{\text{3}}\text{SH]}}_{\text{in}}\text{-}{\text{[}\text{CH}_{\text{3}}\text{SH]}}_{\text{out}}}\times100\%$ (7)

$n-B conversion (\%)=\left( \frac{\left[ n-butylamine_{\mathrm{in}} \right]-\left[ n-butylamine_{\mathrm{out}} \right]}{\left[ n-butylamine_{\mathrm{in}} \right]} \right)\times100\%$ (8)

Where NO_x_ denotes the total concentration of NO and NO_2_. [NO_x_]_in_, [NH_3_]_in_, [NO_x_]_out_, [N_2_O]_out_, [NH_3_]_in_, [NH_3_]_out_, [CH_3_SH]_in_, [CH_3_SH]_out_, [n-B]_in_, [n-B]_out_, [CO_2_]_out_ denote the corresponding inlet and outlet gas concentrations respectively.

**3. Reaction Rate Research**

The reaction rate was calculated by the equation:

$R=\frac{F_{\mathrm{NO}_{x}}\times X_{\mathrm{NO}_{x}}}{22.4W\times X_{\mathrm{Cu}}}$ (9)

Where F_NOx_ is the NO_x_ concentration of inlet NO_x_ molecules (L^.^min^-1^), X_NOx_ is the NO_x_ conversion, W is the weight of the catalysts (g), X_Cu_ is the atomic percentage of Cu obtained from ICP-OES results.

**4. Lifetime Research**

*Biodiesel Handling and Use Guide* *(Fifth Edition, National Renewable Energy Laboratory, United States, 2016, DOE/GO-102016-4875.)* stipulate that phosphorus content involved in biodiesel (B100, 100% biodiesel) is limited to 10 ppm (equivalent to 10 mg_P_/kg_fuel_) maximum. Under optimal conditions, heavy-duty diesel engines have a standard fuel consumption rate of 190~250 g/kWh (*SAE paper 2009-01-1471, 2009*). Based on a typical average power output of 118~346 kW, the required active component mass in an SCR catalyst is 4.875 kg (*Limits and measurement methods for emissions from diesel fuelled heavy-duty vehicles (China VI)*). To simplify the calculation process, a set of baseline values was selected: a fuel consumption rate (Fr) of 200 g_fuel_/kWh, an engine power (Ep) of 100 kW, and a catalyst weight (M) of 5 kg. Currently, countries such as China typically blend 5% biodiesel into diesel vehicles during actual operation, therefore the phosphorus content (C) in the B5 biodiesel is 0.5 mg_P_/kg_fuel_. The deposition quantity of phosphorus (D) required for complete deactivation over the Cu-SSZ-13 and Ce-Cu-SSZ-13 catalysts are 0.4 and 0.8 mmol_p_/g_cat_ (equivalent to 12 and 24 mg_P_/g_cat_), respectively. The catalyst lifetime was calculated by the following equations:

$D_{r}=\frac{F_{r}\times E_{P}\times C}{M}$ (10)

$L=\frac{D}{D_{r}}$ (11)

Where Fr is the fuel consumption rate (200 g_fuel_/kWh), Ep is the engine power (100 kW), C is phosphorus content in B5 biodiesel (5% biodiesel) fuel (0.5 mg_P_/kg_fuel_), M is the weight of the catalysts (5 kg), D is the deposition quantity of phosphorus (mg_p_/g_cat_), Dr is the deposition rate of phosphorus (mg_p_/(g_cat_·h)), L is the catalyst lifetime (h).

**5. Structure Characterization**

The structure and morphology of the catalyst were observed by X-ray diffraction (XRD) spectra, high-resolution transmission electron microscopic (HR-TEM), aberration-corrected high angle annular dark field-scanning transmission electron microscopy (AC-HAADF-STEM), ^31^P solid-state nuclear magnetic resonance (SSNMR) and ^27^Al SSNMR spectra, Raman spectra, UV-visible diffuse reflectance spectroscopy (UV-Vis-DRS), electron paramagnetic resonance (EPR), X-ray photoelectron spectroscopy (XPS), X-ray absorption fine structure (XAFS), electrochemical impedance spectroscopy (EIS), inductively coupled plasma-optical emission spectrometer (ICP-OES).

XRD spectra for catalysts recorded in the range of 5° to 40° were measured on a Rigaku D/MAS-RB X-ray diffractometer with Cu Kα (40 kV, 40 mA) radiation. Synchrotron XRD spectra were collected at BL17B beamline of Shanghai Synchrotron Radiation Facility. The X-ray energy was adjusted to 18 keV, to activate the anomalous dispersion effect. Powder samples were filled into glass capillaries with an outer diameter of 0.5 mm. And each sample was exposed to X-ray radiation for 9 s or 50 s to collect the XRD patterns. Rietveld refinement was performed by using Topas. HR-TEM were conducted on JEOL JEM-2010F. AC-HAADF-STEM pictures were collected by a JEOL Grand Arm at 300 kV. ^31^P SSNMR experiments were performed on Agilent 600 DD2 spectrometre (Agilent, USA, magnetic field strength 14.1T) at a resonance frequency of 242.76 MHz. ^31^P SSNMR spectra were recorded with spinning rate of 8KHz with a 4mm probe at room temperature. The P signal of (CH_3_O)_3_PO was used as the reference of ^31^P chemical shift. ^27^Al SSNMR experiment was carried out an Agilent 600 DD2 spectrometer with a Larmor frequency of 156.25 MHz for Al and proton decoupling (TPPM) during acquisition. The powder samples were placed in a pencil-type zirconia rotor of 4.0 mm. The spectra were obtained at a spinning speed of 8 kHz with a recycle delay of 1s, an approximately 90-degree pulse (3.6us) and 128 scans. The Al signal of AlCl_3_ at 0.9 ppm was used as the reference of ^27^Al chemical shift. Raman spectra were recorded on the LabRAM HR Evolution (Horiba). Raman spectra were acquired in the range of 100-2000 cm^-1^ using a laser with a wavelength of 532 nm. The UV-Vis-DRS was collected using an Agilent Cary 5000 UV-Vis-NIR spectrometer equipped with a DRS accessory to allow collection in the diffuse reflectance mode, against a pure white reference standard. Spectra were collected between 200 and 800 nm with a data interval of 1 nm and at a rate of 200 nm/min. EPR experiments were carried out on a Bruker EMX plus spectrometer. Powder samples were contained in 4 mm OD quartz tubes. During spectral acquisition, microwave power was 1 mW, and the frequency was 100 kHz. The field was swept 1500 G in 84 s and modulated at 100 kHz with a 3 G amplitude. The surface atomic valence of the catalysts was studied by XPS system (PHI-5300) with Mg Kα radiation. The binding energies of P, Cu, O and Ce were corrected for the containment carbon peak (C 1s = 284.8 eV), and the peak fitting was performed through casa XPS software. Extended X-ray absorption fine structure (EXAFS) spectra of Cu K-edge and Ce L_3_-edge were conducted at room temperature using a Debye−Scherrer camera installed at the BL17B (λ = 0.7081(1) Å) beamline at the Shanghai Synchrotron Radiation Facility. Demeter package are used to analyze the obtained EXAFS. Wavelet transformation (WT) was carried out by a HAMA package. To fabricate the working electrode, 5 mg of catalyst was dispersed via sonication in a blend of ethanol and deionized water to form a homogeneous catalyst ink. The resulting ink was then drop-cast onto a dry carbon paper (CP) substrate with an area of 1 cm². Electrochemical impedance spectroscopy (EIS) was acquired at open-circuit potential under 25°C conditions, covering a frequency range from 0.01 Hz to 100 kHz without internal resistance correction. ICP-OES was conducted on Agilent 5110.

**6. Temperature Programmed Desorption and Surface Reaction Tests**

The hydrogen temperature programmed reduction (H_2_-TPR) was conducted on a Micromeritics AutoChem 2920 II auto-adsorption apparatus with a thermal conductivity detector (TCD). Prior to the reduction process, 80 mg of each catalyst was treated under 2 vol.% O_2_/He atmosphere with a flow rate of 20 mL/min at 500 °C for 1 h, then cooled to room temperature under He. In H_2_-TPR runs, the catalysts were exposed to 10 vol.% H_2_/Ar and then the reactor temperature was raised from room temperature to 800 °C with a rate of 10 °C/min. The Micromeritics AutoChem 2920 II auto-adsorption apparatus was employed for NH_3_ temperature programmed desorption coupled with a mass spectrometer (NH_3_-TPD-MS) experiments with a TCD to monitor the NH_3_. Prior to the NH_3_-TPD-MS experiments, 80 mg catalysts were outgassed under 2 vol.% O_2_/He protection (30 mL/min) at 500 °C for 1 h and then cooled to 50 °C. Samples were exposed to 10% NH_3_/He for one hour at 50 °C, the physical adsorption of ammonia was removed by He puring for 10 min at the same temperature. Finally, the temperature was raised to 800 °C with a ramping rate of 10 °C/min. The NO+O_2_ temperature programmed desorption coupled with a mass spectrometer (NO+O_2_-TPD-MS) was carried out on a Tianjin XQ TP-5080 auto-adsorption apparatus with a TCD to monitor the NO_x_ species. Similarly, with the NO+O_2_-TPD-MS experiment, 80 mg catalysts were outgassed under 10 vol.%O_2_/He protection (30 mL/min) at 300 °C for 30 min and then cooled to 50 °C. Samples were exposed to the samples were exposed to a flow of NO+O_2_ at the same temperature for 1 h, the physical adsorption of NO was removed by He purging for 10 min at the same temperature. Finally, the temperature was raised to 800 °C with a ramping rate of 10 °C/min.

**7. *Quasi in situ* EPR spectra measurements**

*Quasi in situ* EPR spectra recorded in the absorption of NO+O_2_ or NH_3_. The experiments were carried out on a Chinainstru&Quantumtech EPR200-Plus spectrometer. Powder samples were contained in 4 mm OD quartz tubes. During spectral acquisition, microwave power was 1 mW, and the frequency was 100 kHz. The field was swept 1500 G in 84 s and modulated at 100 kHz with a 3 G amplitude.

**8. *In situ* DRIFTS spectra measurements**

*In situ* diffuse reflectance infrared Fourier transform spectroscopy (*In situ* DRIFTS) spectroscopic experiments were carried out on a Nicolet 6700 spectrometer equipped with a Harrick Scientific DRIFTS cell and a liquid nitrogen cooled mercury cadmium telluride (MCT) detector. The DRIFTS spectra were collected in the range of 600-4000 cm^-1^ in Absorbance units at 4 cm^-1^ resolution. Prior to each measurement, the catalysts were pretreated in 10 vol.%O_2_/N_2_ at 500 °C for 1 h and then cooled to the reaction temperature (180 °C) in the same atmosphere for background acquisition. For transient reactions between NH_3_ and NO + O_2_, NH_3_ (or NO + O_2_) with a total flow rate of 30 mL/min was firstly introduced into the cell at 180 °C for 1 h. Next, pure N_2_ with a flow rate of 30 mL/min was applied for 10 min to purge catalysts at 180 °C. Then, NO + O_2_ (or NH_3_) with a total flow rate of 30 mL/min was introduced into the cell to initiate titration reactions at 180 °C under continuous IR scanning. Furthermore, as for the adsorption-desorption of NH_3_ or NO + O_2_ studies, after being pre-treated at 500 °C and obtaining the background spectra of different temperatures, the catalysts were exposed to a flow of 500 ppm of NH_3_ or NO + O_2_ at 30 °C for 1 h. The desorption process then went on under a flow of N_2_ and was recorded at corresponding temperatures. Testing conditions: 500 ppm NO, 500 ppm NH_3_, 10 vol.% O_2_, and N_2_ as the balance gas.

**9. Computational method and model for density functional theory (DFT) calculations**

The periodic SSZ-13 zeolite model was prepared using an orthorhombic unit cell (modified from the standard hexagonal primary cell of CHA framework) with the size parameters of 13.6750 × 23.6858 × 14.7670 Å^3^. To mimic the experimental Si/Al ratio of 27 for SSZ-13 zeolites used in this work, the same Si/Al ratio of 7 was set up by replacing 3 Si atoms on the SSZ-13 framework structure with 3 Al, and compensating the charges with additional 3 H atoms on the O atoms, resulting in the model SSZ-13 zeolite of H_3_Al_3_Si_69_O_144_.The Vienna ab initio Simulation package (VASP)^[1]^ was used to calculate the energy of the adsorption systems under the framework of density functional theory. The generalized gradient approximation (GGA) with the Perdew-Burke-Ernzerhof (PBE)^[2]^ function was used for the evaluation of the exchange-correlation energy. The plane wave basis set was set to an energy Cutoff of 500 eV to provide accurate self-consistent charge density. For geometry optimizations, the force and energy convergences were 0.05 eV/Å and 10^-5^ eV, respectively. The DFT + U was applied to address the on-site Coulomb interaction with the U-J parameters of 6 eV for Cu 3d and 5 eV for Ce 4f.^[3]^ To account for van der Waals interaction, the DFT-D3 method of Grimme was utilized.^[4]^ The Brillouin zone was sampled with a single gamma grid for geometry optimizations. The adsorption energy of PO_4_^3-^ (E) was calculated by the following equation:

$$E=E_{{{PO}_{4}}^{3-}/Cat}{- E}_{Cat}- E_{{{PO}_{4}}^{3-}}$$

where $E_{{{PO}_{4}}^{3-}/Cat}$ is the total energy of Cu-SSZ-13 or Ce-Cu-SSZ-13 after adsorption of PO_4_^3-^, $E_{Cat}$ is the energy of Cu-SSZ-13 or Ce-Cu-SSZ-13, $E_{{{PO}_{4}}^{3-}}$ is the energy of the single PO_4_^3-^ molecule.

**10.** ***Ab initio* molecular dynamics (AIMD) simulations**

The *ab initio* molecular dynamics (AIMD) simulations were performed to directly observe the intercage diffusion of PO_4_^3-^ in the Cu-SSZ-13_P and Ce-Cu-SSZ-13_P catalysts. The time step was chosen to be 1fs, and Newton’s motion equation is integrated using the Verletalgorithm implemented in VASP. The simulations were carried out for 14 ps. The Brillouin zone was sampled at the gamma point. The temperature was controlled to 100°C using a Nose−Hoover thermostat.

Results and Discussion


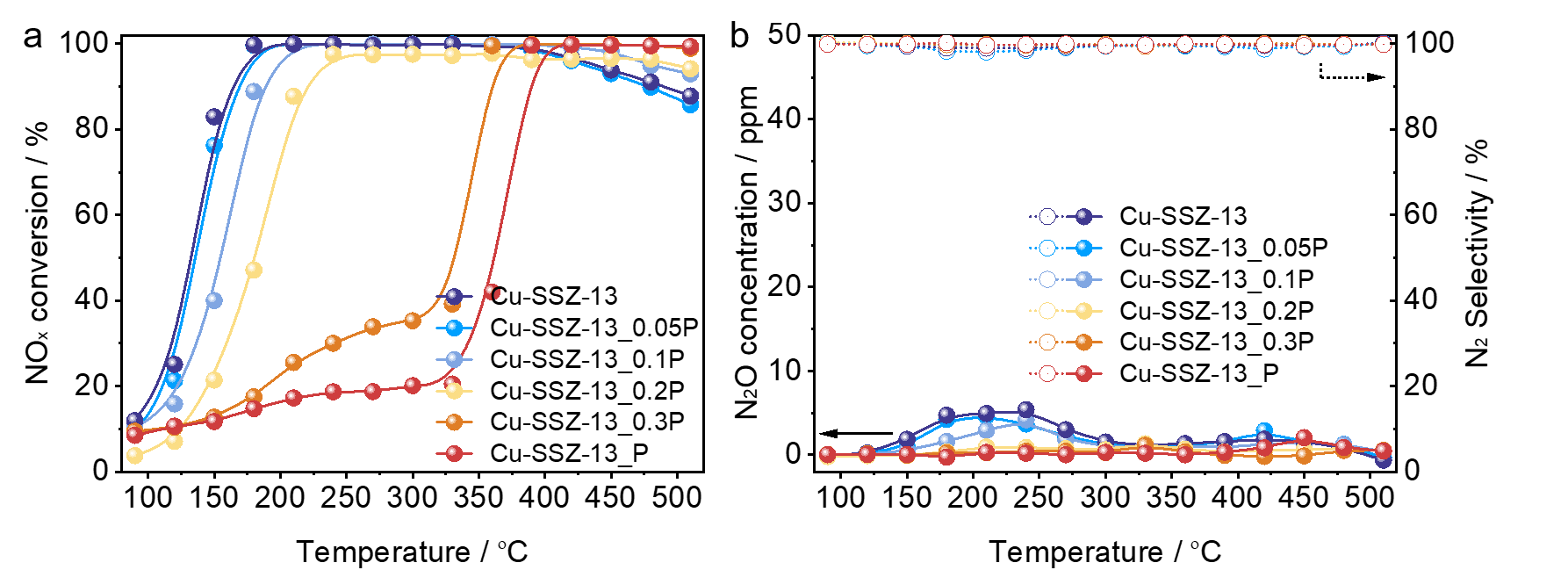


**Figure S1.** (a) NO_x_ conversion, (b) N_2_O concentration and N_2_ selectivity of the fresh and P-poisoned Cu-SSZ-13 catalysts in NH_3_-SCR. Reaction conditions: 500 ppm NO, 500 ppm NH_3_ and 10% O_2_, 5% H_2_O, 5% CO_2_ with N_2_ as the balance, WHSV = 200, 000 mL·g^-1^·h^-1^.

The NO_x_ conversion of Cu-SSZ-13 decreases as the phosphorus loading increases. Furthermore, N_2_O concentration of Cu-SSZ-13 gradually decreases as phosphorus content increases, indicating that phosphorus enhances N_2_ selectivity by inhibiting the non-selective oxidation of NH_3_. In addition, in order to simulate the actual cumulative phosphorus poisoning amount in the exhaust gas of biodiesel vehicles, we select Cu-SSZ-13 catalyst with 0.4 mmol/g_cat_ phosphorus loading as the research object.


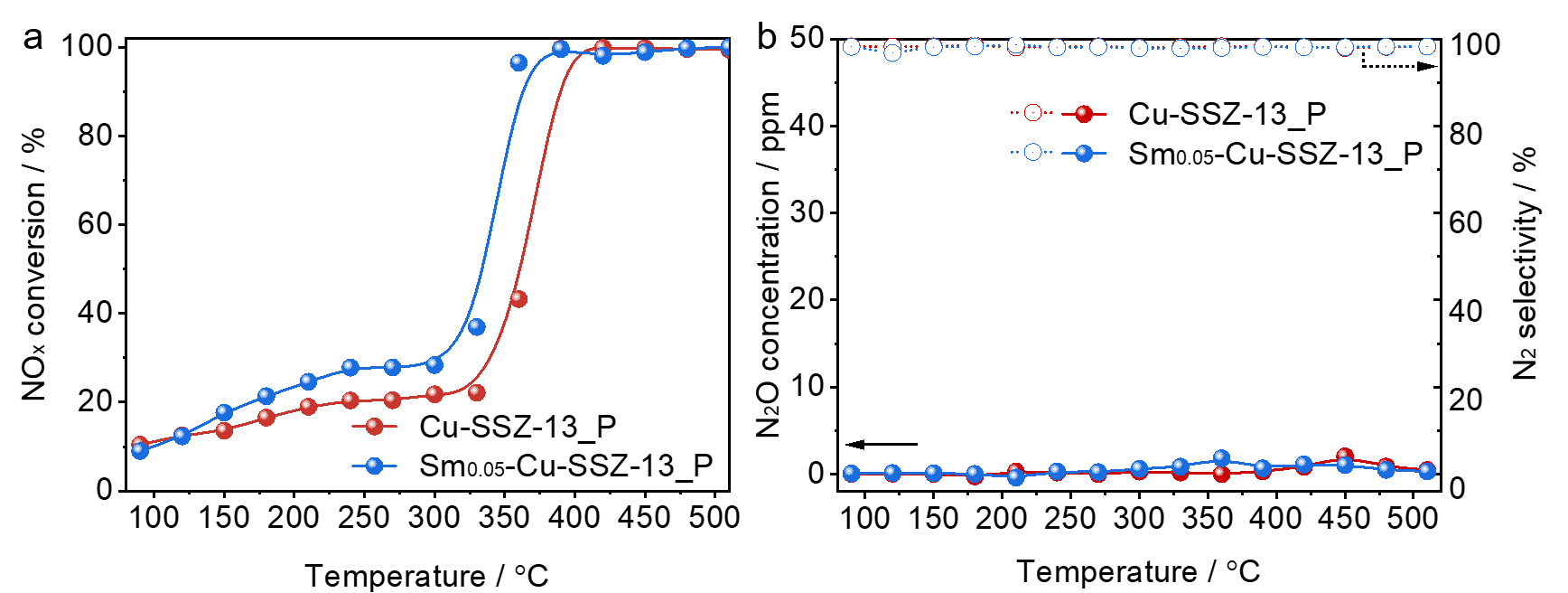


**Figure S2.** (a) NO_x_ conversion, (b) N_2_O concentration and N_2_ selectivity of the Cu-SSZ-13_P and Sm_0.05_-Cu-SSZ-13_P catalysts in NH_3_-SCR. Reaction conditions: 500 ppm NO, 500 ppm NH_3_ and 10% O_2_, 5% H_2_O, 5% CO_2_ with N_2_ as the balance, WHSV = 200, 000 mL·g^-1^·h^-1^.


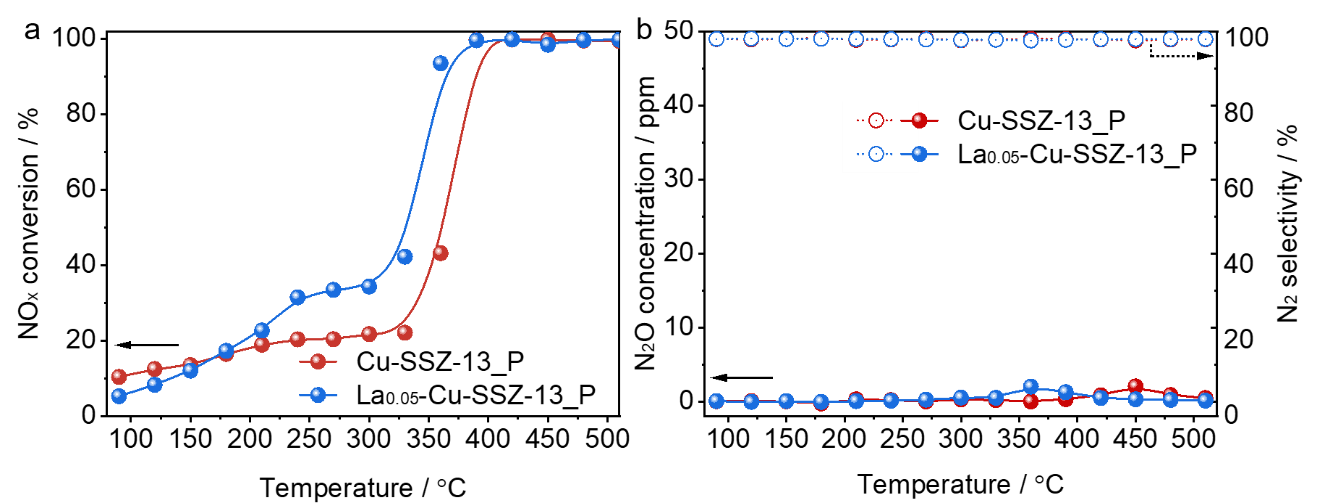


**Figure S3.** (a) NO_x_ conversion, (b) N_2_O concentration and N_2_ selectivity of the Cu-SSZ-13_P and La_0.05_-Cu-SSZ-13_P catalysts in NH_3_-SCR. Reaction conditions: 500 ppm NO, 500 ppm NH_3_ and 10% O_2_, 5% H_2_O, 5% CO_2_ with N_2_ as the balance, WHSV = 200, 000 mL·g^-1^·h^-1^


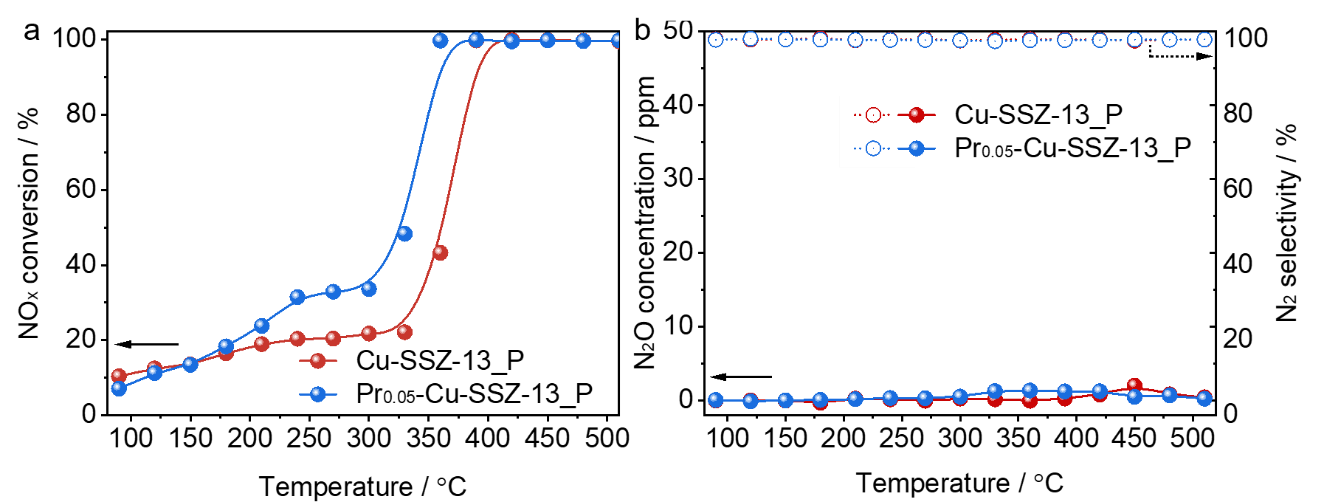


**Figure S4.** (a) NO_x_ conversion, (b) N_2_O concentration and N_2_ selectivity of the Cu-SSZ-13_P and Pr_0.05_-Cu-SSZ-13_P catalysts in NH_3_-SCR. Reaction conditions: 500 ppm NO, 500 ppm NH_3_ and 10% O_2_, 5% H_2_O, 5% CO_2_ with N_2_ as the balance, WHSV = 200, 000 mL·g^-1^·h^-1^.


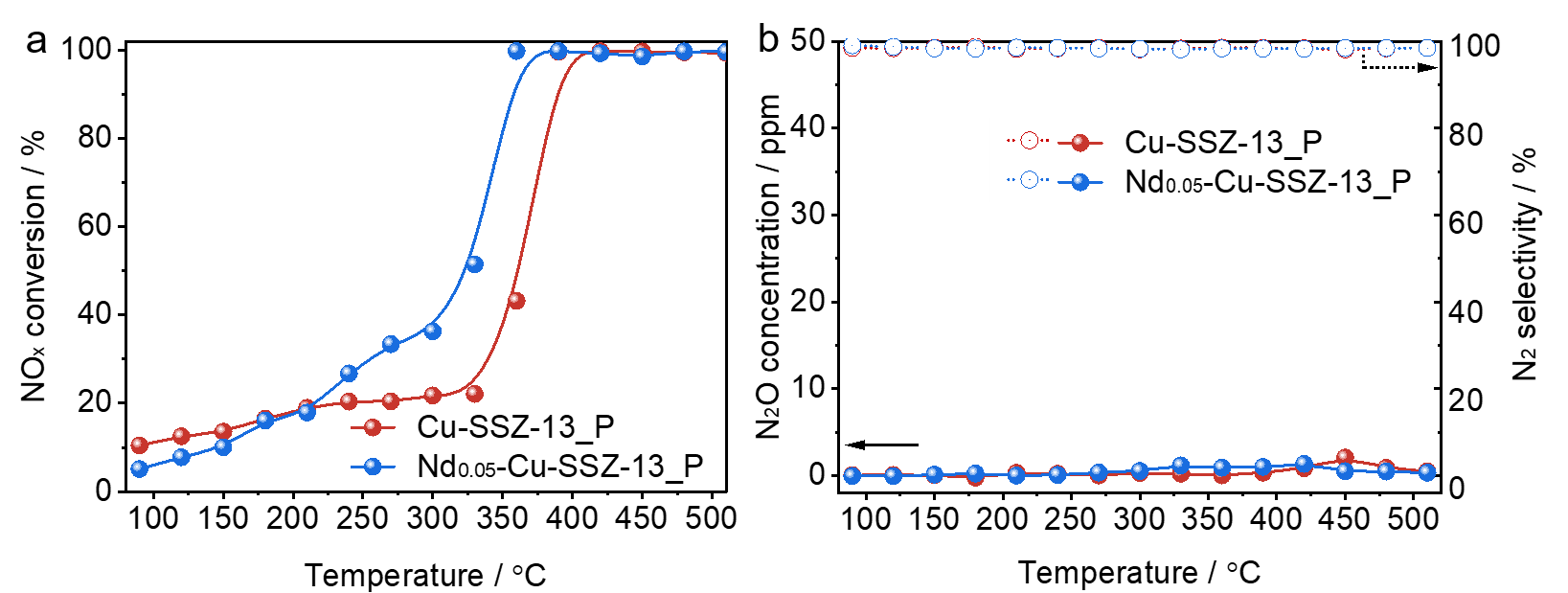


**Figure S5.** (a) NO_x_ conversion, (b) N_2_O concentration and N_2_ selectivity of the Cu-SSZ-13_P and Nd_0.05_-Cu-SSZ-13_P catalysts in NH_3_-SCR. Reaction conditions: 500 ppm NO, 500 ppm NH_3_ and 10% O_2_, 5% H_2_O, 5% CO_2_ with N_2_ as the balance, WHSV = 200, 000 mL·g^-1^·h^-1^.


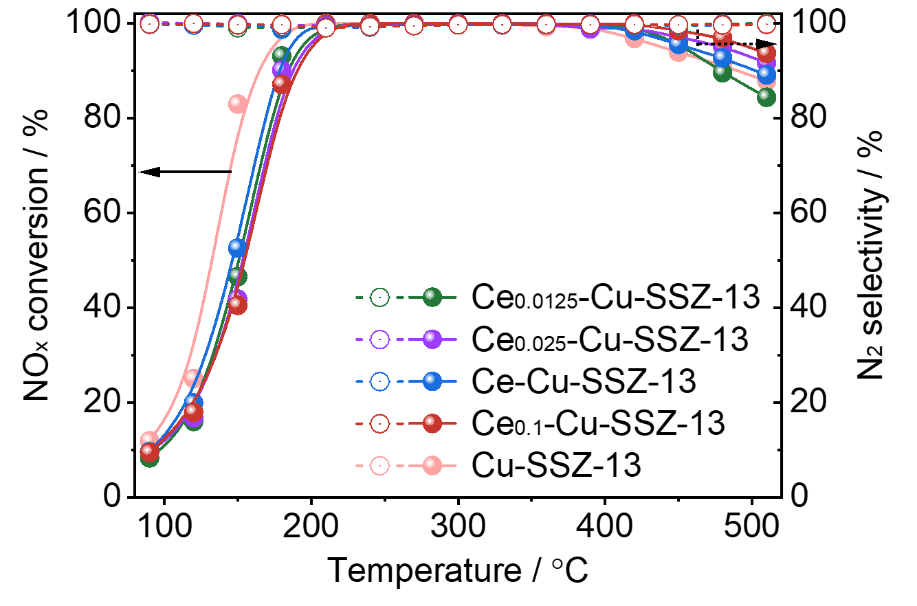


**Figure S6.** NO_x_ conversion and N_2_ selectivity of Ce_0.0125_-Cu-SSZ-13, Ce_0.025_-Cu-SSZ-13, Ce-Cu-SSZ-13, Ce_0.1_-Cu-SSZ-13, and Cu-SSZ-13 catalysts in NH_3_-SCR. Reaction conditions: 500 ppm NO, 500 ppm NH_3_ and 10% O_2_, 5% H_2_O, 5% CO_2_ with N_2_ as the balance, WHSV = 200, 000 mL·g^-1^·h^-1^.(Note: The number next to the Ce (in the formula) is relative to the amount of Ce in solution.)

The Ce-Cu-SSZ-13 catalysts prepared by adjusting the concentration of Ce(NO_3_)_3_ solution exhibit similar NH_3_-SCR performance.


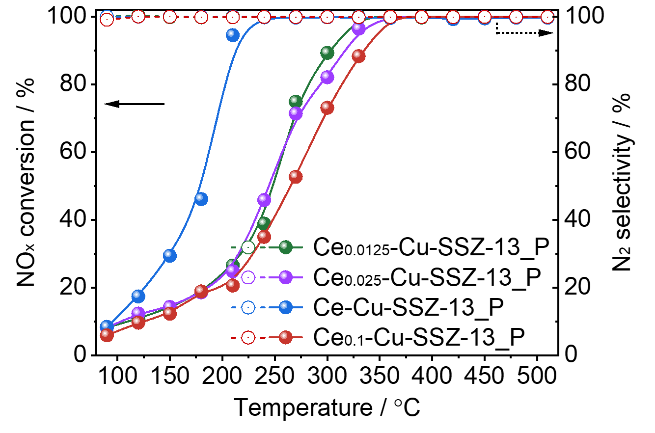


**Figure S7.** NO_x_ conversion and N_2_ selectivity of Ce_0.0125_-Cu-SSZ-13_P, Ce_0.025_-Cu-SSZ-13_P, Ce-Cu-SSZ-13_P and Ce_0.1_-Cu-SSZ-13_P catalysts in NH_3_-SCR. Reaction conditions: 500 ppm NO, 500 ppm NH_3_ and 10% O_2_, 5% H_2_O, 5% CO_2_ with N_2_ as the balance, WHSV = 200, 000 mL·g^-1^·h^-1^.(Note: The number next to the Ce (in the formula) is relative to the amount of Ce in solution.)

The Ce-Cu-SSZ-13_P catalyst has the excellent NH_3_-SCR performance compared with Ce_0.0125_-Cu-SSZ-13_P/ Ce_0.025_-Cu-SSZ-13_P/Ce-Cu-SSZ-13_P. Therefore, Cu-SSZ-13 prepared at Ce(NO_3_)_3_ solution concentration of 0.05M presents the optimal phosphorus resistance.


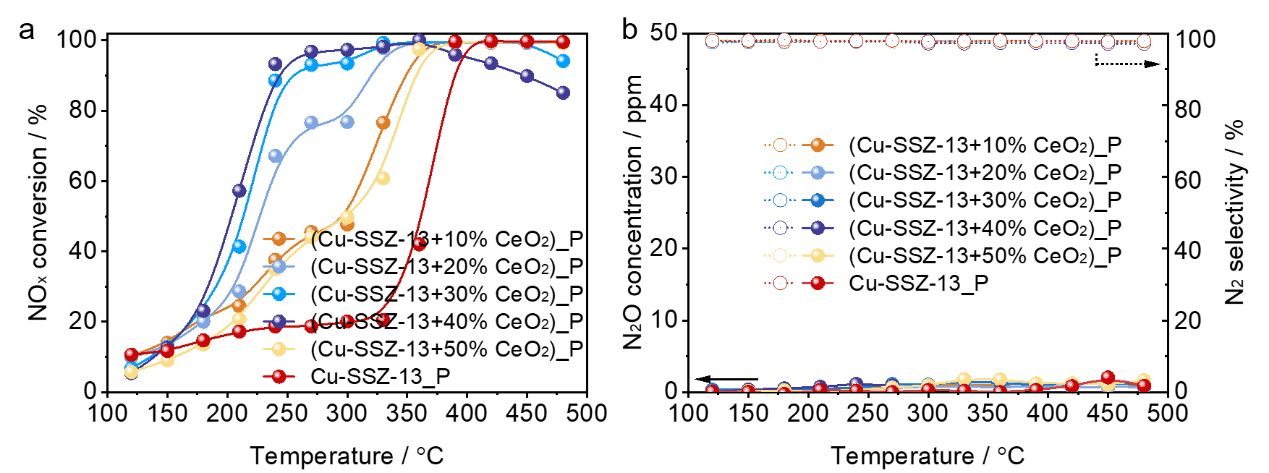


**Figure S8.** (a) NO_x_ conversion, (b) N_2_O concentration and N_2_ selectivity of the (Cu-SSZ-13+zCeO_2_)_P (z = 0%, 10%, 20%, 30%, 40%, 50% in weight percent) catalysts in NH_3_-SCR. Reaction conditions: 500 ppm NO, 500 ppm NH_3_ and 10% O_2_, 5% H_2_O, 5% CO_2_ with N_2_ as the balance, WHSV = 200, 000 mL·g^-1^·h^-1^.

The NOₓ conversion results exhibits a non-linear relationship with the content of CeO_2_, initially increasing to a peak before declining at higher concentrations. The catalyst achieves the optimal resistance to phosphorus poisoning at a CeO₂ content of 40%.


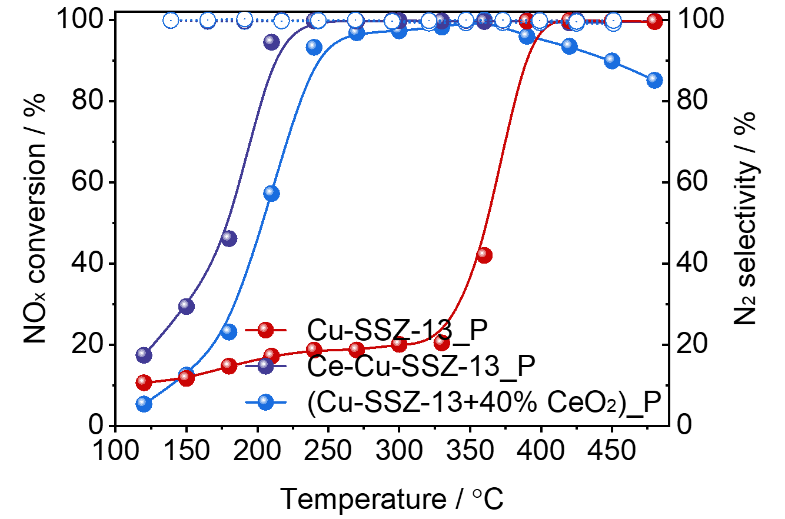


**Figure S9.** NO_x_ conversion and N_2_ selectivity of the Cu-SSZ-13_P, Ce-Cu-SSZ-13_P and Cu-SSZ-13+40%CeO_2_)_P catalysts in NH_3_-SCR. Reaction conditions: 500 ppm NO, 500 ppm NH_3_ and 10% O_2_, 5% H_2_O, 5% CO_2_ with N_2_ as the balance, WHSV = 200, 000 mL·g^-1^·h^-1^.

Compared to the Ce-Cu-SSZ-13_P catalyst achieving 90% NO_x_ conversion at 210 °C, the (Cu-SSZ-13+40% CeO_2_)_P catalyst exhibits the T_90_ (the temperature at which the NO_x_ conversion rate reaches 90%) delayed by 30 °C, indicating that atomically dispersed Ce demonstrates superior phosphorus poisoning suppression compared to CeO_2_.


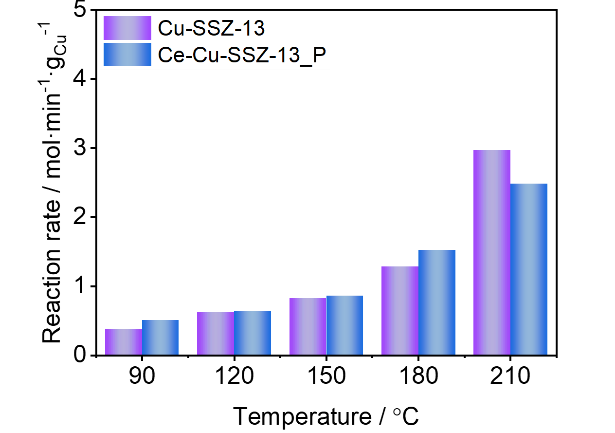


**Figure S10.** The reaction rate histograms of Cu-SSZ-13 and Ce-Cu-SSZ-13_P in the low-temperature range normalised on the Cu atomic content of Cu-SSZ-13. Reaction conditions: 500 ppm NO, 500 ppm NH_3_ and 10% O_2_, 5% H_2_O, 5% CO_2_ with N_2_ as the balance, WHSV = 200, 000 mL·g^-1^·h^-1^.


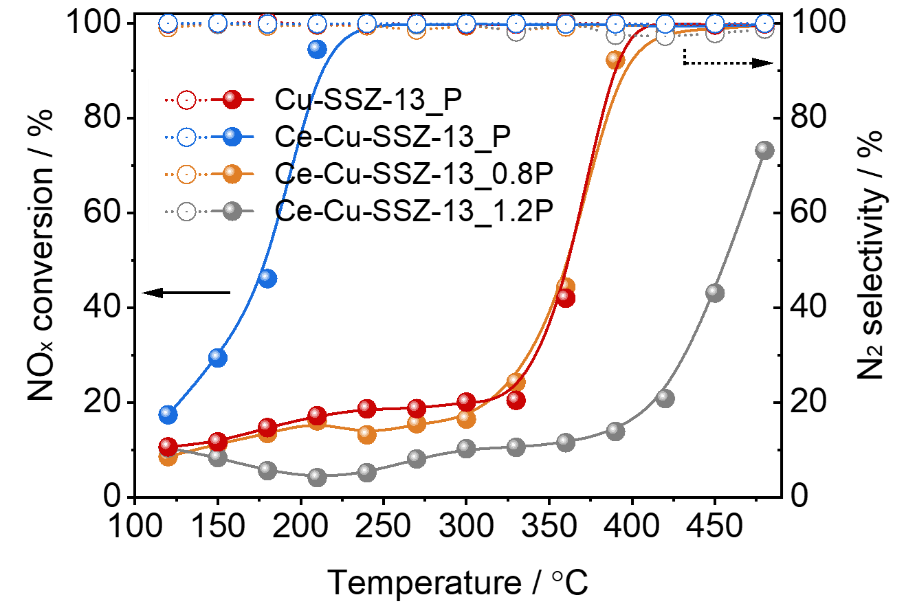


**Figure S11.** NO_x_ conversion and N_2_ selectivity of the Cu-SSZ-13_P, Ce-Cu-SSZ-13_P, Ce-Cu-SSZ-13_0.8P, and Ce-Cu-SSZ-13_1.2P catalysts in NH_3_-SCR. Reaction conditions: 500 ppm NO, 500 ppm NH_3_ and 10% O_2_, 5% H_2_O, 5% CO_2_ with N_2_ as the balance, WHSV = 200, 000 mL·g^-1^·h^-1^.

To provide an estimate of the catalyst lifetime under representative biodiesel exhaust conditions, we test the NH₃-SCR performance of Ce-Cu-SSZ-13 with higher phosphorus content. The results show that NO_x_ conversion of Cu-SSZ-13_P remains below 20% until 330 °C and the Ce-Cu-SSZ-13_0.8P catalyst performs similarly. And the performance of Ce-Cu-SSZ-13_1.2P is significantly inferior to that of Ce-Cu-SSZ-13_0.8P. Thus, Ce-Cu-SSZ-13 exhibits a higher deactivation threshold (0.8 mmol/g_cat_) than that of Cu-SSZ-13 (0.4 mmol/g_cat_).

**
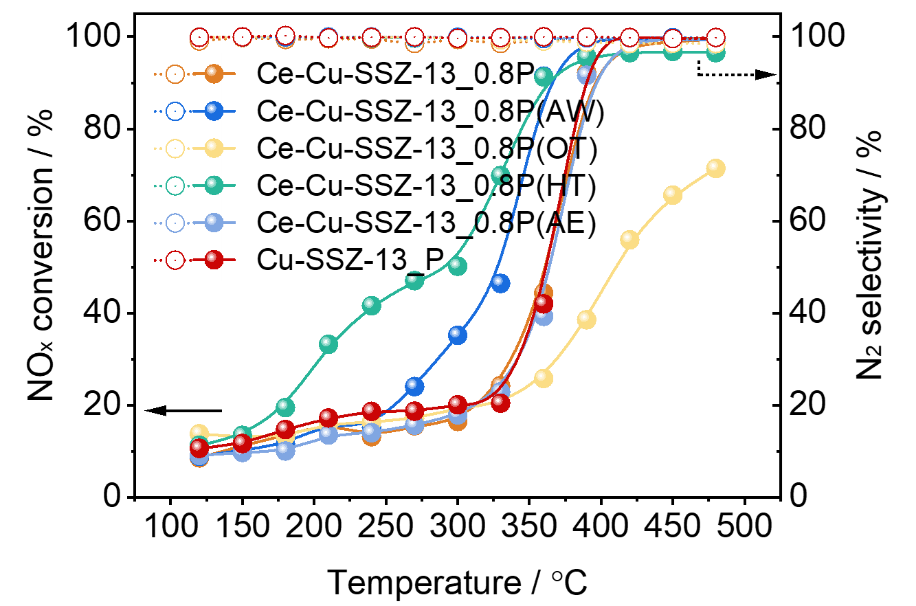
**

**Figure S12.** NO_x_ conversion and N_2_ selectivity of the Cu-SSZ-13_P, Ce-Cu-SSZ-13_0.8P and Ce-Cu-SSZ-13_0.8P (AW/OT/HT/AE) catalysts in NH_3_-SCR. Reaction conditions: 500 ppm NO, 500 ppm NH_3_ and 10% O_2_, 5% H_2_O, 5% CO_2_ with N_2_ as the balance, WHSV = 200, 000 mL·g^-1^·h^-1^.

To verify which kind of regeneration method is useful for the deactivated Cu-Ce-SSZ-13 catalysts, we treated the deactivated Ce-Cu-SSZ-13_0.8P catalyst via oxidation treatment (OT, a 2 h pre-treatment in 10% O_2_/N_2_), hydrothermal treatment (HT, 50 h of hydrothermal aging at 650°C), mild acid washing (AW, a 1 h wash with 0.1 M H_2_C_2_O_4_(aq)), and ammonium salt exchange (AE, a 2 h ion exchange with 1 M NH_4_Cl solution at 80°C), and assesses the corresponding regenerated NH_3_-SCR activity. The results show that mild acid washing and hydrothermal treatment effectively restore the low-temperature activity of the deactivated Ce-Cu-SSZ-13 catalyst.


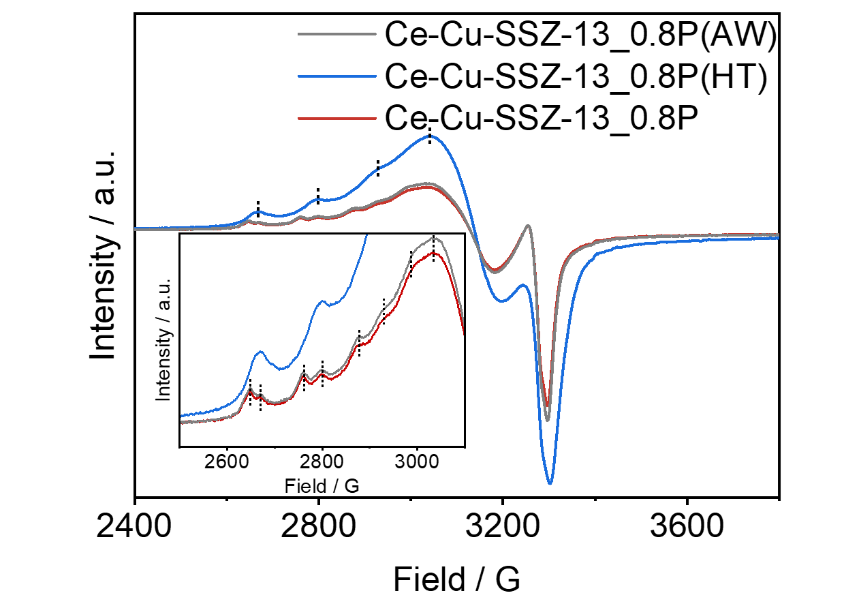


**Figure S13.** EPR profiles of Ce-Cu-SSZ-13_0.8P, Ce-Cu-SSZ-13_0.8P(HT), Ce-Cu-SSZ-13_0.8P(AW) at room temperature (insert is the magnification of the EPR profiles within the range of 2500-3100G).

EPR analysis reveals that the regenerated Ce-Cu-SSZ-13_0.8P catalyst, especially after hydrothermal treatment, exhibits a higher concentration of active Cu^2+^ species. The restored Cu^2+^ quadruple peak confirms the effective mitigation of phosphorus poisoning by the regeneration treatment.


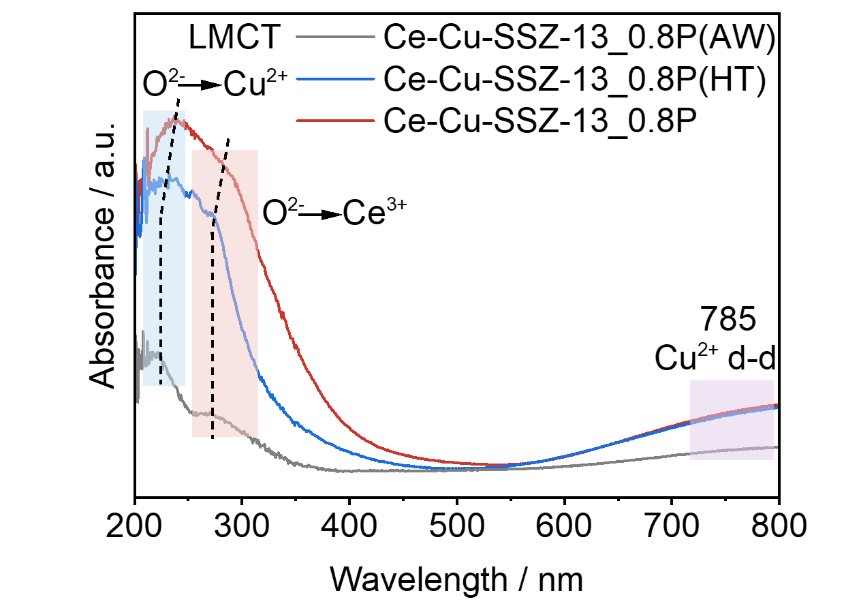


**Figure S14.** UV-vis profiles of Ce-Cu-SSZ-13_0.8P, Ce-Cu-SSZ-13_0.8P(HT), Ce-Cu-SSZ-13_0.8P(AW).

UV-vis spectra show a blue shift in the absorption bands corresponding to O^2^⁻ → Cu^2+^ and O^2^⁻ → Ce^3+^ transitions in the regenerated samples, indicating stronger interaction between the Cu^2+^/Ce^3+^ sites and the zeolite framework O species.


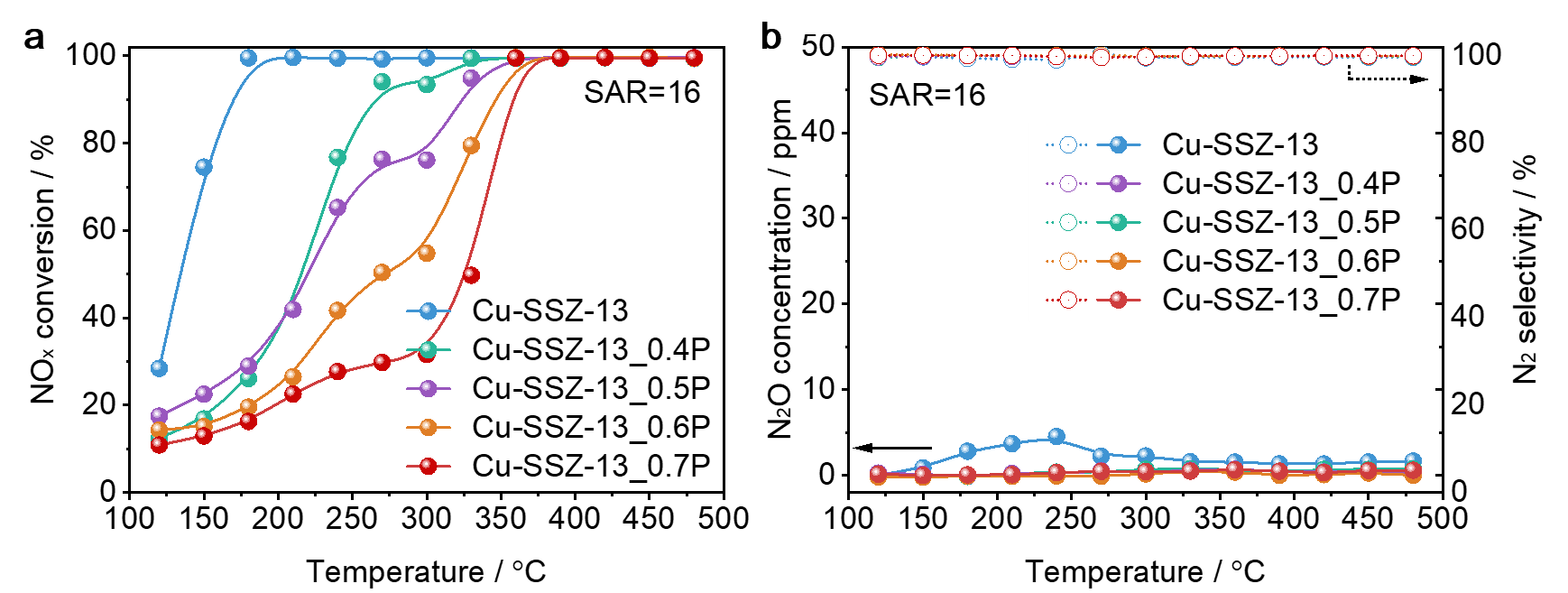


**Figure S15.** (a) NO_x_ conversion, (b) N_2_O concentration and N_2_ selectivity of the Cu-SSZ-13_yP (y=0.4, 0.5, 0.6, 0.7 mmol/g_cat_) (SAR=16) catalysts in NH_3_-SCR. Reaction conditions: 500 ppm NO, 500 ppm NH_3_ and 10% O_2_, 5% H_2_O, 5% CO_2_ with N_2_ as the balance, WHSV = 200, 000 mL·g^-1^·h^-1^.

The Ce ion modification strategy is applied to Cu-SSZ-13 (SAR=16) with lower silicon-to-aluminium ratios (SAR=16), revealing that a higher phosphorus content (0.7 mmol/g_cat_) is required to achieve complete deactivation.


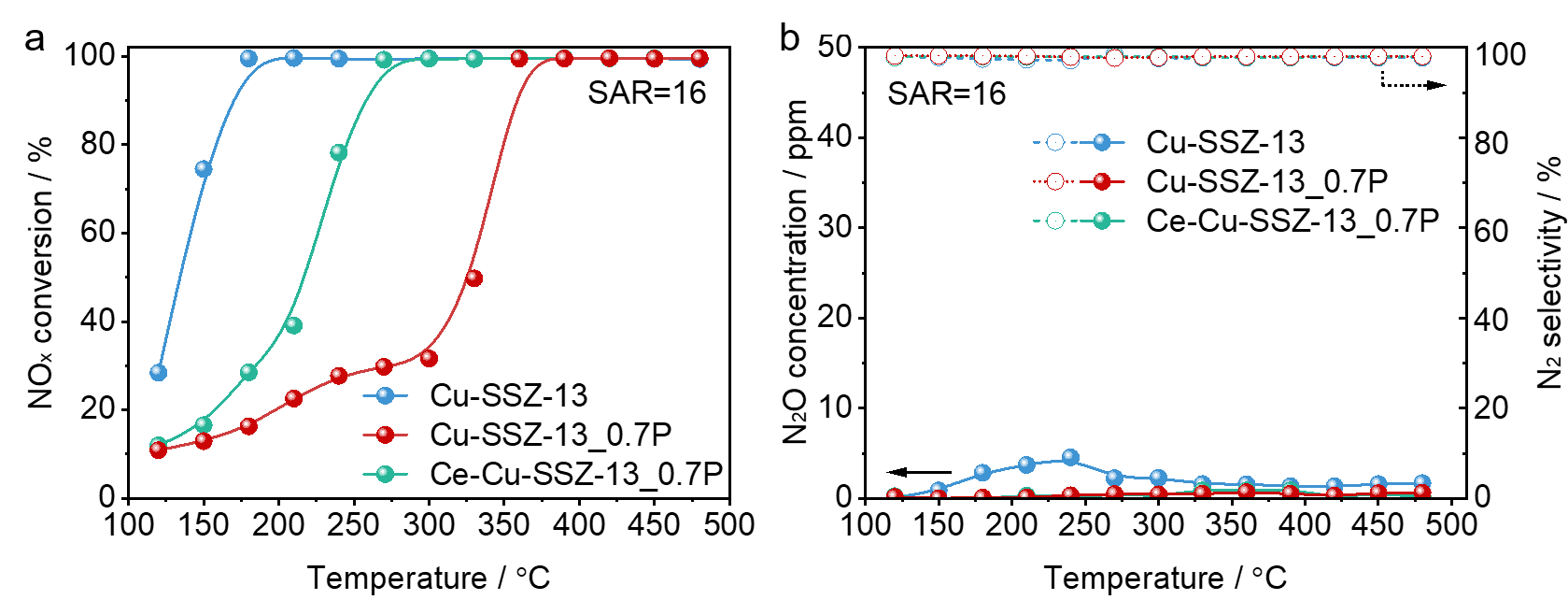


**Figure S16.** (a) NO_x_ conversion, (b) N_2_O concentration and N_2_ selectivity of the Cu-SSZ-13, Cu-SSZ-13_0.7P and Ce-Cu-SSZ-13_0.7P (SAR=16) catalysts in NH_3_-SCR. Reaction conditions: 500 ppm NO, 500 ppm NH_3_ and 10% O_2_, 5% H_2_O, 5% CO_2_ with N_2_ as the balance, WHSV = 200, 000 mL·g^-1^·h^-1^.

Cu-SSZ-13_0.7P (SAR=16) is selected to evaluate the Ce ion modification strategy on imparting phosphorus resistance to zeolite catalysts with different SAR. The Ce ion modified Cu-SSZ-13 (SAR=16) with 2.23 wt% Ce added via solid-state ion exchange method significantly enhances its NOₓ conversion under higher content of phosphorus poisoning conditions, confirming the universality of the Ce ion modification strategy on promoting phosphorus resistance of Cu-SSZ-13 catalysts with different silicon-to-aluminium ratios.


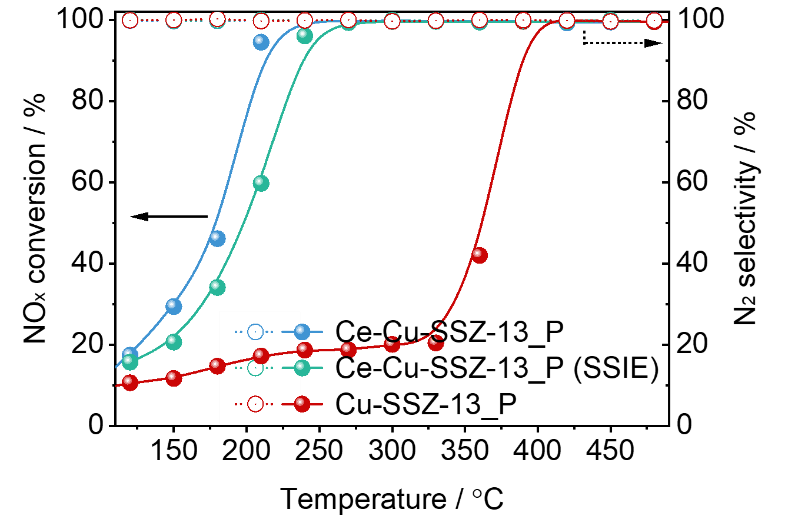


**Figure S17.** NO_x_ conversion and N_2_ selectivity of the Cu-SSZ-13_P, Ce-Cu-SSZ-13_P and Ce-Cu-SSZ-13_P (SSIE) catalysts in NH_3_-SCR. Reaction conditions: 500 ppm NO, 500 ppm NH_3_ and 10% O_2_, 5% H_2_O, 5% CO_2_ with N_2_ as the balance, WHSV = 200, 000 mL·g^-1^·h^-1^.

Cu-Ce dual-atom catalyst prepared via solid-state ion exchange (SSIE) only shows slightly lower phosphorus tolerance than those synthesized by liquid-state ion exchange. After the same content of phosphorus exposure, the Ce-Cu-SSZ-13_P (SSIE) catalyst maintains 90% NOₓ conversion efficiency and 100% N₂ selectivity at 240 °C.


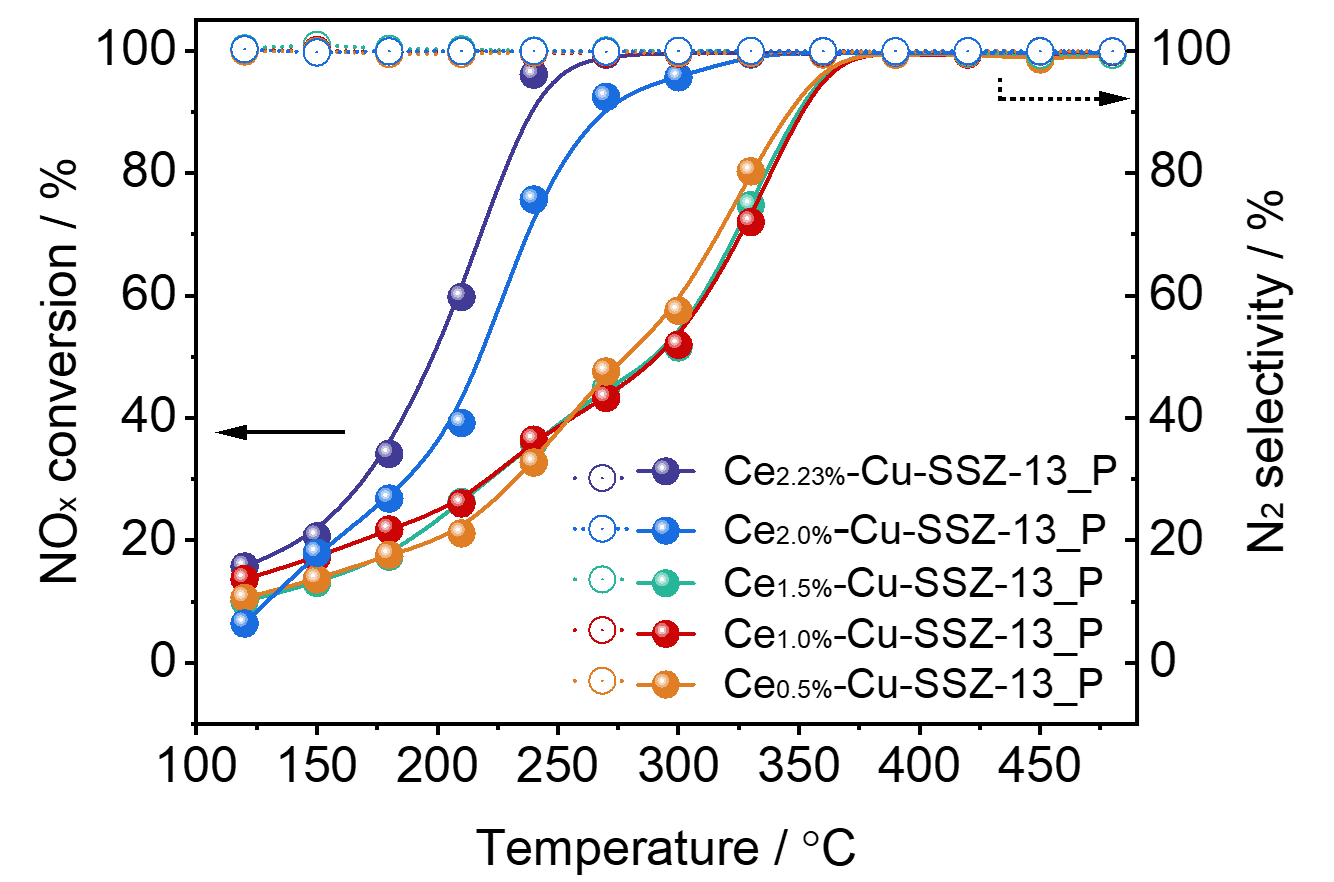


**Figure S18.** NO_x_ conversion and N_2_ selectivity of the (Ce_w_-Cu-SSZ-13)_P(SSIE) (w=0.5%, 1.0%, 1.5%, 2.0%, 2.23% in weight percent) catalysts in NH_3_-SCR. Reaction conditions: 500 ppm NO, 500 ppm NH_3_ and 10% O_2_, 5% H_2_O, 5% CO_2_ with N_2_ as the balance, WHSV = 200, 000 mL·g^-1^·h^-1^.

To verify the cost implications of the Ce loading (2.23 wt% Ce) among Ce-Cu-SSZ-13 catalysts and whether similar protection can be achieved at lower Ce contents, the Ce-Cu-SSZ-13 catalysts with varying Ce contents (0.5%, 1.0%, 1.5%, 2.0%, 2.23% in weight percent) are synthesized by to precisely control the Ce content incorporated into Cu-SSZ-13 catalysts. Then, the NH_3_-SCR performance of (Ce_w_-Cu-SSZ-13)_P(SSIE) (w=0.5%, 1.0%, 1.5%, 2.0%, 2.23% in weight percent) catalysts are evaluated after identical P poisoning, and the NO_x_ conversion is correlated with the Ce weight percentage.


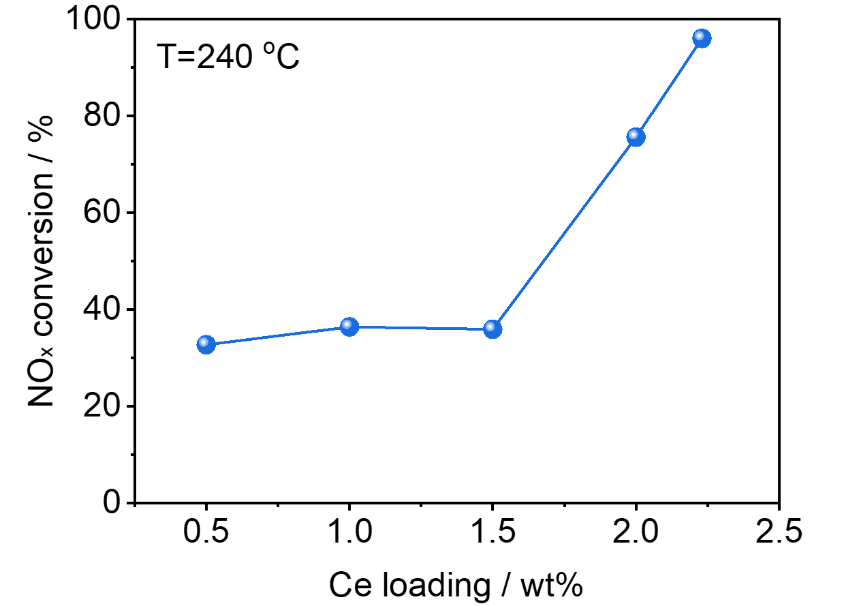


**Figure S19.** Plots of the NO_x_ conversion *versus* Ce loading over (Ce_w_-Cu-SSZ-13)_P(SSIE) (w=0.5%, 1.0%, 1.5%, 2.0%, 2.23% in weight percent) catalysts in NH_3_-SCR at 240 °C.

The results indicate that as the Ce content decreased, the catalytic performance first declines (Ce > 1.5%) and then stabilizes (Ce < 1.5%), suggesting that the Ce content of 2.23% used in this study represents an optimal balance between catalytic performance and cost.


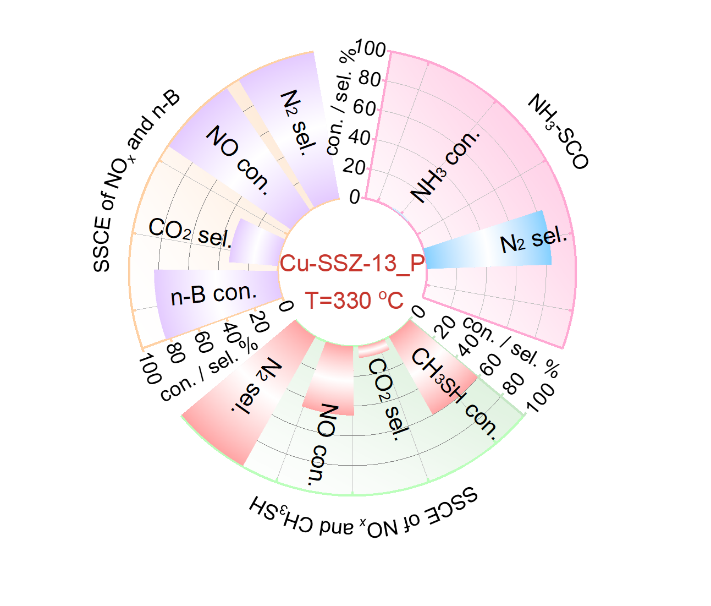


**Figure S20.** Performance evaluation of Cu-SSZ-13_P in other environmental catalytic reactions (T=330 ^o^C). Reaction conditions: 500 ppm NH_3_, 10 % O_2_, 5% H_2_O, N_2_ as balance, and WHSV = 200, 000 mL·g^-1^·h^-1^. 500 ppm NO, 500 ppm NH_3_, 100 ppm CH_3_SH, 5 % O_2_, N_2_ as balance, and GHSV = 25,000 h^−1^ for SSCE of NO_x_ and CH_3_SH. 100 ppm NO, 100 ppm NH_3_, 200 ppm n-B, 5 % O_2_, N_2_ as balance, and GHSV = 50,000 h^−1^ for SSCE of NO_x_ and n-B.


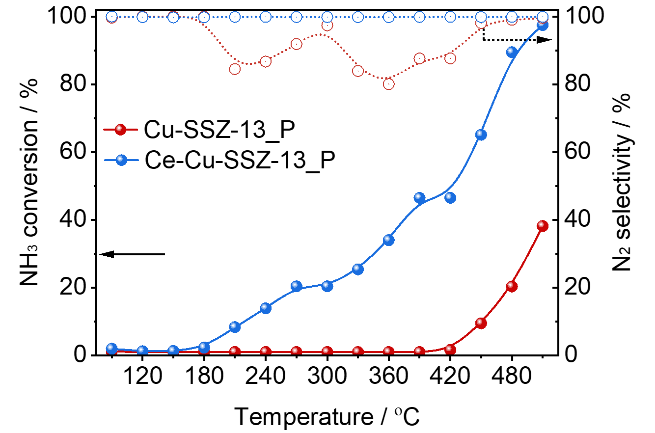


**Figure S21.** NH_3_ conversion and N_2_ selectivity of Cu-SSZ-13_P and Ce-Cu-SSZ-13_P catalysts in NH_3_-SCO. Reaction conditions: 500 ppm NH_3_, 10% O_2_, 5% H_2_O, with N_2_ as the balance, WHSV = 200, 000 mL·g^-1^·h**^-1^.**

It can be seen that Ce incorporation shows a promotional effect on the NH_3_ oxidation activity compare with Cu-SSZ-13_P. Moreover, Ce-Cu-SSZ-13_P can preserve 100% N_2_ selectivity in the whole test temperature ramp from 90 to 510 ^o^C..


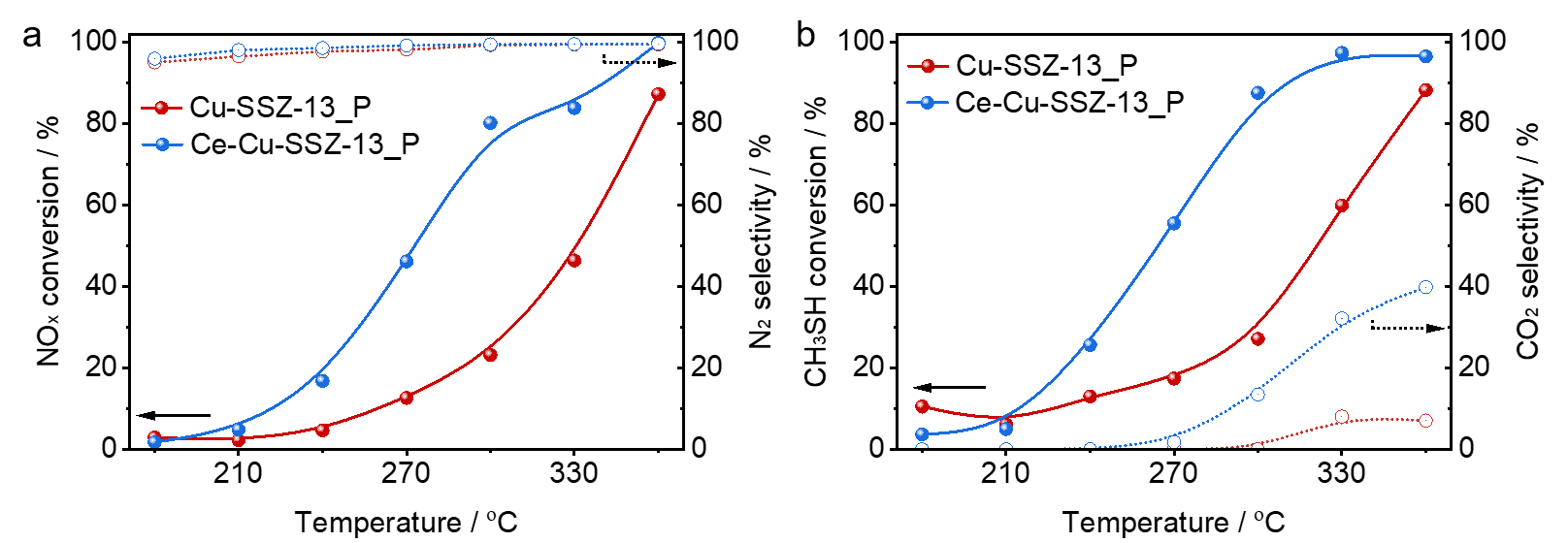


**Figure S22.**  (a) NO*_x_* conversion and N_2_ selectivity, (b) CH_3_SH conversion and CO_2_ selectivity of Cu-SSZ-13_P and Ce-Cu-SSZ-13_P catalysts in SSCE of NO_x_ and CH_3_SH. Reaction conditions: 500 ppm NO, 500 ppm NH_3_, 100 ppm CH_3_SH, 5 vol % O_2_, N_2_ as the balance gas, and GHSV of 25,000 h^–1^.

Ce-Cu-SSZ-13_P catalyst performs a superior catalytic activity in both NO_x_ reduction and CH_3_SH oxidation than that of Cu-SSZ-13_P catalyst. In addition, N_2_ selectivity and CO_2_ yield of Ce-Cu-SSZ-13_P catalyst during the whole test range is higher than that of the Cu-SSZ-13_P catalyst.


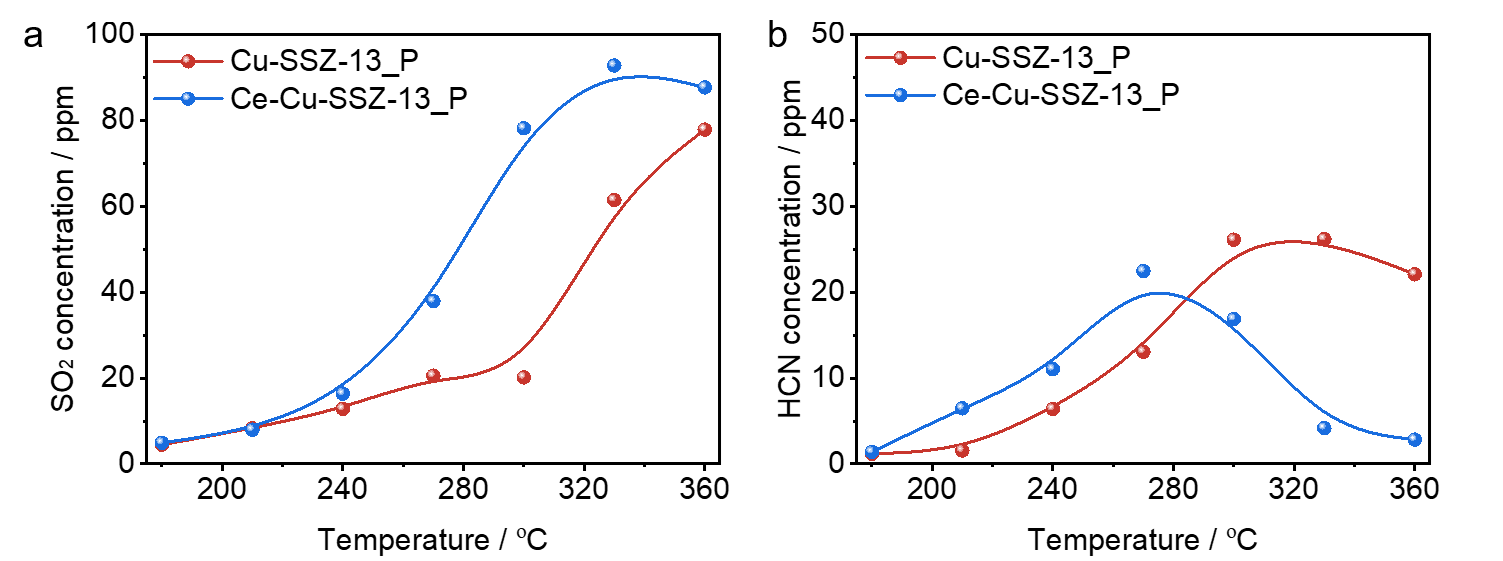


**Figure S23.** The reaction products of (a) SO_2_ and (b) HCN formation over Cu-SSZ-13_P and Ce-Cu-SSZ-13_P catalysts in SSCE of NO_x_ and CH_3_SH. Reaction conditions: 500 ppm NO, 500 ppm NH_3_, 100 ppm CH_3_SH, 5 vol % O_2_, N_2_ as the balance gas, and GHSV of 25,000 h^–1^.

The consistency of SO_2_ yield with the conversion of CH_3_SH indicates that no other sulphur-containing by-products are generated from the Ce-Cu-SSZ-13_P catalyst. In addition, the HCN yields of two catalysts is close to each other in the low-temperature interval, but Cu-SSZ-13_P apparently has more HCN production in the high-temperature interval.


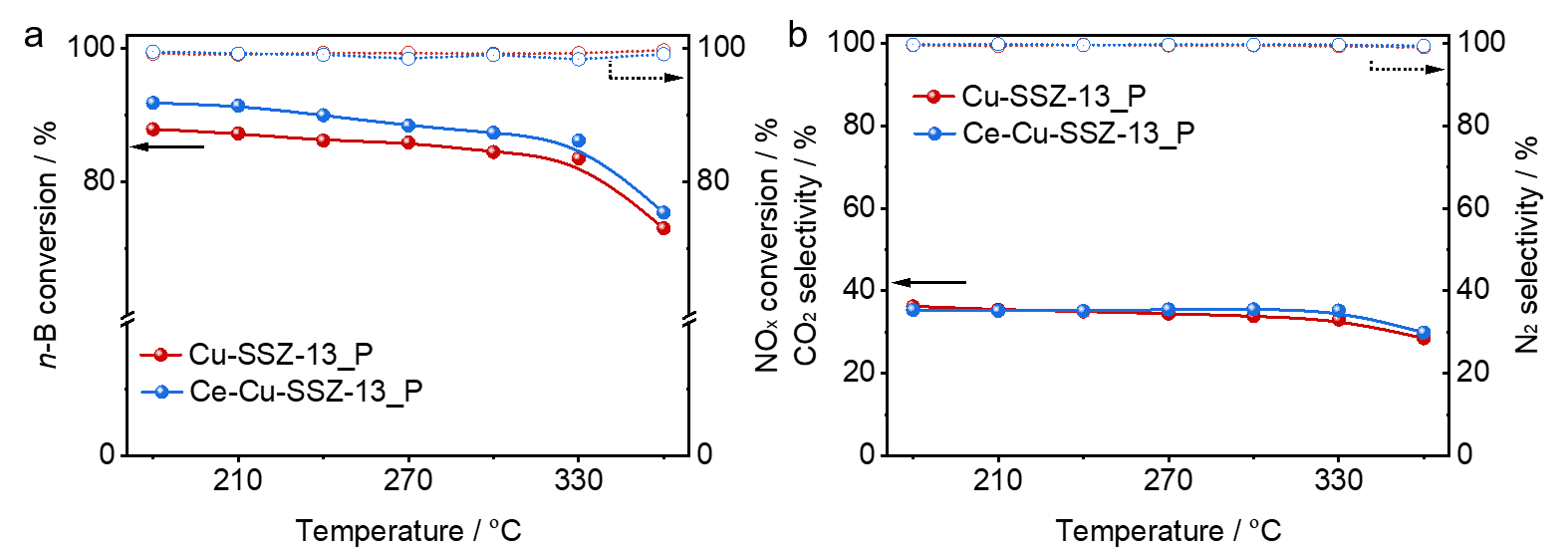


**Figure S24.** (a) n-B conversion and NO*_x_* conversion, (b) CO_2_ selectivity and N_2_ selectivity of Cu-SSZ-13_P and Ce-Cu-SSZ-13_P catalysts in SSCE of NO_x_ and n-B. Reaction conditions: 100 ppm NO, 100 ppm NH_3_, 200 ppm n-B, 5 vol % O_2_, N_2_ as the balance gas, and GHSV of 50,000 h^–1^.

Both catalysts show 100% NO_x_ conversion and N_2_ selectivity from 180 to 360 ^o^C. However, Ce-Cu-SSZ-13_P has higher n-butylamine conversion and CO_2_ yield.


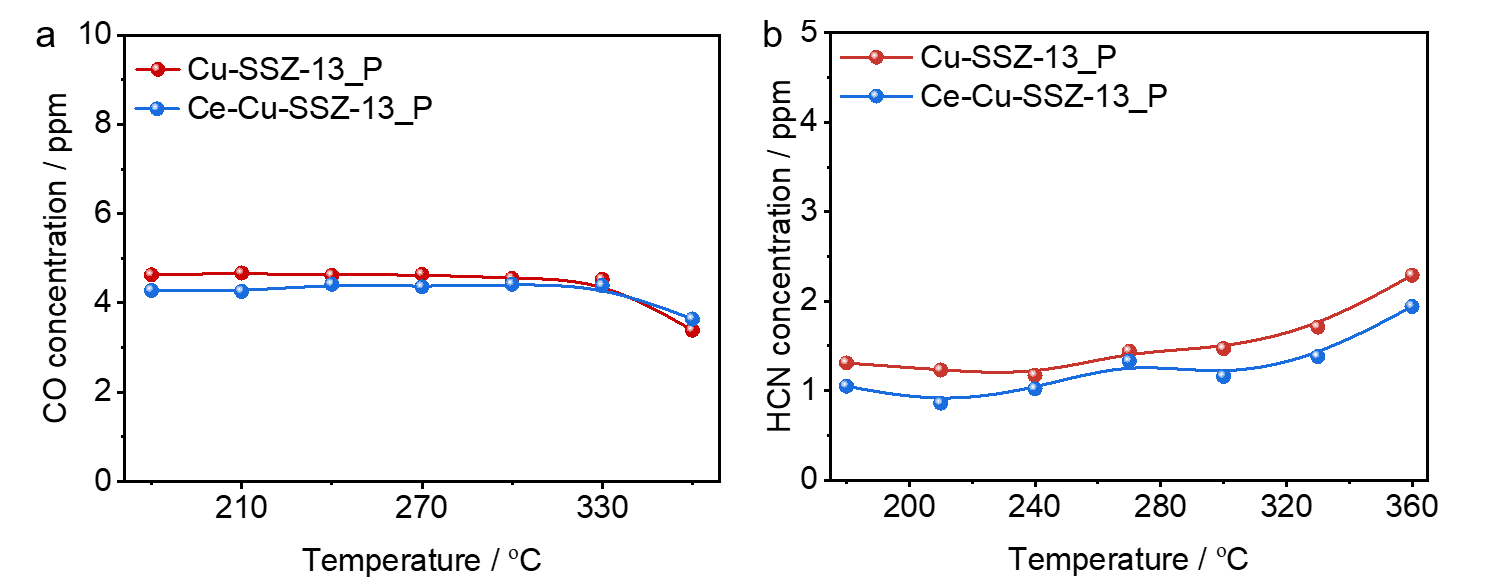


**Figure S25.** The reaction products of (a) CO and (b) HCN formation over Cu-SSZ-13_P and Ce-Cu-SSZ-13_P catalysts in SSCE of NO_x_ and n-B. Reaction conditions: 100 ppm NO, 100 ppm NH_3_, 200 ppm n-B, 5 vol % O_2_, N_2_ as the balance gas, and GHSV of 50,000 h^–1^.

Cu-SSZ-13_P catalyst shows higher CO and HCN yield than that of Ce-Cu-SSZ-13_P catalyst.


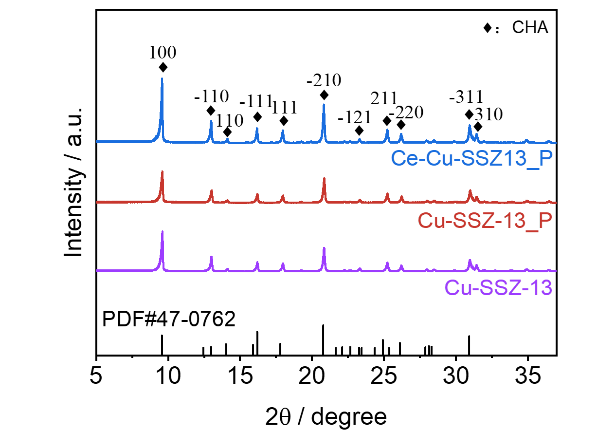


**Figure S26.** XRD patterns of the Cu-SSZ-13, Cu-SSZ-13_P and Ce-Cu-SSZ-13_P catalysts.


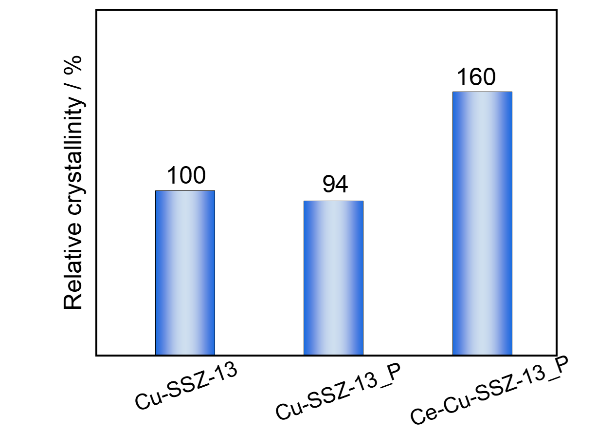


**Figure S27.** Relative crystallinity of the Cu-SSZ-13, Cu-SSZ-13_P and Ce-Cu-SSZ-13_P catalysts.


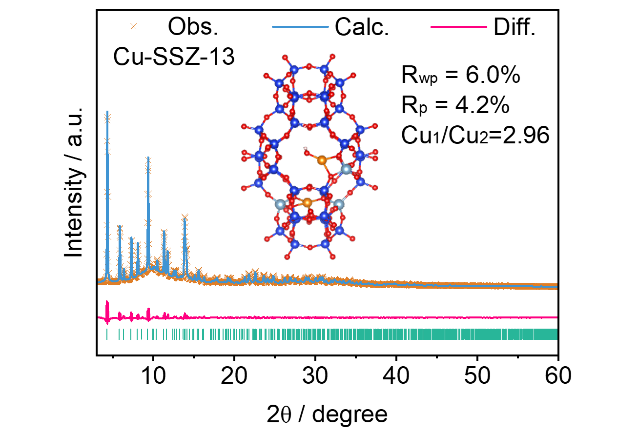


**Figure S28.** Crystallographic structure of Cu-SSZ-13 with 2.37 wt% Cu loading and its corresponding final Rietveld refinement results by using synchrotron X-ray scattering data. The observed, calculated, and difference curves are in orange, blue, and pink, respectively. The vertical bars indicate the positions of the Bragg peaks (λ=6.889 nm).


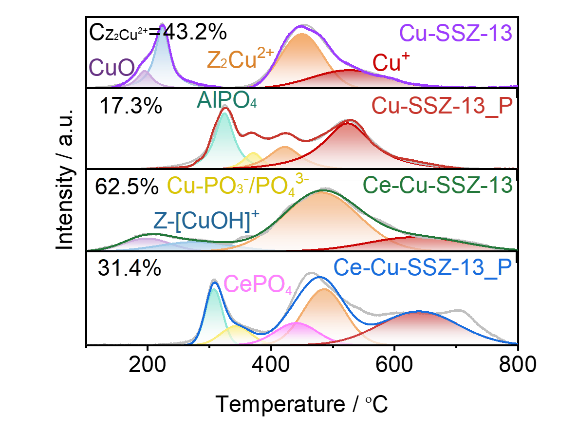


**Figure S29.** H_2_-TPR profiles of the Cu-SSZ-13, Cu-SSZ-13_P, Ce-Cu-SSZ-13 and Ce-Cu-SSZ-13_P catalysts.

For the Cu-SSZ-13 catalyst, the reduction peak at 198 °C corresponds to reduction of CuO species to Cu^0^, 223 °C and 449 °C are attributable to reduction of [ZCu^2+^OH]^+^ in 8MRs and Z_2_Cu^2+^ in 6MRs, respectively, and the reduction peak above 500 °C is attributable to reduction of Cu^+^ to Cu^0^.^[5]^ The new peaks among Cu-SSZ-13 after phosphorus impregnation appear at 324 °C and 370 °C are attributed to reduction of phosphorus or oxygen species (dealuminized by phosphorus introduction) and reduction of Cu with phosphate/metaphosphates complex, respectively.^[6]^ At the same time, the [ZCu^2+^OH]^+^ sites locate in the 8MRs almost disappear, which confirms that the [ZCu^2+^OH]^+^ sites bond to phosphate/metaphosphate, while phosphorus species also affects the reduction of Z_2_Cu^2+^ sites.

Compared with Cu-SSZ-13, the amount of [ZCu^2+^OH]^+^ at 277 °C decreases and the amount of Z_2_Cu^2+^ at 481 °C increases in Ce-Cu-SSZ-13, revealing that Ce cations can increase the relative proportion of Z_2_Cu^2+^.^[7]^ With the subsequent phosphorus poisoning, the reduction behaviors of Ce-Cu-SSZ-13_P shows a trace amount of characteristic peaks of CePO_4_ species around 450 °C.^[8]^


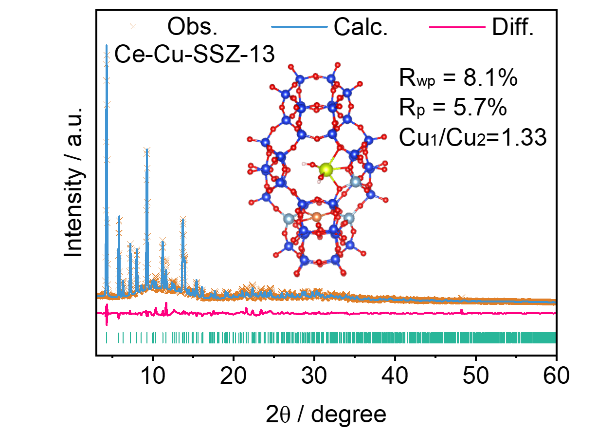


**Figure S30.** Crystallographic structure of Ce-Cu-SSZ-13 with 1.88 wt% Cu, 2.41 wt% Ce loading and its corresponding final Rietveld refinement results by using synchrotron X-ray scattering data. The observed, calculated, and difference curves are in black, red, and blue, respectively. The vertical bars indicate the positions of the Bragg peaks (λ=6.889 nm).


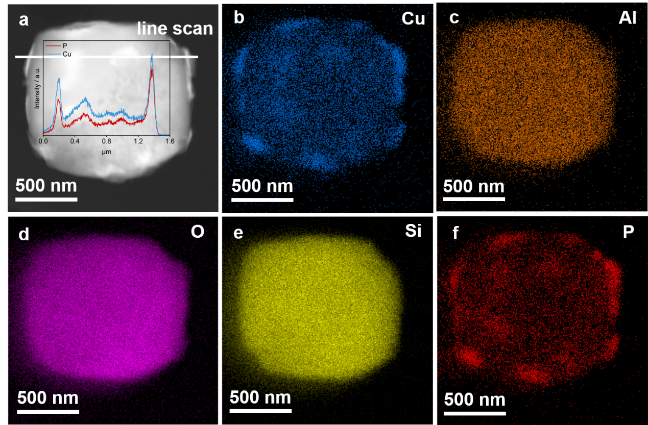


**Figure S31.** (a) HR-TEM image and line scan results of the Cu-SSZ-13_P catalyst. (b-f) EDS mapping results of Cu, Al, O and Si elements distribution over the Cu-SSZ-13_P catalyst (blue represents Cu, orange represents Al, purple represents O, yellow represents Si, and red represents P).


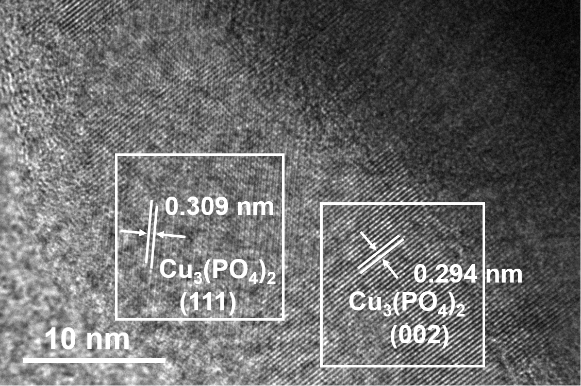


**Figure S32.** HR-TEM image of the Cu-SSZ-13_P catalyst.


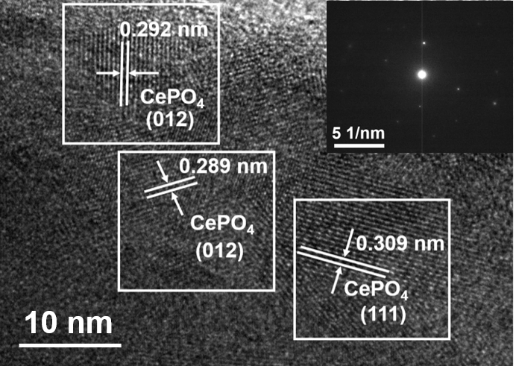


**Figure S33.** HR-TEM image of the Ce-Cu-SSZ-13_P catalyst.


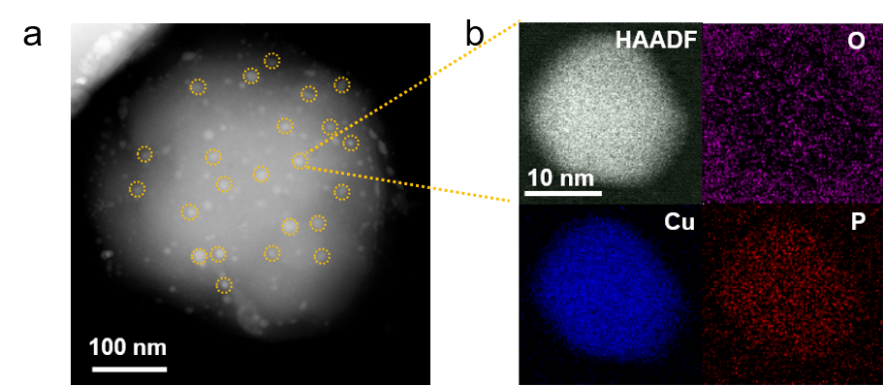


**Figure S34.** (a) AC-HAADF-STEM pictures and (b) HAADF-STEM-EDS mapping results of the Cu-SSZ-13_P catalyst.

To achieve a precise elucidation of the Cu-SSZ-13_P structure, an aberration-corrected high-angle annular dark-field scanning transmission electron microscopy (HAADF-STEM) measurement was conducted. As depicted in Figure S34a, a prominent aggregation of surface particles is evident and Cu_3_(PO_4_)_2_ is formed. Furthermore, HAADF-STEM-EDS analysis unequivocally confirms the coexistence of Cu and P within the Cu-SSZ-13_P catalyst in Figure S34b.


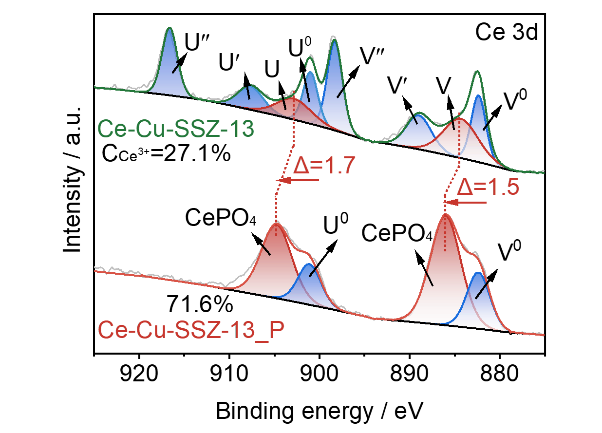


**Figure S35.** Ce 3d XPS spectra of the Ce-Cu-SSZ-13 and Ce-Cu-SSZ-13_P catalysts.

The characteristic peaks of Ce^4+^ in Ce-Cu-SSZ-13 are labeled as v^0^(882.3 eV), v'(888.9 eV), v"(898.3 eV), u^0^(901.0 eV), u'(907.6 eV) and u"(916.6 eV), respectively, and Ce^3+^ is labeled v(884.3eV) and u (902.8 eV).^[9]^ The binding energy of Ce moves to higher value after impregnation of phosphorus, indicating an electron-induced effect between Ce and P. The introduction of phosphorus increases the amount of Ce^3+^, where the appearance of phosphate/metaphosphate species may change the status of Ce.


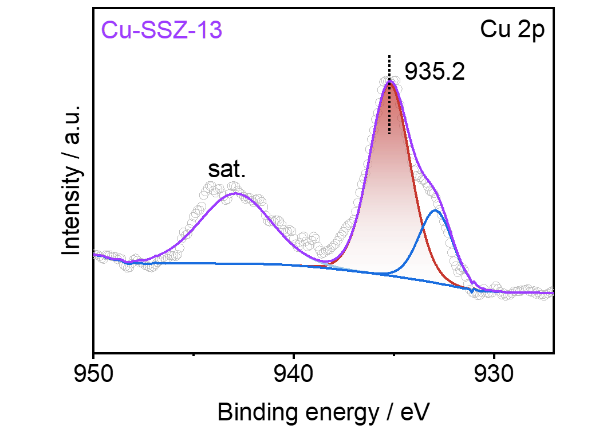


**Figure S36.** Cu 2p XPS spectra of the Cu-SSZ-13 catalyst.

^
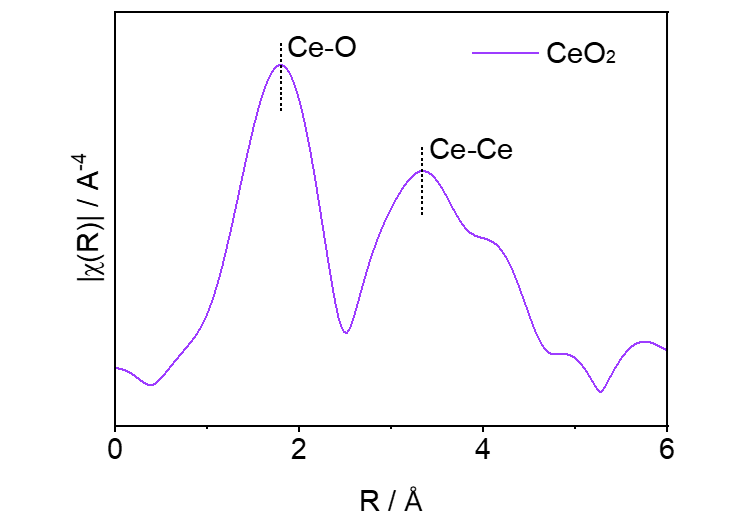
^

**Figure S37.** FT-EXAFS spectra of CeO_2_.

A peak near 3.6 Å is also observed in the FT-EXAFS spectrum of the CeO_2_ reference sample, indicating the distance between Ce-Ce.^[10]^


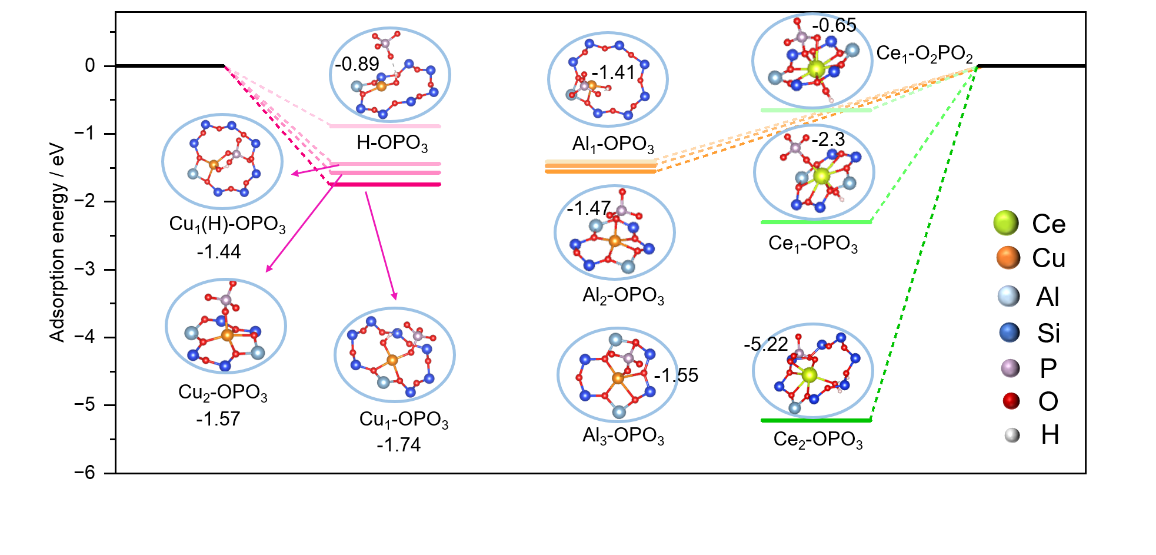


**Figure S38.** Local structures and energy profiles of PO_4_^3-^ adsorption on Cu^2+^ sites, Al^3+^ sites and Ce^3+^ sites. All energies in this are given in eV.


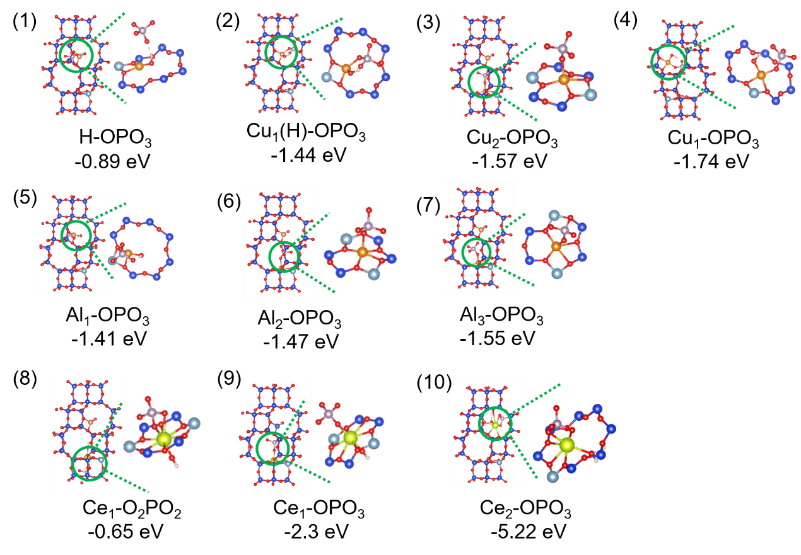


**Figure S39.** Global structures and energy profiles of PO_4_^3-^ adsorption on (1-4) Cu^2+^, (5-7) Al^3+^ and (8-10) Ce^3+^ sites. All energies in this are given in eV.


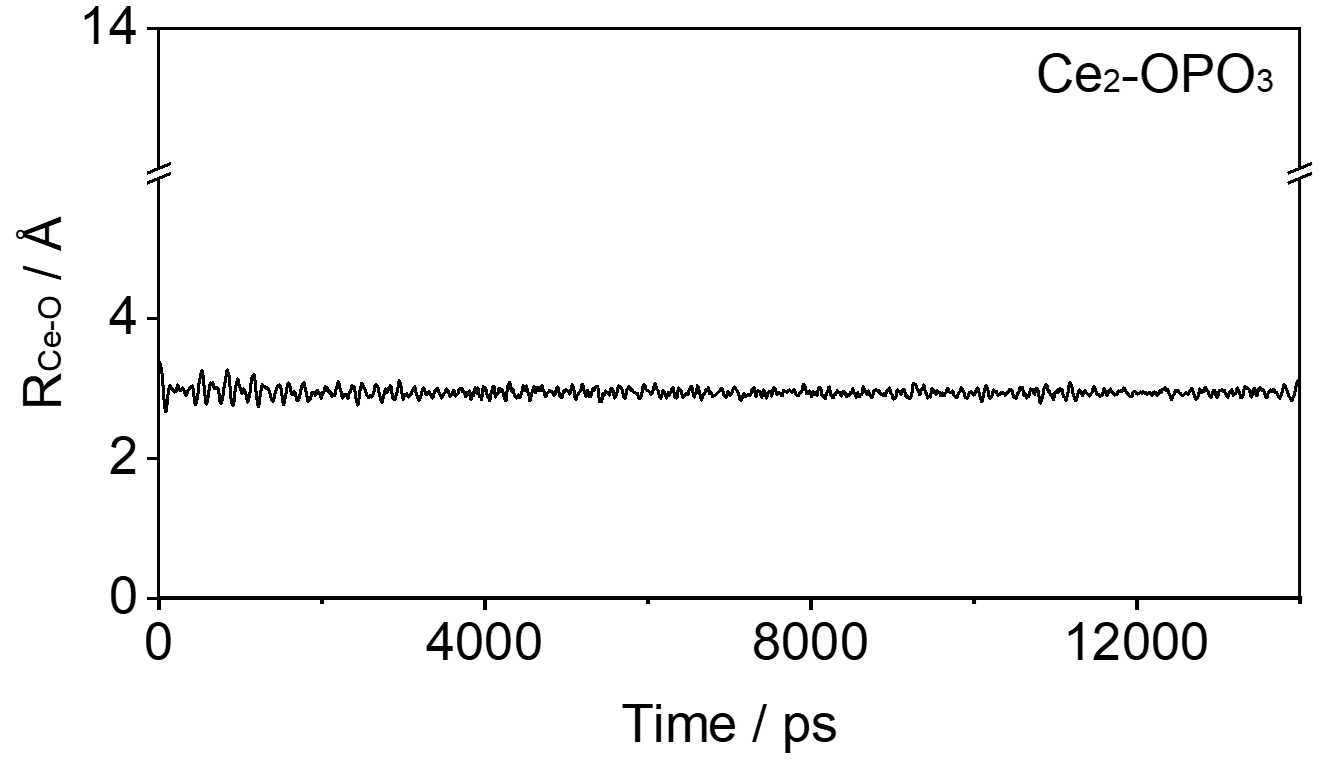


**Figure S40.** Calculated time-dependent distance between the PO_4_^3-^ and Ce sites over the structure of Ce_2_-OPO_3_ in AIMD simulation.


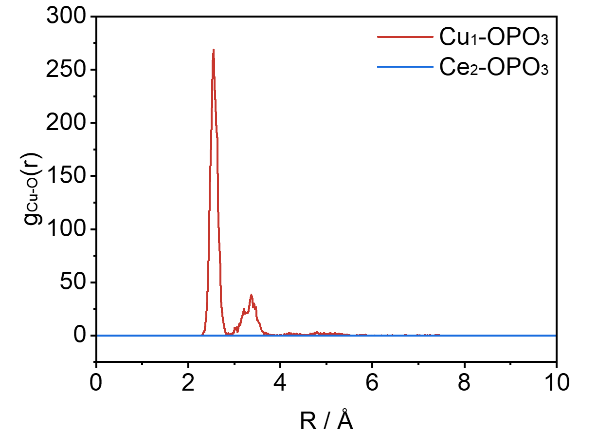


**Figure S41.** RDFs for Cu-O (Cu_1_-OPO_3_) and Cu-O (Ce_2_-OPO_3_) collected from MD simulations.


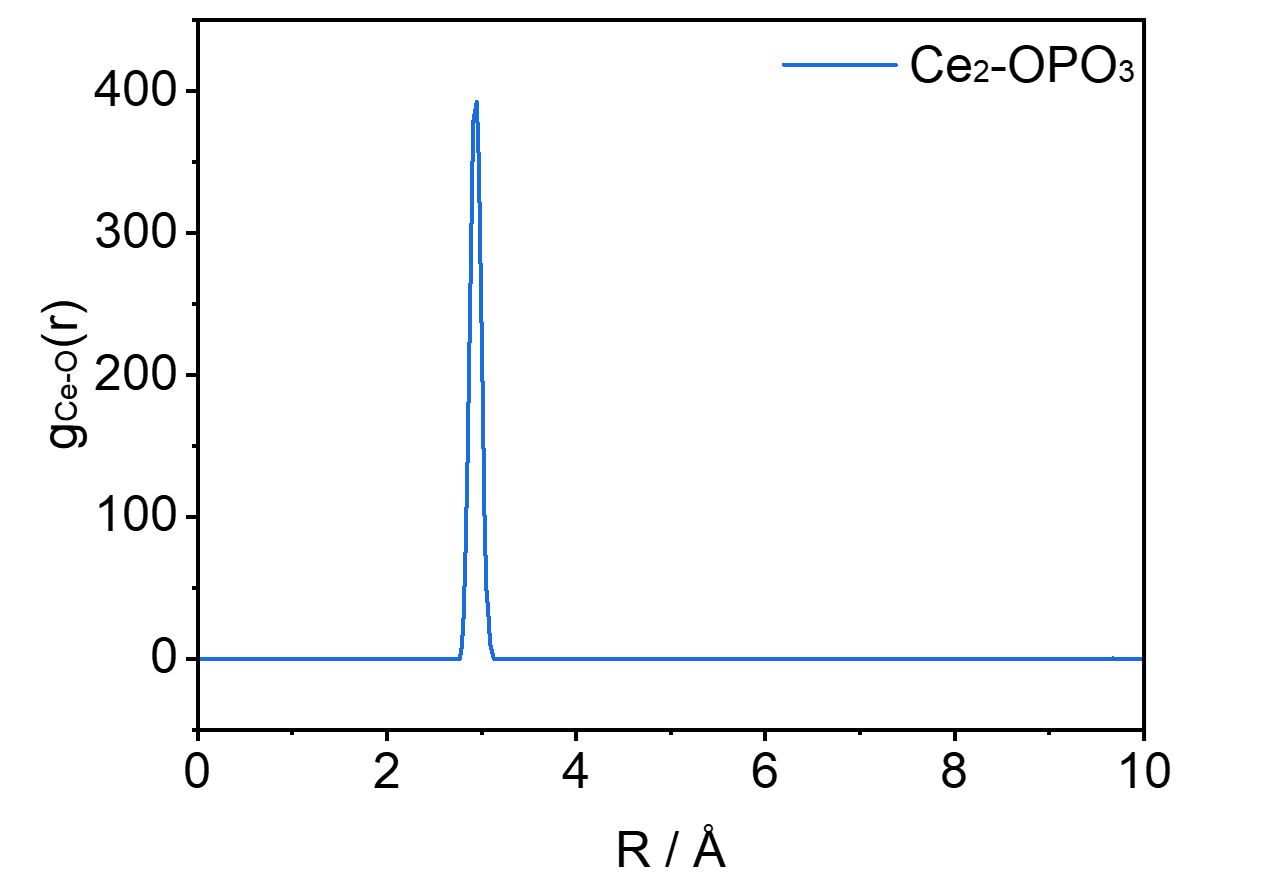


**Figure S42.** RDFs for Ce-O (Ce_2_-OPO_3_) collected from MD simulations.


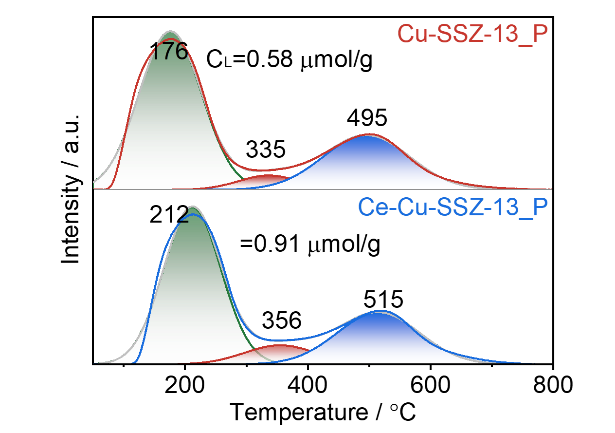


**Figure S43.** NH_3_-TPD-MS profiles of the Cu-SSZ-13_P and Ce-Cu-SSZ-13_P catalysts.

The peak of NH_3_-TPD-MS can be attributed to the desorption of NH_3_ around 180 °C by weak acid sites consisting of terminal hydroxyl groups and structural vacancies, that around 330 °C by medium-strong acid sites consisting of Cu^2+^, as well as that around 490 °C by Brønsted acid sites consisting of Al-OH-Si.^[11]^ The recovery of Lewis acid sites of the Ce-Cu-SSZ-13_P catalyst is favourable to the low-temperature NH_3_-SCR.^[12]^


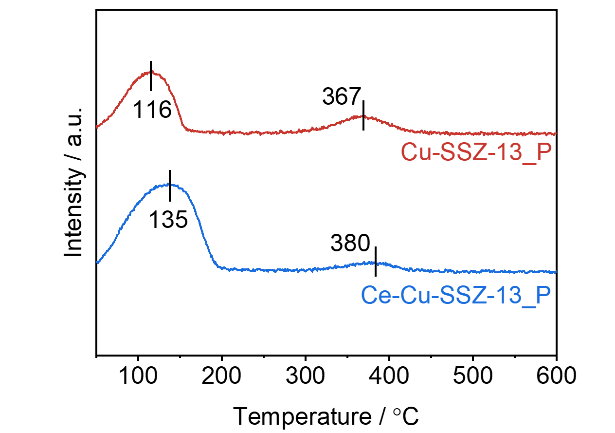


**Figure S44.** NO+O_2_-TPD-MS profiles of the Cu-SSZ-13_P and Ce-Cu-SSZ-13_P catalysts.

The desorption peaks at around 130 °C and 380 °C can be classified into weakly adsorbed NO_x_ and bidentate nitrate.^[13]^


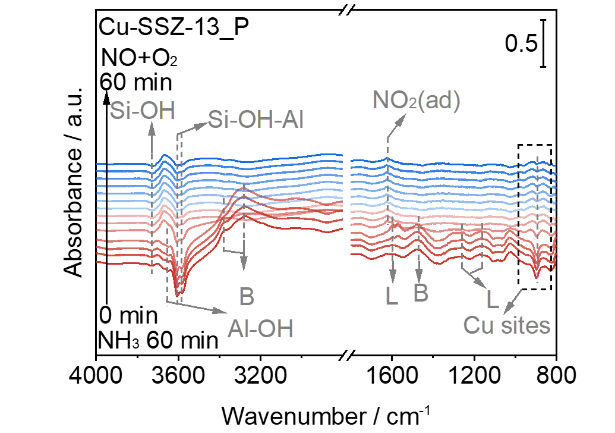


**Figure S45.** *In situ* DRIFTs of the transient reactions between pre-adsorbed NH_3_ and NO + O_2_ as a function of time over the Cu-SSZ-13_P catalyst. The spectra were collected at 180 °C. Experimental conditions: 500 ppm NO, 500 ppm NH_3_ when used, 10 vol % O_2_, and N_2_ as the balance gas.


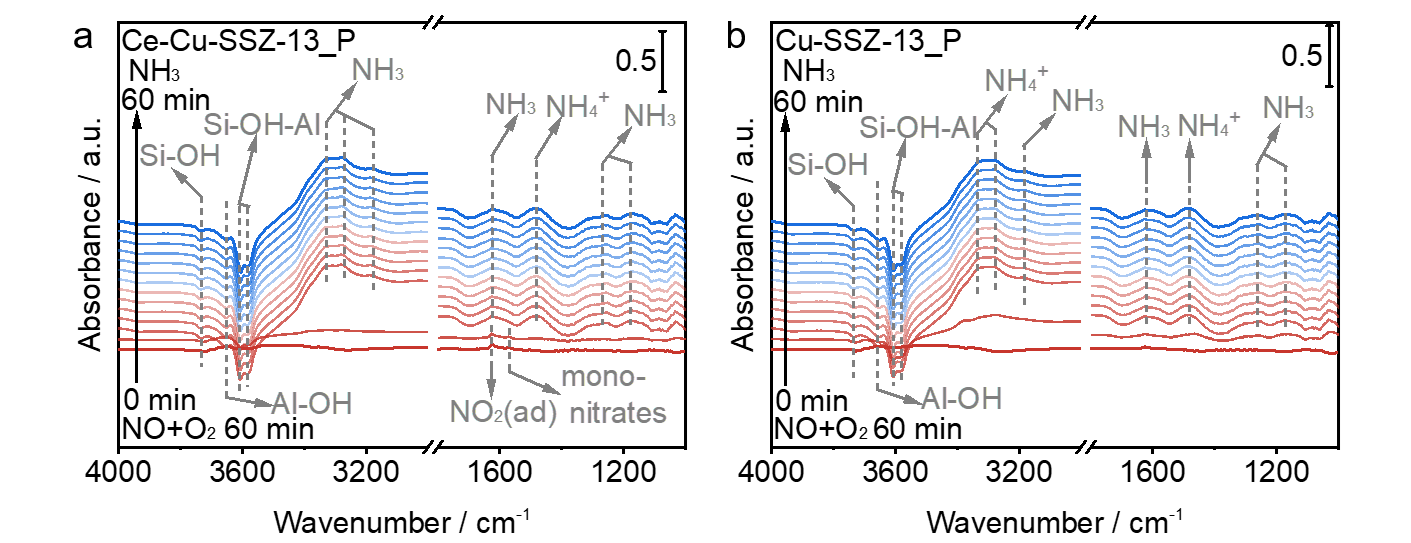


**Figure S46.** *In situ* DRIFTS of the transient reactions between pre-adsorbed NO + O_2_ and NH_3_ as a function of time over the (a) Ce-Cu-SSZ-13_P and (b) Cu-SSZ-13_P catalysts. All the spectra were collected at 180 °C. Experimental conditions: 500 ppm NO, 500 ppm NH_3_ when used, 10 vol % O_2_, and N_2_ as the balance gas.

**Table S1.** Comparison between Ce-Cu-SSZ-13_P with other reported P-tolerant NH_3_-SCR catalysts.

| No. | Catalyst | Test condition | T_99_ / ^o^C | Reference |
| --- | --- | --- | --- | --- |
| 1 | Ce-Cu-SSZ-13_P | [NH_3_] = 500 ppm, [NO] = 500 ppm, [O_2_] = 10%, [H_2_O] = 5%, [CO_2_] = 5%, WHSV=200, 000 mL‧g^−1^‧h^−1^ | 240 | This work |
| 2 | Cu-0.4P-R5 | [NH_3_] = 1100 ppm, [NO] = 1000 ppm, [O_2_] = 5%, [H_2_O] = 10%, GHSV=30, 000 h^−1^ | 450 | ^[6a]^ |
| 3 | Zn&P/Cu | [NH_3_] = 500 ppm, [NO] = 500 ppm, [O_2_] = 5%, GHSV=100, 000 h^−1^ | 300 | ^[12]^ |

**Table S2.** Element contents in different samples from ICP-OES.

| Samples | Cu | Ce | Al | P |
| --- | --- | --- | --- | --- |
|  | wt% | wt% | wt% | wt% |
| Cu-SSZ-13 | 2.37 | - | 2.54 | - |
| Cu-SSZ-13_P | 2.42 | - | 2.18 | 1.14 |
| Ce-Cu-SSZ-13_P | 1.82 | 2.23 | 2.15 | 1.13 |

**Table S3.** Refine atomic position, occupancies, and thermal displacement parameters for the XRD pattern of the Cu-SSZ-13 catalyst. Cu_1_ represent [ZCu^2+^OH]^+^ occupying at 8MRs, Cu_2_ represent Z_2_Cu^2+^ occupying at 6MRs.

| Sample |  | x | y | z | Occ | Uiso | Site |
| --- | --- | --- | --- | --- | --- | --- | --- |
| Cu-SSZ-13 | O_1_ | 0.90200 | 0.09800 | 0.12270 | 1.000 | 0.001 | 18 |
|  | O_2_ | 0.97670 | 0.31010 | 0.16667 | 1.000 | 0.001 | 18 |
|  | O_3_ | 0.12030 | 0.24050 | 0.13150 | 1.000 | 0.001 | 18 |
|  | O_4_ | 0.0000 | 0.25770 | 0.0000 | 1.000 | 0.001 | 18 |
|  | T_1_(Si or Al) | 0.99970 | 0.22640 | 0.10510 | 1.000 | 0.006 | 36 |
|  | Cu_1_ | 0.99676 | 0.41371 | 0.06858 | 0.083 | 0.479 | 36 |
|  | Cu_2_ | 0.0000 | 0.0000 | 0.14779 | 0.028 | 0.800 | 6 |

**Table S4.** Refine atomic position, occupancies, and thermal displacement parameters for the XRD pattern the Cu-SSZ-13_P catalyst. Cu_1_ represent [ZCu^2+^OH]^+^ occupying at 8MRs, Cu_2_ represent Z_2_Cu^2+^ occupying at 6MRs.

| Sample |  | x | y | z | Occ | Uiso | Site |
| --- | --- | --- | --- | --- | --- | --- | --- |
| Cu-SSZ-13_P | O_1_ | 0.90200 | 0.09800 | 0.12270 | 1.000 | 0.000 | 18 |
|  | O_2_ | 0.97670 | 0.31010 | 0.16667 | 1.000 | 0.000 | 36 |
|  | O_3_ | 0.12030 | 0.24050 | 0.13150 | 1.000 | 0.000 | 36 |
|  | O_4_ | 0.00000 | 0.25770 | 0.0000 | 1.000 | 0.000 | 18 |
|  | T_1_(Si or Al) | 0.99970 | 0.22640 | 0.10510 | 1.000 | 0.12 | 36 |
|  | Cu_1_ | 1.0977 | 0.4670 | 0.1018 | 0.0862 | 0.09 | 36 |
|  | Cu_2_ | 0.0000 | 0.0000 | 0.1783 | 0.139 | 0.09 | 6 |

**Table S5.** Refine atomic position, occupancies, and thermal displacement parameters for the XRD pattern the Ce-Cu-SSZ-13 catalyst. Cu_1_ represent [ZCu^2+^OH]^+^ occupying at 8MRs, Cu_2_ represent Z_2_Cu^2+^ occupying at 6MRs, Ce_1_ represent [Z_1_Ce^3+^(OH)_2_]^+^ occupying at 8MRs.

| Sample |  | x | y | z | Occ | Uiso | Site |
| --- | --- | --- | --- | --- | --- | --- | --- |
| Ce-Cu-SSZ-13 | O_1_ | 0.90200 | 0.09800 | 0.12270 | 1.000 | 0.000 | 18 |
|  | O_2_ | 0.97670 | 0.31010 | 0.16667 | 1.000 | 0.000 | 18 |
|  | O_3_ | 0.12030 | 0.24050 | 0.13150 | 1.000 | 0.000 | 18 |
|  | O_4_ | 0.0000 | 0.25770 | 0.0000 | 1.000 | 0.000 | 18 |
|  | T_1_(Si or Al) | 0.99970 | 0.22640 | 0.10510 | 1.000 | 0.011 | 36 |
|  | Cu_1_ | 1.0873 | 0.4678 | 0.09040 | 0.02 | 0.086 | 36 |
|  | Cu_2_ | 0.0000 | 0.0000 | 0.14779 | 0.015 | 0.086 | 6 |
|  | Ce_1_ | 1.0873 | 0.4678 | 0.09040 | 0.015 | 0.086 | 36 |

**Table S6.** Refine atomic position, occupancies, and thermal displacement parameters for the XRD pattern the Ce-Cu-SSZ-13_P catalyst. Cu_1_ represent [ZCu^2+^OH]^+^ occupying at 8MRs, Cu_2_ represent Z_2_Cu^2+^ occupying at 6MRs, Ce_1_ represent [Z_1_Ce^3+^(OH)_2_]^+^ occupying at 8MRs.

| Sample |  | x | y | z | Occ | Uiso | Site |
| --- | --- | --- | --- | --- | --- | --- | --- |
| Ce-Cu-SSZ-13_P | O_1_ | 0.90200 | 0.09800 | 0.12270 | 1.000 | 0.000 | 18 |
|  | O_2_ | 0.97670 | 0.31010 | 0.16667 | 1.000 | 0.000 | 36 |
|  | O_3_ | 0.12030 | 0.24050 | 0.13150 | 1.000 | 0.000 | 36 |
|  | O_4_ | 0.0000 | 0.25770 | 0.0000 | 1.000 | 0.000 | 18 |
|  | T_1_(Si or Al) | 0.99970 | 0.22640 | 0.10510 | 1.000 | -0.52 | 36 |
|  | Cu_1_ | 1.0913 | 0.47690 | 0.08940 | 0.09 | 0.29 | 36 |
|  | Cu_2_ | 0.0000 | 0.0000 | 0.14779 | 0.07 | 0.29 | 6 |
|  | Ce_1_ | 1.0913 | 0.4769 | 0.08940 | 0.067 | 0.29 | 36 |

**Table S7.** EXAFS fitting parameters at the Cu K-edge for the Cu-SSZ-13_P and Ce-Cu-SSZ-13_P catalysts.

| Sample | Path | ^a^N | ^b^R(Å) | ^c^Δσ^2^*10-^3^ (Å^2^) | ^d^ΔE_0_ (eV) | ^e^R factor |
| --- | --- | --- | --- | --- | --- | --- |
| Cu-SSZ-13_P | Cu-O | 4.1 | 1.95±0.01 | 5.7±2.5 | 3.1±1.4 | 0.006 |
|  | Cu-O-Cu | 1.6 | 3.01±0.1 | 10 | 3.1±1.4 |  |
| Ce-Cu-SSZ-13_P | Cu-O | 3.6±0.2 | 1.93±0.005 | -6.3±0.7 | 4.0±0.7 | 0.006 |
|  | Cu-O-Cu | 0.7±0.2 | 2.89±0.02 | -6.3±0.7 | 10 |  |

^a^N: coordination numbers; ^b^R: bond distance; ^c^Δσ^2^*10^-3^ (Å^2^): Debye-Waller factors; ^d^ΔE_0_: the inner potential correction. ^e^R factor: goodness of fit.

**Table S8.** Quantitative analysis result of NH_3_-TPD-MS profiles.

| Catalysts | Weak acid  μmol/g | Lewis acid  μmol/g | Brønsted acid  μmol/g | Tatal acid  μmol/g |
| --- | --- | --- | --- | --- |
| Cu-SSZ-13_P | 6.57 | 0.58 | 3.15 | 10.3 |
| Ce-Cu-SSZ-13_P | 6.01 | 0.91 | 2.86 | 9.78 |

**Movie S1.** AIMD simulation for the PO_4_^3-^ inter-cage diffusion in Ce-Cu-SSZ-13 (MP4). The unit of the continuously changing number in the video multiplied by 10 is fs. (Double click to play the video)

**Movie S2.** AIMD simulation for the PO_4_^3-^ inter-cage diffusion in Cu-SSZ-13 (MP4). The unit of the continuously changing number in the video multiplied by 10 is fs. (Double click to play the video)

# References

[1] G. Kresse, J. Furthmüller, *Phys. Rev. B* **1996**, *54*, 11169-11186.

[2] J. P. Perdew, K. Burke, M. Ernzerhof, *Phys. Rev. Lett.* **1996**, *77*, 3865-3868.

[3] a) B. Himmetoglu, R. M. Wentzcovitch, M. Cococcioni, *Phys. Rev. B* **2011**, *84*, 115108; b) Z. Su, X. Li, W. Si, L. Artiglia, Y. Peng, J. Chen, H. Wang, D. Chen, J. Li, *ACS Catal.* **2023**, *13*, 3444-3455.

[4] S. Ehrlich, *J. Chem. Phys.* **2010**, *132*, 1.

[5] a) A. Wang, K. Xie, D. Bernin, A. Kumar, K. Kamasamudram, L. Olsson, *Appl. Catal. B: Environ.* **2020**, *269*, 118781; b) K. Xie, J. Woo, D. Bernin, A. Kumar, K. Kamasamudram, L. Olsson, *Appl. Catal. B: Environ.* **2019**, *241*, 205-216; c) H. Zhao, Y. Zhao, M. Liu, X. Li, Y. Ma, X. Yong, H. Chen, Y. Li, *Appl. Catal. B: Environ.* **2019**, *252*, 230-239.

[6] a) Z. Chen, C. Bian, Y. Guo, L. Pang, T. Li, *ACS Catal.* **2021**, *11*, 12963-12976; b) I. Popescu, I.-C. Marcu, *Phys. Chem. Chem. Phys.* **2021**, *23*, 5897-5907.

[7] B. Wang, X. Feng, Y. Xu, J.-W. Shi, *Sep. Purif. Technol.* **2023**, *315*, 123679.

[8] P. Zhang, A. Chen, T. Lan, X. Liu, T. Yan, W. Ren, D. Zhang, *J. Hazard. Mater.* **2023**, *441*, 129867.

[9] L. Kang, L. Han, J. He, H. Li, T. Yan, G. Chen, J. Zhang, L. Shi, D. Zhang, *Environ. Sci. Tech.* **2018**, *53*, 938-945.

[10] X.-P. Yang, Z.-Z. Wu, Y.-C. Li, S.-P. Sun, Y.-C. Zhang, J.-W. Duanmu, P.-G. Lu, X.-L. Zhang, F.-Y. Gao, Y. Yang, Y.-H. Wang, P.-C. Yu, S.-K. Li, M.-R. Gao, *Nat. Commun.* **2025**, *16*, 2811.

[11] a) Y. Shen, T. Li, J. Yang, A. Wang, L. Wang, W. Zhan, Y. Guo, Y. Guo, *Chem. Eng. J.* **2023**, *473*, 145275; b) L. Ma, Y. Cheng, G. Cavataio, R. W. McCabe, L. Fu, J. Li, *Chem. Eng. J.* **2013**, *225*, 323-330.

[12] K. Song, S. Zhao, Z. Li, K. Li, Y. Xu, Y. Zhang, Y. Cheng, J.-W. Shi, *J. Colloid Interface Sci.* **2023**, *629*, 243-255.

[13] L. Han, M. Gao, C. Feng, L. Shi, D. Zhang, *Environ. Sci. Tech.* **2019**, *53*, 5946-5956.

# Author Contributions

Y.Q.C designed the study, analyzed the data, and wrote the first draft of the manuscript. P.L.W discussed, revised and polished the manuscript. W.Q.Q performed the AMID calculations. Y.J.S performed the DFT calculations. Y.T performed the PDF/XAFS experiments. E.M., Y.B.N, X.N.H, F.L.W, J.Z, D.C.P, X.D and M.X discussed and revised the manuscript. P.L.W, Y.J.L, E.C, and D.S.Z supervised the project and coordinated the manuscript preparation and discussion. D.S.Z conceived the research.
